# A Ship‐in‐a‐Bottle Strategy: Crosslinking Amines and Epoxides inside MOF Pores for Enhanced CO_2_ Capture Performance

**DOI:** 10.1002/adma.202410138

**Published:** 2025-09-01

**Authors:** Jordi Espín, Anita Justin, Alexandre Hueber, Anne Belin, Sanjay Venkatachalam, Himan Dev Singh, Emad Oveisi, Wendy L. Queen

**Affiliations:** ^1^ Institute of Chemical Sciences and Engineering École Polytechnique Fédérale de Lausanne (EPFL) Sion CH‐1951 Switzerland; ^2^ Interdisciplinary Center for Electron Microscopy École Polytechnique Fédérale de Lausanne (EPFL) Lausanne CH‐1015 Switzerland

**Keywords:** amine‐epoxide polymer, crosslinking, cyclability, MOF composite, postcombustion CO_2_ capture

## Abstract

This study introduces a “ship‐in‐a‐bottle” technique to impregnate porous supports with amines via a straightforward in situ polymerization process. Specifically, alkylamines—tris(2‐aminoethyl)amine (TAEA) and tetraethylene pentamine (TEPA)—are crosslinked with epoxides—trimethylolpropane triglycidyl ether (TMPTE) and 1,3‐butadiene diepoxide (BDE)—within the pores of the metal–organic framework (MOF) Cr‐BDC (also MIL‐101(Cr), where BDC = 1,4‐benzenedicarboxylate), producing four distinct MOF‐polymer composites. These composites are subsequently evaluated for their efficacy in postcombustion carbon capture, examining metrics such as CO_2_ capacity, CO_2_/N_2_ selectivity, isosteric heat of CO_2_ adsorption, kinetic breakthrough times, and cyclability. Among the composites, Cr‐BDC‐TAEA‐BDE (branched‐linear) demonstrates the most promising results, achieving a CO_2_ capacity of 2.2 mmol g^−1^ at 0.15 bar and 313 K, a CO_2_/N_2_ selectivity of 301, and an isosteric heat of CO_2_ adsorption of −110 kJ mol^−1^. This composite also exhibits superior breakthrough performance, with N_2_/CO_2_ separation times of 103 and 143 min per gram under dry and humid conditions, respectively. Furthermore, the four MOF‐polymer composites are subjected to up to 100 temperature swing adsorption/desorption cycles (at 313 and 393 K, respectively), revealing minimal amine leaching or degradation over time. Notably, the composites also show significantly enhanced cyclability compared to Cr‐BDC impregnated with amines without epoxide crosslinking agents; this indicates that crosslinking inhibits amine leaching.

## Graphical abstract

A “ship‐in‐a‐bottle” strategy is used to grow oligomers inside of a MOF by crosslinking alkylamines with epoxides within the pores of Cr‐BDC. When tested in postcombustion carbon capture applications, the composites show excellent performance with high CO_2_ adsorption capacity, exceptional CO_2_/N_2_ selectivity, and enhanced stability over 100 adsorption/desorption cycles. The crosslinking strategy effectively prevents amine leaching and degradation, making the composites more stable than the MOF containing non‐crosslinked alkylamines amines.

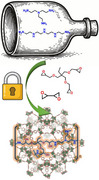

## 1 Introduction

Atmospheric CO_2_ levels are rising at an alarming rate, primarily due to the ongoing combustion of fossil fuels to meet the world's energy demands.^[^ ^1^, ^2^ ^]^ Although transitioning to renewable energy sources is the most appropriate strategy to address this issue, fossil fuels still account for ≈80% of global energy supply.^[^ ^3^, ^4^ ^]^ In addition, it is expected that energy demand will continue to increase at a pace larger than the growth of renewables.^[^ ^5^ ^]^ When considering this and that energy transitions are historically slow, it is projected that fossil fuel use will persist for years to come. Therefore, to reach the NetZero emission target by 2050, it is necessary to implement 2 strategies in parallel; (i) point source carbon capture to avoid further release of CO_2_ to the atmosphere from major contributors such as cement and steel industries, and combustion‐based power plants, and (ii) the deployment of negative emission technologies, which focus on removing CO_2_ already present in the atmosphere, including both natural (reforestation, enhanced rock weathering) and technical solutions (direct air capture (DAC)).^[^ ^6^, ^7^ ^]^

The most mature technology for postcombustion carbon capture (PCC) involves the use of aqueous alkanolamine solutions, commonly known as amine scrubbers.^[^ ^8^ ^]^ Importantly, amines can chemisorb CO_2_ via a nucleophilic attack on the carbon of the CO_2_ molecule, forming either a carbamate or a bicarbonate species.^[^ ^9^, ^10^, ^11^ ^]^ Despite their high selectivity for CO_2_ in typical PCC streams (12–16% CO_2_, 73–77% N_2_, 5–7% H_2_O, 3–4% O_2_, and trace impurities at 1 bar and 313 K or higher), amine scrubbers also face limitations. These include high CO_2_ adsorption enthalpies^[^ ^8^ ^]^ and high heat capacities (3–4 J K^−1^ g^−1^),^[^ ^12^ ^]^ which lead to large energy penalties that can reduce power plant efficiency by as much as 30%.^[^ ^13^ ^]^ Additionally, amine solutions are often highly corrosive and can exhibit poor thermal stability at the temperatures required for CO_2_ release (typically 393–473 K).^[^ ^14^, ^15^ ^]^ As an alternative, porous solid adsorbents are considered promising due to their lower heat capacities (<1 J K^−1^ g^−1^), which could reduce parasitic energy losses by at least half.^[^ ^11^, ^12^ ^]^

Among solid adsorbents, MOFs^[^ ^16^ ^]^ stand out due to their exceptional surface areas, pore volumes,^[^ ^17^ ^]^ and highly tunable porous structures.^[^ ^18^ ^]^ A remarkable number of review articles have been published, underscoring an extensive amount of research focused on MOF development for carbon capture applications.^[^ ^19^, ^20^, ^21^, ^22^, ^23^, ^24^ ^]^ To optimize MOFs for such applications, two prominent strategies have been used: designing MOFs with open metal coordination sites (OMSs)^[^ ^25^, ^26^ ^]^ and/or small pores^[^ ^27^, ^28^, ^29^ ^]^ as both approaches can result in high CO_2_ capacities at low pressures. For instance, a pioneering study of Yaghi et al. demonstrated that Mg‐MOF‐74, a MOF replete with OMSs, exhibits a CO_2_ capacity of ≈6.2 mmol at 0.2 bar and 298 K, resulting in an impressive separation performance of ≈8.9 wt% dynamic capacity in breakthrough experiments (20% CO_2_ in CH_4_).^[^ ^25^ ^]^

Despite the remarkable performance, the efficacy of MOFs with OMSs and small pores can sometimes be problematic in humid environments due to H_2_O competing for CO_2_ adsorption sites and/or framework degradation, which stems from the displacement of metal‐ligand coordination bonds.^[^ ^30^, ^31^ ^]^ To address this, postsynthetic MOF modification (PSM) with amines has been employed on hydrolytically stable MOFs.^[^ ^24^ ^]^ Reported strategies include coordinating amine‐containing molecules to OMSs,^[^ ^32^, ^33^, ^34^, ^35^, ^36^, ^37^, ^38^, ^39^ ^]^ grafting amines onto MOF ligands,^[^ ^40^, ^41^ ^]^ or the wet impregnation of MOF pores with amine‐containing molecules^[^ ^42^, ^43^ ^]^ or polymers.^[^ ^44^, ^45^, ^46^, ^47^, ^48^ ^]^ Several notable studies have explored the PSM of MOFs with amines. For instance, Yaghi et al. presented two strategies for incorporating amines into MOF‐808. The first approach involved decorating MOF‐808 with amino acids via the coordination of carboxylate groups to the zirconium oxo‐cluster. The second strategy employed the coordination of 3‐chloropropionic acid to the zirconium oxo‐cluster, allowing alkyl amine to be subsequently grafted into the MOF pores.^[^ ^39^ ^]^ Two MOF‐808 variants, one containing lysine and the other tris(3‐aminopropyl)amine, exhibited enhanced CO_2_ capacities of ≈1.6 and 1.05 mmol g^−1^ (at 0.15 bar and 298 K), respectively, compared to the parent material.

Long et al. have conducted extensive research on appending amines to OMSs in MOFs.^[^ ^32^, ^37^, ^49^, ^50^, ^51^ ^]^ For example, by appending *N*,*N*′‐dimethylethylenediamine to OMSs in Mg_2_(dobpdc) (dobpdc^2−^ = 4,4′‐dioxidobiphenyl‐3,3′‐dicarboxylate), a CO_2_ capacity of ≈3.14 mmol g^−1^ (at 0.15 bar and 313 K) was achieved. This work also revealed a fascinating cooperative adsorption effect, where CO_2_ insertion between Mg^2+^ and the appended amine led to the formation of ammonium carbamate chains along the MOF's pore wall.^[^ ^32^ ^]^

Jones et al. explored wet impregnation strategies to incorporate tris(2‐aminoethyl)amine and a low‐molecular‐weight PEI‐800 (polyethyleneimine) into MIL‐101(Cr) (also Cr‐BDC).^[^ ^48^ ^]^ Among the two, MIL‐101(Cr)‐PEI‐800 demonstrated superior performance in multicycle temperature swing adsorption experiments, attributed to the lower volatility of the PEI polymer. Cr‐BDC loaded with 1.06 mmol PEI/g exhibited a CO_2_ adsorption capacity of ≈2.5 mmol g^−1^ (at 0.15 bar and 298 K).

Despite these impressive CO_2_ adsorption capacities, amine‐infused porous supports often degrade during adsorption/desorption cycles due to irreversible CO_2_ binding and/or amine displacement from the framework surface, followed by subsequent leaching.^[^ ^48^, ^52^, ^53^, ^54^, ^55^, ^56^ ^]^ To combat this, researchers have explored capping agents like epoxides.^[^ ^52^, ^57^, ^58^, ^59^ ^]^ For example, Choi et al. synthesized PEI capped with 1,2‐epoxybutane (EB), which was then impregnated into silica beads using methanol. This material achieved a CO_2_ capacity of ≈2.2 mmol g^−1^, retained over 50 consecutive temperature swing cycles in a simulated flue gas mixture (adsorption/desorption cycles, 313/393 K).^[^ ^52^, ^59^ ^]^ Accelerated aging tests also indicated that epoxide capping successfully reduced the formation of urea species, a common amine degradation product.

To minimize amine leaching, other strategies have focused on better immobilizing amines within the pores of the support. For example, Long et al. demonstrated that modifying the dimensions of alkylamines to longer tetraamines allowed the amines to bridge between neighboring OMSs. Multiple metal coordination points minimized amine leaching and significantly enhanced long‐term cyclability in humid conditions.^[^ ^33^ ^]^ Further, our group covalently grafted alkylamines onto –NH_2_‐containing ligands in Cr‐BDC‐NH_2_ (also known as NH_2_‐MIL‐101(Cr)) using bromoacetyl bromide as a bridging molecule. While this modification only led to modest CO_2_ capacities of 1.0 and 1.55 mmol g^−1^ at 0.15 bar and 313 K after grafting tris(2‐aminoethyl)amine (TAEA) and PEI‐800, respectively, the composites maintained high CO_2_ capacity even after 200 temperature swing adsorption/desorption cycles. Further, there was no CO_2_ capacity loss after soaking the composite in water. In contrast, when the grafting was replaced with traditional wet impregnation methods, an ≈85% CO_2_ capacity loss was observed when soaked in water.^[^ ^41^ ^]^ However, one issue encountered was the inability to achieve 100% conversion. Unfortunately, only ≈45% of the MOF ligands were functionalized with amines. This could be due to amines grafted to multiple ligands and/or the blocking of MOF pores by grafted amines, inhibiting their diffusion throughout the material and leading to lower‐than‐expected CO_2_ capacities.

To overcome this shortfall, herein, we present a “ship‐in‐a‐bottle” method for amine impregnation inside of a robust MOF, MIL‐101(Cr) (Cr‐BDC = Cr_3_O(OH)(H_2_O)_2_(BDC)_3_ ). For this, the MOF was impregnated with short‐chain alkylamines, which were subsequently crosslinked with di‐ or tri‐epoxides inside the pores. This approach is advantageous because the crosslinking between monomers formed in situ are expected to: (i) better trap the alkylamines in the MOF pores compared to standard wet impregnation methods, reducing amine loss during cycling, (ii) reduce the number of primary amines (after the reaction with epoxides), promoting improved stability and easier regeneration of the composite over many temperature swing adsorption/desorption cycles, and (iii) provide more homogeneous composites (and less pore blocking) relative to those prepared using preformed polymers because it is easier for small monomers to diffuse into the MOF pores.

In this work, two different alkylamines – branched, tris(2‐aminoethyl)amine (TAEA) and linear, tetraethylene pentamine (TEPA) – were crosslinked with two different epoxides: a branched, trimethylolpropane triglycidyl ether (TMPTE) and linear, 1,3‐butadiene diepoxide (BDE), leading to the formation of four distinct oligomers inside of the Cr‐BDC framework. The oligomers included branched‐branched (TAEA‐TMPTE), linear‐linear (TEPA‐BDE), branched‐linear (TAEA‐BDE) and linear‐branched (TEPA‐TMPTE) structures.

We meticulously optimized the reagent ratios, concentrations, reaction time, and temperature to achieve the best results. The CO_2_ adsorption performance of all synthesized materials was then assessed through adsorption measurements, yielding capacities ranging from 1.39 to 2.23 mmol g^−1^ at 313 K and 0.15 bar. Additionally, we calculated the isosteric heats of CO_2_ adsorption from variable temperature data and determined the selectivity via ideal adsorbed solution theory (IAST). To further evaluate performance, breakthrough tests were conducted under both dry and highly humid conditions (15% CO_2_ and 85% N_2_ at 313 K, ≈80% RH in humid experiments). We also subjected the composites to up to 100 temperature swing (313–393 K) adsorption/desorption cycles via thermogravimetric analysis (TGA). After composite exposure to dry CO_2_ at 393 K for extended periods of time, there is no evidence of urea formation indicating the crosslinked materials exhibit high chemical stability. For one of the best performing composites, we further challenged the material to 1000 cycles under humid conditions. Last, the polymers forming within the MOF were characterized using ^1^H NMR and ESI‐MS analyses after composite digestion.

## 2 Results and Discussion

### 2.1 Screening of Reaction Conditions

Cr‐BDC was chosen as the adsorbent for this study due to its remarkable stability, extensive surface area (3450 m^2^ g^−1^), significant pore volume (1.66 cm^3^ g^−1^), and desirable cage sizes (1.2 and 1.6 nm windows with 2.9 nm and 3.4 nm diameters). These characteristics are ideal for promoting effective amine encapsulation.^[^ ^60^, ^61^ ^]^

The synthetic procedure was adapted from the work of Janiak et al.,^[^ ^61^ ^]^ where Cr(NO_3_)_3_·9H_2_O and BDC were dispersed in deionized water, followed by the addition of 1 eq. of HNO_3_ (69%) and heating in an autoclave at 473 K for 16 h. The powder X‐ray diffraction (PXRD) pattern of the resulting MOF matched the simulated one, confirming its structure (Figure S1, Supporting Information). The calculated Brunauer–Emmett–Teller (BET) surface area was 3309 m^2^ g^−1^, and the pore volume 1.65 cm^3^ g^−1^, as determined from N_2_ adsorption isotherms at 77 K (Figures S2 and S3, Supporting Information), aligning closely with previously reported values (3450 m^2^ g^−1^ and 1.6 cm^3^ g^−1^). Further, scanning electron microscopy (SEM) images reveal octahedral crystals having an average size of 1.32 ± 0.25 µm (statistics were calculated using 100 particles of the same sample) (Figure S4, Supporting Information).

**Scheme 1:**
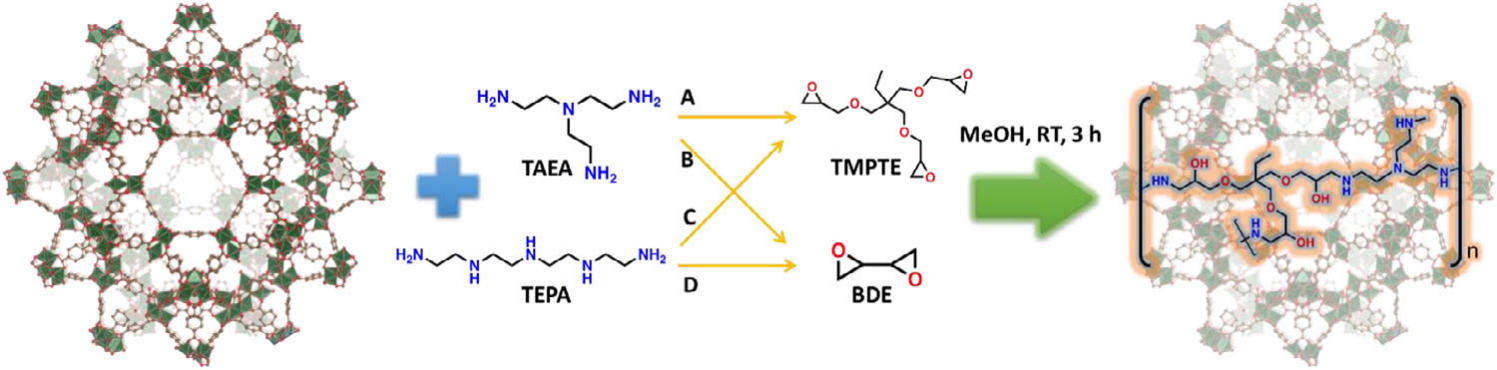
Schematic of the amine‐epoxide combinations and crosslinking approach in the pores of Cr‐BDC (MIL‐101(Cr)). The 4 combinations of amines and epoxides lead to: A = Cr‐BDC‐TAEA‐TMPTE, B = Cr‐BDC‐TAEA‐BDE, C = Cr‐BDC‐TEPA‐TMPTE, and D = Cr‐BDC‐TEPA‐BDE.

The proposed “ship‐in‐the‐bottle” method, used to crosslink amines and epoxides in the MOF pores, consisted of dispersing the MOF in a methanolic solution, and subsequently adding the selected amine and a methanolic solution of the epoxide (**Scheme** **1**). The sample was then removed from the solution via centrifugation, vacuum dried at room temperature, and subsequently heated at 393 K for 12 h under vacuum as a curing step.

To optimize the reaction conditions, TAEA and TMPTE were first used, and several parameters such as reaction time and temperature, solvent volume, and amine/epoxide ratios were varied (Table S1, Supporting Information). First, the temperature (298 K) and time (3 h) were held constant, and the amine: epoxide ratios were varied from 1:1, 2:1, 4:1 also with varying methanol volumes of 2, 4, and 8 mL (entries 1–9 in Table S1 in the Supporting Information). The results indicate the impact of these variables on the CO_2_ capacity at 0.15 bar and 313 K, a pressure and temperature regime relevant to PCC. For example, in the reactions containing low concentration of reagents (8 mL of methanol ‐ MeOH) the highest CO_2_ uptake was ≈1.6 mmol g^−1^ for the 4:1 ratio (entries 1–3 in Table S1 in the Supporting Information); however, in several reactions containing high concentration of reagents (2 mL MeOH), the CO_2_ capacity could not be accurately assessed. The latter stems from significant amount of amine condensation that occurred in the adsorption cell during the activation step (393 K for 12 h under vacuum); this implied that the amine species were leaching from the MOF pores or crosslinking occurred outside (entries 7–9 in Table S1 in the Supporting Information). Therefore, to proceed with the optimization, 4 mL of methanol was selected for the next experiments (entries 4–6 in Table S1 in the Supporting Information). In addition, a notable decrease in the CO_2_ adsorption capacity was observed (0.07–0.54 mmol g^−1^) when using lower amine: epoxide ratios (1:1, 1:2, 1:4) (entries 3, 6, 9, and 10–12 in Table S1 in the Supporting Information), likely due to a large degree of crosslinking (excess of epoxide) and hence, a lower density of amines in the pores was available for CO_2_ adsorption. Given this, an amine: epoxide ratio of 4:1 was selected since all samples obtained using this ratio gave rise to higher CO_2_ capacities (entries 5 and 8) and minimal to no amine leaching.

Next, the impact of reaction time (3 or 12 h) and temperature (298 or 323 K) was determined using an amine: epoxide ratio of 4:1 and MeOH volume of 4 mL (entries 13–15 in Table S1 in the Supporting Information). Interestingly, all samples gave rise to high, yet similar CO_2_ capacities (≈2.3–2.4 mmol g^−1^). Unfortunately, upon activation of these 3 composites, there was also notable amine leaching. So, a second epoxide step was added to reactions containing amine: epoxide ratio of 4:1 to improve amine retention in the framework; however, we observed a significative loss of capacity, 1.56 and 1.07 mmol g^−1^ when using TMPTE and BDE, respectively (entries 22–23 in Table S1 in the Supporting Information). Thus, efforts were made to slightly reduce the amine: epoxide ratio to 3.5:1; however, this sample also underwent light amine leaching after activation (entry 21 in Table S1 in the Supporting Information). Last, the amine: epoxide ratio was further reduced to 3:1 (TAEA: TMPTE). Under these conditions, no amine leaching was observed. This reaction protocol was thus selected as the optimal one and was also rerun several times to demonstrate reproducible CO_2_ adsorption capacities of 1.86 and 1.82 mmol g^−1^ (entries 16 and 20, respectively in Table S1 in the Supporting Information).

Once the optimum reaction conditions were found for the Cr‐BDC‐TAEA‐TMPTE composite (4 mL MeOH, 298 K for 3 h), the rest of the amine‐epoxide combinations were tested under the optimized conditions (Scheme 1). Notably, given the difference in the structures of the monomers, the ratio of the crosslinking species was also screened. From the CO_2_ adsorption isotherms at 313 K, the best amine: epoxide ratios were found to be 3:1, 2:1, and 3:1 for TEPA‐BDE, TAEA‐BDE, and TEPA‐TMPTE composites, respectively (Tables S2–S4, Supporting Information). With the aim to demonstrate reproducibility, three different reactions for each amine‐epoxide combination were carried out, and the isotherms showed similar CO_2_ capacity for all repeated reactions (Figures S6–S10 and Table S5, Supporting Information). Noteworthy, the PXRD patterns of each Cr‐BDC‐amine‐epoxide composite match well with the expected PXRD pattern of Cr‐BDC, confirming the stability of the MOF under the conditions used for in‐situ polymerization reactions. It is noted that there was a change in the relative intensity ratios between the peaks at 2.8° (plane 2 2 0), 3.3°, and 3.4° (planes 3 1 1 and 2 2 2) and a drop in the intensity of the peaks at 5.6° and 5.9° (planes 4 4 0 and 5 3 1), which are likely attributed to pore filling (Figure S17, Supporting Information) as indicated previously in the literature.^[^ ^35^ ^]^

**Figure 1:**
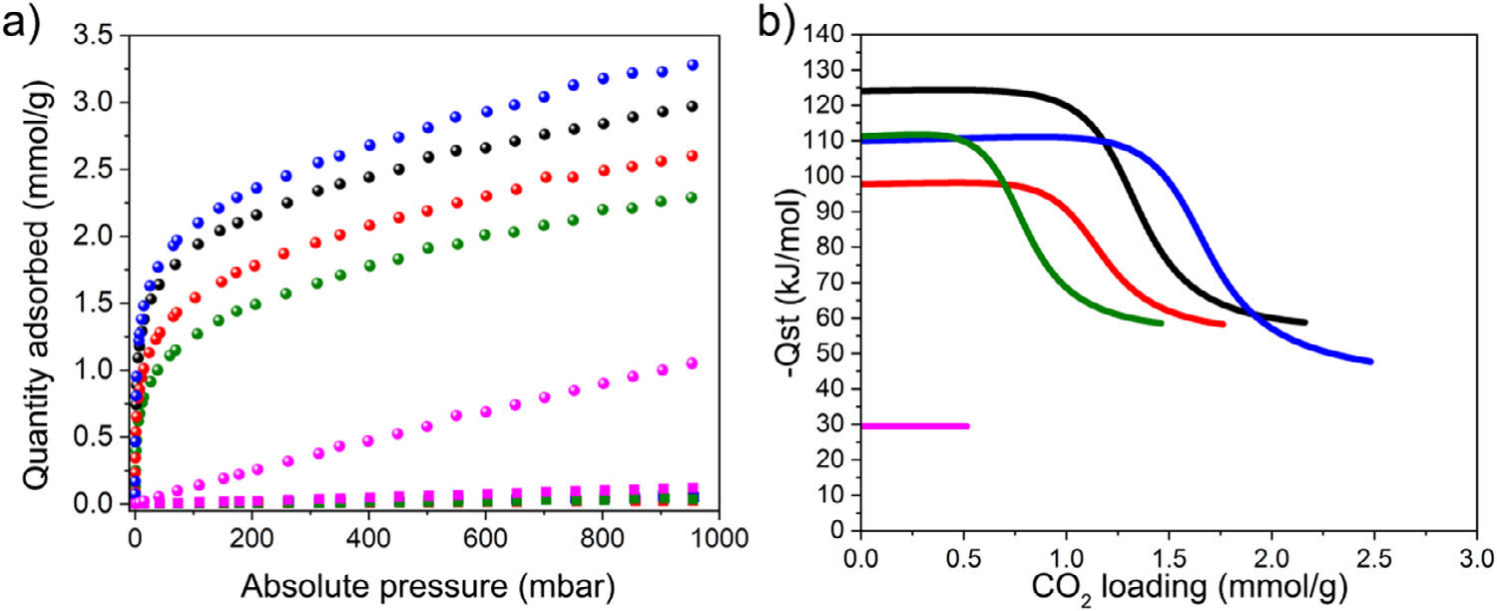
Plot of the a) CO_2_ (spheres) and N_2_ (squares) adsorption isotherms at 313 K, and b) isosteric heat of CO_2_ adsorption of the bare Cr‐BDC and scaled‐up composites; Cr‐BDC (magenta), Cr‐BDC‐TAEA‐TMPTE (black), Cr‐BDC‐TEPA‐BDE (red), Cr‐BDC‐TAEA‐BDE (blue) and Cr‐BDC‐TEPA‐TMPTE (green). The synthesis of these materials was done at larger scales (550 mg).

The scalability of the approach was also considered in this study; hence, the four composites were scaled up by a factor of ten; for this, 550 mg batches of Cr‐BDC were used for composite formation. Notably, the scaled composites have a CO_2_ capacity (313 K) similar to the small‐scale batches (**Figure** **1**a; Table S5, Supporting Information). Only the CO_2_ capacity of Cr‐BDC‐TEPA‐TMPTE composite decreased slightly upon scaling by a factor of ≈19% (**Table** **1**; Table S5, Supporting Information). Considering this, and the need to ensure consistency in the samples, all subsequent studies were carried on composites produced from this larger scale reaction. In addition, reproducibility was also tested using different batches of Cr‐BDC, synthesized by different collaborators in the laboratory (Figures S11–S14, Supporting Information). Last, the scale of the Cr‐BDC‐TAEA‐TMPTE composite was further increased by 100‐times, using 5.5 g of starting Cr‐BDC treated with 100 times more solvent and reagent amounts under the optimized reaction conditions (Figure S15 and Experimental details in the Supporting Information). The CO_2_ adsorption isotherm obtained from the resulting composite is comparable to that of the reaction done on the 550 mg scale (Figure S16, Supporting Information).

**Table 1:** Summary of the CO_2_ and N_2_ capacity at 313 K, and the calculated selectivity and enthalpy of adsorption of all the composites synthesized at larger scales (550 mg starting MOF) and the bare Cr‐BDC.

Sample	CO_2_ adsorption @ 0.15 bar [mmol g^−1^]	CO_2_ adsorption @ 1 bar [mmol g^−1^]	N_2_ adsorption @ 0.85 bar [mmol g^−1^]	Selectivity CO_2_/N_2_ (15/85)	Enthalpy of adsorption at zero coverage, *Q*_st_ [kJ mol^−1^]
Cr‐BDC‐TAEA‐TMPTE	2.05	2.97	0.049	237	−124
Cr‐BDC‐TEPA‐BDE	1.69	2.60	0.023	416	−98
Cr‐BDC‐TAEA‐BDE	2.23	3.29	0.042	301	−110
Cr‐BDC‐TEPA‐TMPTE	1.39	2.29	0.039	202	−111
Bare Cr‐BDC	0.19	1.06	0.10	11	−30

### 2.2 Composites Characterization

The BET surface area, S_BET_, of the four composites, calculated from the measured N_2_ isotherms at 77 K (Figure S18, Supporting Information), shows an expected drop ranging from 63% to 74% (1234–866 m^2^ g^−1^) after the crosslinking step when compared to the parent Cr‐BDC (3309 m^2^ g^−1^) (Table S6, Supporting Information). Surprisingly, the composites containing the linear amine, TEPA, (Cr‐BDC‐TEPA‐TMPTE and Cr‐BDC‐TEPA‐BDE) have a lower S_BET_ of 866 and 894 m^2^ g^−1^, respectively, when compared to those of the branched amine, TAEA, (Cr‐BDC‐TAEA‐TMPTE and Cr‐BDC‐TAEA‐BDE) of 1126 and 1234 m^2^ g^−1^, respectively; however, they all retain their crystallinity postfunctionalization (Figure S17, Supporting Information). Given the decrease in the composite surface areas and shift in the pore size distribution to lower values (Figure S19 and Table S6, Supporting Information), it is expected that the amine and epoxide species have been incorporated inside the MOF pores and are likely filling the largest pores. The pore volume of the parent Cr‐BDC (1.65 cm^3^ g^−1^) decreases after the modification by 65–75% (0.41–0.57 cm^3^ g^−1^) (Table S6, Supporting Information).

To assess whether the polymer was coating the external surface of the MOF crystals or adsorbed in the pores, SEM images were obtained for all four composites (Figures S20–S23, Supporting Information). These images indicated that the MOF crystals retain sharp edges, further confirming that significant amounts of polymeric species do not accumulate on the external surface of the crystals. Further, there is also no evidence of polymer aggregation independent of the MOF crystals. This was further supported by energy dispersive X‐rays spectroscopy (EDXS) in scanning transmission electron microscopy (STEM) of MOF crystals that were embedded in an epoxy resin and serially sliced using ultramicrotomy. The amine‐containing polymer was found throughout the internal surface of the crystals, as indicated by a significant increase in the nitrogen signal throughout the composites (Figures S25–S28, Supporting Information). It is noted that only a faint nitrogen EDXS signal is observed for the bare MOF, Cr‐BDC, which could stem from the residual N,N‐dimethylformamide (DMF) solvent or nitrates in the pores. (Figure S24, Table S8, Supporting Information). However, it is noted that the magnitude of this signal also matches the intensity of the background, attributed to the resin supporting the sliced crystals. Thermogravimetric analysis (TGA) data collected from RT up to 1073 K under air, shows a remarkable drop in the residual weight percentage for all four composites, when compared to the bare MOF (Figures S29 and S30, Supporting Information); this is attributed to crosslinked amine‐epoxide and hence, organics present in the framework pores. The organic content was estimated to be between 39% and 43% for the four optimized composites (Table S7. See TGA section in the Supporting Information). Further, the elemental analysis (EA) results also showed a higher C % and N % content, which comes from the adsorbed organics in the pores (Tables S8 and S9. See EA section in the Supporting Information).

**Figure 2:**
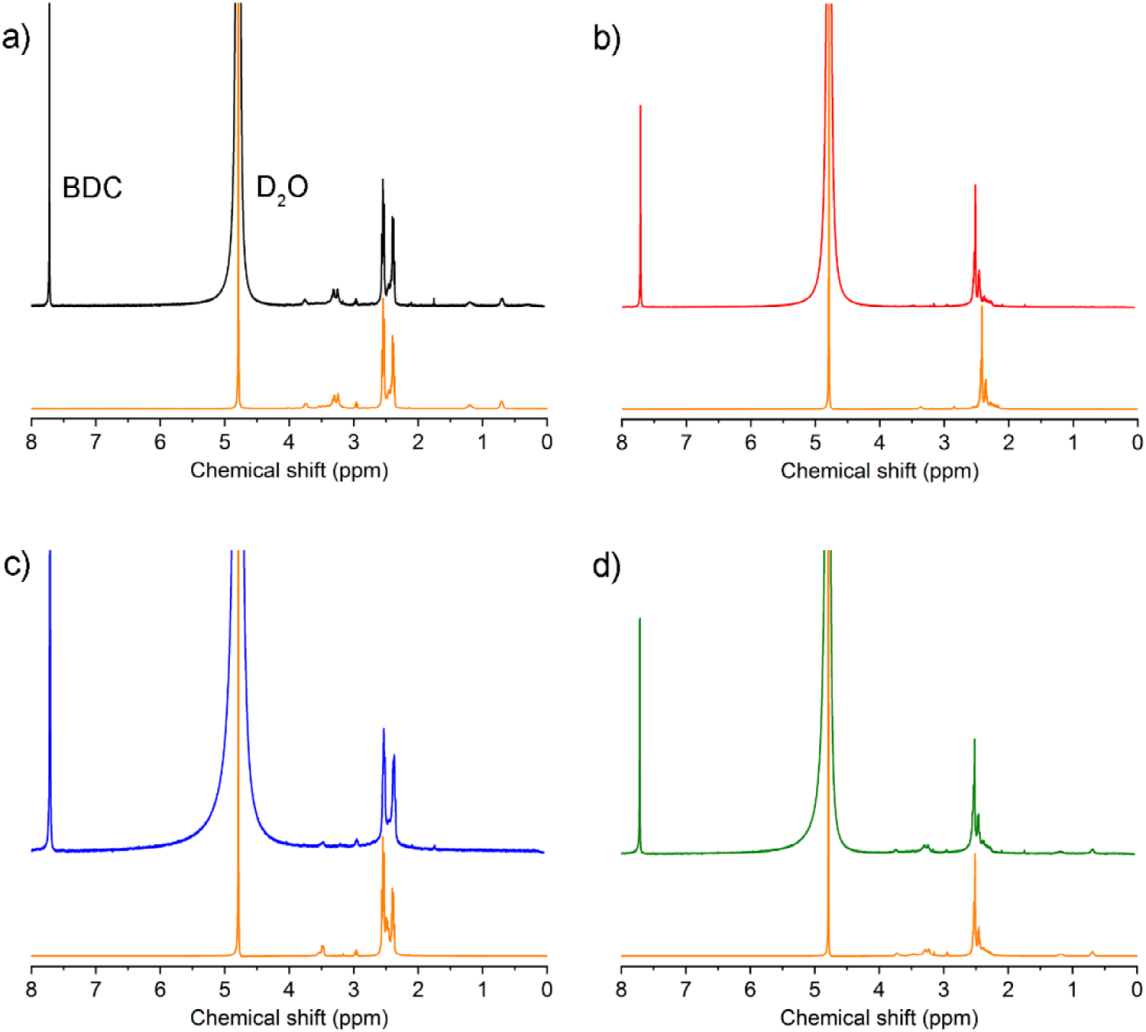
^1^H NMR spectra of the digested a) Cr‐BDC‐TAEA‐TMPTE (black), b) Cr‐BDC‐TEPA‐BDE (red), c) Cr‐BDC‐TAEA‐BDE (blue) and Cr‐BDC‐TEPA‐TMPTE (green), compared to their analogous bulk polymers (orange). The digestion of the samples in both MOF composites and bulk polymers was done using 60 µL NaOD 40% in D_2_O in 500 µL D_2_O. D2O signal is observed at 4.79 ppm and BDC ligand in the digested MOF samples at 7.71 ppm.

To understand the nature of the polymerized species formed during the crosslinking reaction, a control experiment was carried out to form the bulk polymers using the same reaction procedures but excluding the MOF (see Experimental details). During the reaction, the liquid became highly viscous, like honey (Figure S31, Supporting Information). Interestingly, after heating the bulk polymers under vacuum (393 K for 12 h), to mimic the activation process of the MOF‐polymer composites, there was a hardening of the material forming a rocky solid; this confirms that such a curing step helps in crosslinking the amines and epoxides and is likely key to prevent the leaching of low‐molecular weight oligomers from the MOF pores (Figure S32, Supporting Information). After curing, the four control polymers were dissolved in D_2_O and analyzed via ^1^H NMR spectroscopy. Notably, the peaks associated with the protons of the epoxide rings (2.90–3.15 ppm in BDE and 2.75–3.00 ppm in TMPTE) disappeared after being mixed with the amines (Figures S33–S40, Supporting Information) due to the ring‐opening reaction, and thus amine‐epoxide crosslinking. In addition, the peaks related to the pendant ethyl group of TMPTE epoxide (0.88 and 1.38 ppm, for –CH_3_ and –CH_2_–, respectively) as well as the other proton signals of TMPTE between 3.30 and 4.10 ppm, become significantly broadened after the reaction with both TEPA and TAEA amines, indicating that the original molecule loses its symmetry (Figures S37 and S40, Supporting Information). Remarkably, in the case of the reaction of TAEA with either BDE or TMPTE, there are several signs of the amine‐epoxide reaction. First, the main signals of TAEA centered at 2.56 and 2.72 ppm (related to –CH_2_–CH_2_–) indicate a broadening and hence again a loss of symmetry. Moreover, there is a new signal centered at 2.62 ppm that arises from the crosslinking; this is attributed to new –CH_2_– groups that are formed when the epoxide rings open (Figures S37 and S38, Supporting Information). Next, the bulk polymers, were dissolved in the NaOD/D_2_O mixture (60 µL NaOD 40% in D_2_O in 500 µL D_2_O), which are the required conditions to digest Cr‐BDC and the respective composites, to assess whether the basic conditions impact the polymer structure. However, all proton signals only shift slightly up field, thus confirming that the polymer structure was retained under the digestion conditions (Figures S43–S46, Supporting Information). Next, the four activated Cr‐BDC composites were digested using the same NaOD/D_2_O mixture (60 µL NaOD 40% in D_2_O in 500 µL D_2_O) as the bulk polymers, and the samples were sonicated for 30 min to ensure full dissolution of the MOF. As seen in **Figure** **2** and Figures S47–S50 (Supporting Information), the resulting ^1^H NMR spectra prove the polymeric species formed within the MOF have similar chemical environments as the bulk polymers. In the case of the digested composites, the signal‐to‐noise ratio was lower than in the spectra of the bulk polymer due to lower concentration of polymer in the MOF pores. Also, the signal of the BDC ligand was found in all the digested composite samples at 7.71 ppm (Figure 2; Figures S47–S50, Supporting Information).

Considering that the polymers formed with and without the MOF look similar according to ^1^H NMR spectroscopy, the molecular weight of the polymers was also assessed. For this, electrospray ionization‐mass spectrometry (ESI‐MS) data was collected on the bulk polymers synthesized without the MOF (dissolved in MeOH) and the polymer liberated from the MOF pore using a NaOH/methanol mixture (100 µL NaOH 10 m in 500 µL methanol). Surprisingly, the ESI‐MS spectra of the four bulk polymers, shown in Figures S51–S54 (Supporting Information), indicate low‐MW oligomers, in addition to a signal originating from unreacted amine *m*/*z* signal (147.15 for TAEA and 190.20 for TEPA). For instance, for the branched‐branched bulk polymer, TAEA‐TMPTE, species ranging from trimeric oligomers (TAEA‐TMTPE‐TAEA, *m*/*z* = 594.5) to hexameric oligomers (4(TAEA)‐2(TMPTE), *m*/*z* = 1188.96) could be identified (Figure S51, Supporting Information). For the branched‐linear structure, TAEA‐BDE, oligomers of up to eleven units (6(TAEA)‐5(BDE), *m*/*z* = 1307.1) were observed (Figure S52, Supporting Information), and for the linear–linear reaction, TEPA‐BDE, there were species reaching up to seven units (4(TEPA)‐3(BDE)), *m*/*z* = 1014.9) with a purely linear structure (Figure S53, Supporting Information). Last, the linear‐branched polymer, TEPA‐TMPTE, had oligomer sizes up to five units (3(TEPA)‐2(TMPTE), *m*/*z* = 1201.9) (Figure S54, Supporting Information). When further comparing these ESI‐MS spectra with those of the polymers liberated from the composites, there is a shift towards even lower MW oligomeric species indicating that the MOF pores may restrict the propagation of the polymeric chains. For instance, the branched‐branched Cr‐BDC‐TAEA‐TMPTE composite presented dimeric to tetrameric structures with Na^+^ generating adducts due to its presence in the digestion mixture (TAEA‐TMPTE + Na^+^, *m*/*z* = 471.31, 2(TAEA)‐TMPTE + Na^+^, *m*/*z* = 617.8) (Figure S55, Supporting Information). The branched‐linear Cr‐BDC‐TAEA‐BDE revealed trimeric (2(TAEA)‐BDE + Na^+^, *m*/*z* = 401.33) and tetrameric species (2(TAEA)‐2(BDE), *m*/*z* 465.38) (Figure S56, Supporting Information). In the case of linear–linear, Cr‐BDC‐TEPA‐BDE, higher MW structures were observed; there were trimeric 2(TEPA)‐2BDE + 2Na^+^ (*m*/*z* = 572.7) and hexameric oligomers 3(TEPA)‐3(BDE) + Na^+^ (*m*/*z* = 850.7) observed (Figure S57, Supporting Information). Notably, the longer chains obtained from the purely linear candidate could be due to easier diffusion of TEPA and BDE in the pores of Cr‐BDC. Last, the linear‐branched Cr‐BDC‐TEPA‐TMPTE composite also exhibited small species crosslinked in the pores such as dimeric TEPA‐TMPTE + Na^+^ (*m*/*z* = 513.3) and trimeric 2(TEPA)‐TMPTE (*m*/*z* = 702.9) species (Figure S58, Supporting Information).

### 2.3 CO_2_ Capture Performance of the Composites

Once amine crosslinking with epoxides was demonstrated and the CO_2_ adsorption capacity optimized, each MOF‐polymer composite was further assessed for the PCC application. Flue gas is composed mainly of N_2_ (73–75%) and CO_2_ (≈15%), H_2_O vapor and other trace species (O_2_, NO*_x_*, SO*_x_*). Thus, the CO_2_/N_2_ (15/85) selectivity of each composites was assessed via CO_2_ and N_2_ adsorption isotherms measured at 313 K (Figure 1a; Figure S59, Supporting Information) using the IAST method (see IAST section in the Supporting Information).^[^ ^62^ ^]^ While the bare MOF has a selectivity value of 11, the amine‐epoxide composites offer CO_2_/N_2_ (15/85) selectivity ranging from 202 to 416, which is 20 to 40 times higher than the bare Cr‐BDC (Table 1). This highlights the benefits of amine modifications, which not only favors an enhancement in CO_2_ capacity, but also a significant decrease in the N_2_ uptake.^[^ ^40^, ^41^, ^63^ ^]^ Notably, the adsorption capacity for N_2_ gas at 850 mbar and 313 K was reduced by at least a factor of 2, from 0.10 mmol g^−1^ for the parent Cr‐BDC to 0.02–0.05 mmol g^−1^ for the composites (Table 1). Therefore, it is expected that the composites will present a minimal loss of CO_2_ capacity when exposed to postcombustion streams, where N_2_ is the predominant component (85%). The observed trend in selectivity follows Cr‐BDC‐TEPA‐BDE > Cr‐BDC‐TAEA‐BDE > Cr‐BDC‐TAEA‐TMPTE ∼ Cr‐BDC‐TEPA‐TMPTE >> Cr‐BDC.

The isosteric heat of CO_2_ adsorption (*Q*_st_) of all four composites was also assessed. For this, isotherms, measured at variable temperatures (313, 333, and 353 K) (Figures S60–S71 and Tables S10–S13 in the Supporting Information), were fit with a dual‐site Langmuir model and the Clausius–Clapeyron equation was subsequently used to determine the *Q*_st_. (Section S1, Supporting Information). The *Q*_st_ values of the four composites expectedly fall in the chemisorptive regime, from −98 to −124 kJ mol^−1^ (Table 1), which stems from a nucleophilic attack from the nitrogen of the amine on the carbon of the CO_2_, forming carbamate species in dry conditions.^[^ ^10^, ^11^ ^]^ Notably, the branched‐branched composite (Cr‐BDC‐TAEA‐TMPTE) had the highest *Q*_st_ value −124 kJ mol^−1^, while the linear‐linear composite (Cr‐BDC‐TEPA‐BDE) showed the lowest value −98 kJ mol^−1^. The lower *Q*_st_ likely stems from a higher ratio of accessible secondary amines over primary amines, the former being naturally weaker nucleophiles. Notably, before crosslinking TEPA already has fewer primary amines than TAEA. Moreover, the reactivity of the smaller and less sterically hindered BDE could lead to higher conversion of primary amines to secondary amines relative to TMPTE. The remaining two composites, which include a branched amine and linear epoxide (Cr‐BDC‐TAEA‐BDE) and a linear amine with branched epoxide (Cr‐BDC‐TEPA‐TMPTE) have similar *Q*_st_ values, which are −110 and −111 kJ mol^−1^, respectively, despite a significant difference in the CO_2_ adsorption capacity at 0.15 bar (Table 1). As a reference, the isotherms of the bare Cr‐BDC, were fitted to a single‐site Langmuir model (Figures S72–S74 and Table S14, Supporting Information); as expected, the *Q*_st_ value falls in the physisorption regime, −30 kJ mol^−1^. Thus, the absolute value of the isosteric heat of CO_2_ adsorption has the following trend: Cr‐BDC‐TAEA‐TMPTE > Cr‐BDC‐TEPA‐TMPTE ∼ Cr‐BDC‐TAEA‐BDE > Cr‐BDC‐TEPA‐BDE >> Cr‐BDC.

**Figure 3:**
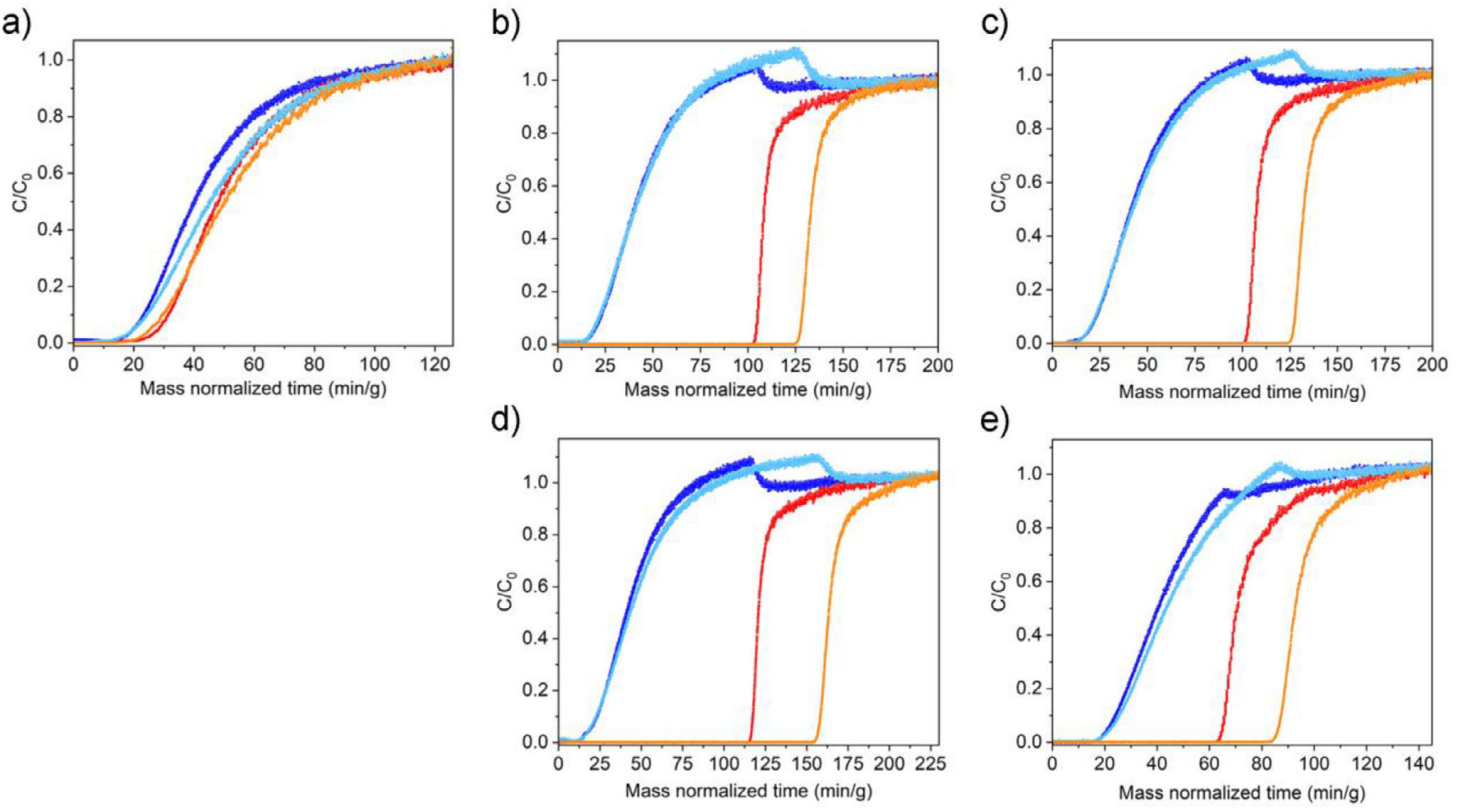
Breakthrough plots under dry (N_2_:CO_2_ 85:15) (N_2_ in blue and CO_2_ in red) and humid conditions (N_2_:CO_2_ 85:15 with 80% RH) (N_2_ in light blue and CO_2_ in orange) of a) the bare Cr‐BDC and the amine‐epoxide composites, b) Cr‐BDC‐TAEA‐TMPTE, c) Cr‐BDC‐TEPA‐BDE, d) Cr‐BDC‐TAEA‐BDE and e) Cr‐BDC‐TEPA‐TMPTE.

While thermodynamic parameters are an important part of assessing a material's potential in PCC applications, the adsorbents will ultimately need to function under continuous flow, thus assessment of their kinetic performance in gas mixtures is pertinent. For that, breakthrough experiments were carried out in a custom‐built apparatus where approximately 300 mg of previously activated sample were mixed with 300 mg of glass beads (108 µm diameter) to minimize the pressure drop throughout the bed. Next, the sample in the bed was purged with He at 313 K and a dry mixture of N_2_ and CO_2_ (85:15) was flowed through the bed (2 mL mi^−1^n), which was held at a temperature of 313 K. The composition of the outlet stream was monitored using a mass spectrometer. Under dry conditions, the N_2_/CO_2_ kinetic separation (time difference between N_2_ and CO_2_ breakthrough after the adsorbent bed) was up to 12 times higher (103 min g^−1^) for the best‐performing composite (Cr‐BDC‐TAEA‐BDE, branched‐linear) when compared to the parent material, Cr‐BDC (8 min g^−1^) (**Figure** **3**a,d). The linear‐branched Cr‐BDC‐TEPA‐TMPTE composite presented the lowest breakthrough separation time (51 min g^−1^) (Figure 3e), and Cr‐BDC‐TAEA‐TMPTE (branched‐branched) and Cr‐BDC‐TEPA‐BDE (linear‐linear) offered intermediate separation times of 87 and 89 min g^−1^, respectively (Figure 3b,c). This trend follows the one observed for the CO_2_ adsorption capacity at 0.15 bar (Table 1), except for the composite Cr‐BDC‐TEPA‐BDE, which exhibits a similar breakthrough time compared to Cr‐BDC‐TAEA‐TMPTE. This could be attributed to the higher CO_2_/N_2_ selectivity of 416 for the linear‐linear Cr‐BDC‐TEPA‐BDE when compared to the branched‐branched Cr‐BDC‐TAEA‐TMPTE composites, 237. Further, Cr‐BDC‐TEPA‐BDE could also exhibit a higher mass transfer coefficient; for example, the linear nature of the entrapped oligomers could ease CO_2_ diffusion through the pores of the MOF support.

**Figure 4:**
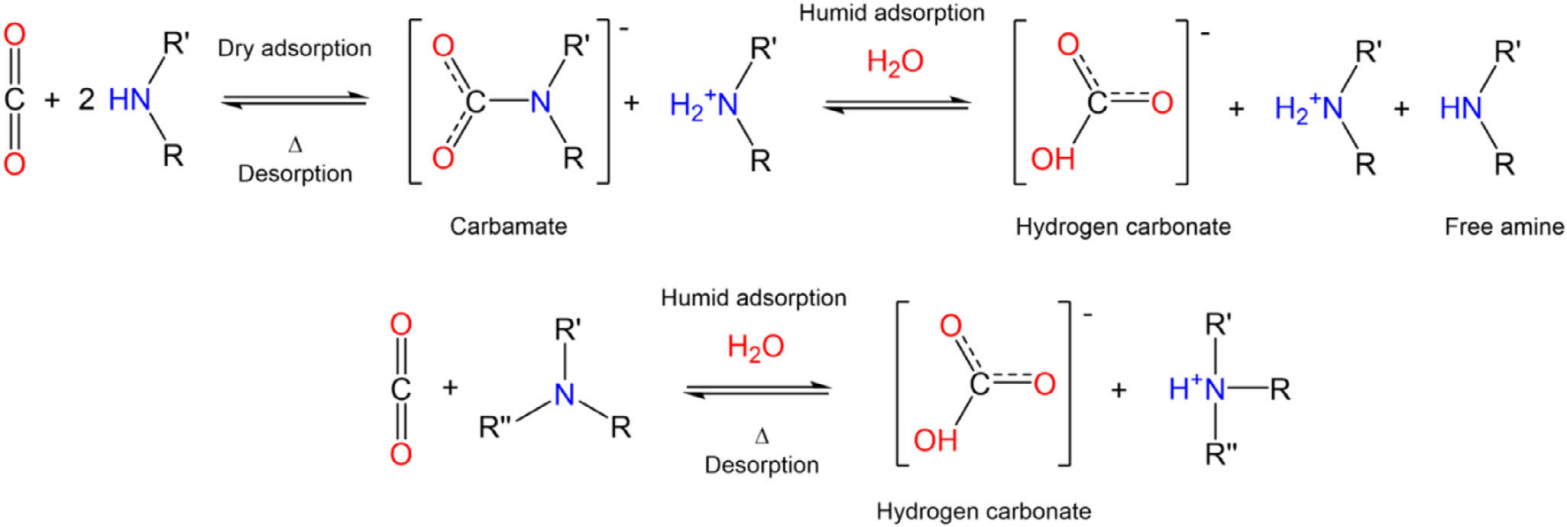
Proposed CO_2_ adsorption mechanism using amines in dry or humid conditions.

Next, breakthrough experiments were carried out for each composite under humid conditions, which included a N_2_: CO_2_ mixture (85:15) containing ≈80 % relative humidity (RH). In this case, the bed was presaturated with 80% RH under He flow for 2 h prior to flowing the gas mixture through the adsorbent column. Under humid conditions the kinetic separation time between N_2_ and CO_2_ was increased for all the composites by 25% to 39%. This enhancement stems from a change in the speciation of the adsorbed CO_2_. In the dry state, there is the predominate formation of carbamate species, which requires an amine: CO_2_ ratio of 2:1; however, humid conditions promote the formation of bicarbonate species, resulting in an amine: CO_2_ ratio of 1:1 (**Figure** **4**),^[^ ^64^ ^]^ increasing the CO_2_ capacity. On the contrary, the breakthrough separation time of the parent Cr‐BDC was reduced by 38 %, due to the competitive binding of water in CO_2_ adsorption sites (Figure 3a). The observed trend in the humid breakthrough experiments is as follows: Cr‐BDC‐TAEA‐BDE > Cr‐BDC‐TEPA‐BDE ∼ Cr‐BDC‐TAEA‐TMPTE > Cr‐BDC‐TEPA‐TMPTE >> Cr‐BDC. Within error of our experiment, this trend is similar to the one observed under the dry conditions. From this work, it seems the use of branched amine (TAEA) is favored in breakthrough experiments when compared to the linear one (TEPA), likely due to the larger number of accessible primary amines. Further, the use of a linear epoxide (BDE) compared to the branched one (TMPTE) seems to yield improved performance; this likely stems from the lower molecular weight of the smaller BDE crosslinking agent, which could boost capacity (Table 1) and/or help to ease the access of CO_2_ to the amines (**Table** **2**).

**Table 2:** Summary of the breakthrough time separation between N_2_ and CO_2_ for the bare MOF and all the composites under dry and humid conditions.

Sample	Breakthrough N_2_/CO_2_ separation time under dry conditions [min g^−1^]	Breakthrough N_2_/CO_2_ separation time under humid conditions [min g^−1^]
Cr‐BDC‐TAEA‐TMPTE	87	110
Cr‐BDC‐TEPA‐BDE	89	111
Cr‐BDC‐TAEA‐BDE	103	142
Cr‐BDC‐TEPA‐TMPTE	51	71
Bare Cr‐BDC	8	5

While breakthrough separation and thermodynamic parameters are an important part of the assessment process for PCC applications, long‐term cyclability in humid environments is equally important. Motivated by the excellent performance of the composites presented here, we evaluated the stability of one composite material, Cr‐BDC‐TAEA‐TMPTE, over 1000 TSA cycles (4–5 weeks) in a CO_2_/N_2_ (15/85) mixture with ≈80% RH (313/393 K adsorption/desorption). The results revealed a flat baseline, indicating minimal to no amine loss or degradation during cycling (Figures S75 and S76, Supporting Information). Additionally, the overall capacity remained effectively unchanged throughout the cycle period. Despite this, the TGA data showed peaks and troughs, indicating ≈2 weight% fluctuations in the quantity of CO_2_ and H_2_O adsorbed (Figures S75 and S76, Supporting Information). To investigate, we measured the temperature of the room housing the TGA during a second set of cycling experiments. Figure S77 (Supporting Information) demonstrated that the fluctuations in the room's temperature profile correlated with the observed fluctuations in adsorption capacity of Cr‐BDC‐TAEA‐TMPTE. These temperature changes directly impacted the humidity level in the gas stream, leading to the fluctuations in the TGA plot. Such fluctuations make it difficult to accurately determine the quantity of CO_2_ present in the sample at any given time. Future TGA cycling experiments should therefore be conducted in a temperature‐ and humidity‐controlled environment. Nonetheless, despite these temperature control issues, the humid cycling experiment indicated minimal capacity loss, suggesting no significant amine loss or degradation with extensive cycling. This consistent performance over time underscores the potential of these new composite materials for future studies aimed at further assessing their carbon capture performance.

To emphasize the importance of the crosslinking approach, we compared the four new amine‐epoxide composites to the Cr‐BDC framework impregnated with TAEA or TEPA, excluding the crosslinking agents. For this, traditional wet impregnation approaches were used in anhydrous cyclohexane or MeOH.^[^ ^35^ ^]^ Importantly, the anhydrous cyclohexane creates a chemical potential difference due to the nonpolar nature of the solvent, promoting more amine impregnation into the Cr‐MOF and appendage to the open Cr^3+^ sites.^[^ ^35^ ^]^ Subsequently, the CO_2_ adsorption isotherms were collected at 313 K from the four materials produced via traditional wet impregnation approaches (Figures S78 and S79 and Table S15, Supporting Information). As expected, materials impregnated using cyclohexane demonstrated a higher capacity at 0.15 bar and 313 K (4.10 mmol g^−1^ for Cr‐BDC‐TAEA and 2.40 mmol g^−1^ for Cr‐BDC‐TEPA) compared to those impregnated in methanol (2.09 mmol g^−1^ for Cr‐BDC‐TAEA and 1.81 mmol g^−1^ for Cr‐BDC‐TEPA) (Table S15, Supporting Information). Also, the branched amine, TAEA, resulted in higher capacities likely due to the higher density of primary amines relative to the linear TEPA.

The adsorption isotherms of the four traditionally impregnated materials were then compared to those of the four Cr‐BDC‐amine‐epoxide composites (Figures S78 and S79 and Table S15, Supporting Information). While the CO_2_ adsorption capacities of the non‐crosslinked Cr‐BDC‐amine materials were comparable or higher than the Cr‐BDC‐amine‐epoxide composites, significant amine leaching was observed on the surface of the glass tubes, rendering the CO_2_ adsorption capacities unreliable—a phenomenon often overlooked in the literature.

**Figure 5:**
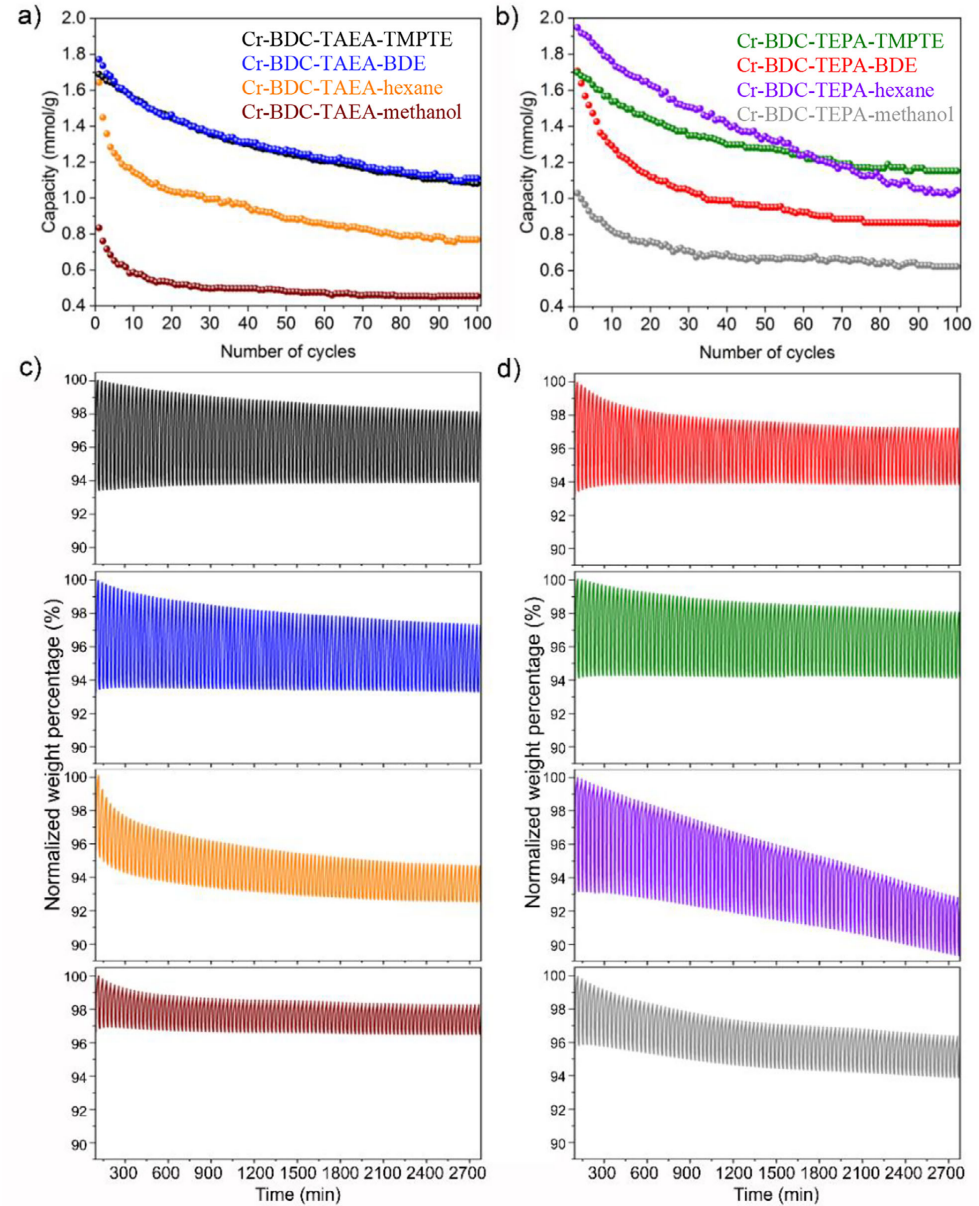
Plots of 100 TSA cycles under 313 K adsorption and 393 K desorption of pure CO_2_ for a) TAEA‐based materials including Cr‐BDC‐TAEA‐TMPTE (black), Cr‐BDC‐TAEA‐BDE (blue), Cr‐BDC‐TAEA in cyclohexane (orange) and Cr‐BDC‐TAEA in methanol (brown), and b) TEPA‐based materials; Cr‐BDC‐TEPA‐BDE (red), Cr‐BDC‐TEPA‐TMPTE (green), Cr‐BDC‐TEPA in cyclohexane (purple) and Cr‐BDC‐TEPA in methanol (grey). Full cycling data shown as normalized weight percentage to each first cycle for c) Cr‐BDC‐TAEA and d) Cr‐BDC‐TEPA composites (same color references as per plots (a) and (b), respectively).

To experimentally demonstrate amine loss, we assessed the cyclable capacity of the eight materials over 100 TSA cycles, consisting of 5‐min adsorption (313 K) and desorption (393 K) cycles in a pure, dry stream of CO_2_ (**Figure** **5**; Figures S80–S95, Supporting Information). These experiments highlighted the importance of the epoxide crosslinking on the long‐term performance. For instance, Cr‐BDC‐TAEA‐cyclohexane and Cr‐BDC‐TAEA‐methanol both present a significantly lower capacity than the Cr‐BTC‐TAEA‐epoxide composites after only a few adsorption/desorption cycles (Figure 5a,c). Importantly, the baselines of the TGA plots of Cr‐BDC‐TAEA‐methanol and Cr‐BDC‐TAEA‐cyclohexane also drop with cycling due to amine loss, reducing the overall mass of the composite after each cycle (Figure 5c; Figures S88,S89,S92,S93, Supporting Information). In contrast, Cr‐BDC‐TAEA‐TMPTE and Cr‐BDC‐TAEA‐BDE have flat baselines in the TGA plots (Figure 5c; Figures S80,S81,S84,S85, Supporting Information), indicating minimal to no amine loss. The final capacities of Cr‐BDC‐TAEA‐cyclohexane and Cr‐BDC‐TAEA‐methanol is only 0.77 and 0.46 mmol g^−1^, respectively, significantly lower than the two polymer composites.

Similar cycling experiments for TEPA‐containing materials revealed comparable trends (Figure 5b,d); for instance, both Cr‐BDC‐TEPA‐methanol and Cr‐BDC‐TEPA‐cyclohexane exhibited plummeting baselines during the TSA cycling experiments (Figure 5d; Figures S90,S91,S94,S95, Supporting Information) demonstrating amine loss. Conversely, Cr‐BDC‐TEPA‐BDE and Cr‐BDC‐TEPA‐TMPTE composites maintained flat baselines (Figure 5d; Figures S82,S83,S86,S87, Supporting Information), with more stable cyclable capacities over 100 adsorption/desorption cycles (Figure 5b). By cycle 100, most of the material's capacities had plateaued; however, Cr‐BDC‐TEPA‐cyclohexane continued to decline, indicating further amine loss.

During the TSA cycling experiments in pure CO_2_, the four Cr‐BDC‐amine‐epoxide composites showed an overall capacity decline (Figure 5a,b). This was unexpected given the flat baselines in the TGA plots (Figure 5c,d), which ruled out significant amine loss (denoted by a downward slope) or the irreversible formation of urea species (denoted by an upward slope). While this capacity decline could stem from several unknown factors, it is noted that throughout the cycling experiments, a N_2_ stream was flowed into the counterbalance of the TGA. If the CO_2_ concentration in the gas stream was diluted by this N_2_ stream, the materials’ capacities would naturally decrease overtime. However, since all materials were tested under the same conditions, each sample would experience the same dilution factor. Nonetheless, these experiments demonstrated that all four amine‐epoxide composites present significantly higher cyclable capacities and insignificant amine loss compared to Cr‐BDC impregnated with TAEA or TEPA using traditional impregnation methods.

To further probe the chemical stability of the amine‐based composites prepared using this new crosslinking method, an accelerated aging test was conducted. Three materials—Cr‐BDC‐TAEA‐BDE and Cr‐BDC‐TAEA‐TMPTE (both crosslinked) and Cr‐BDC‐TAEA (impregnated with amines via cyclohexane)—were exposed to pure CO_2_ at 393 K for 18 h using a TGA. Such conditions are known to promote the formation of urea species,^[^ ^56^, ^65^ ^]^ which cause deactivation of amine‐based sorbents. After exposure, diffuse reflectance infrared Fourier transform spectroscopy (DRIFTS) was used to detect the formation of these urea species (Figure S96, Supporting Information). The spectra of the impregnated sample, Cr‐BDC‐TAEA CH, (containing non‐crosslinked amines) revealed distinct new peaks corresponding to urea functionalities, such as 1567, 1300, and 813 cm^−1^ corresponding to the N–H bending, N–H deformation vibration in NH_2_ and C–N stretching vibration, indicating chemical degradation.^[^ ^66^ ^]^ Furthermore, the band at 1700 cm^−1^ can be attributed to the C═O stretching frequency associated with the formation of carbamate species^[^ ^67^ ^]^ (Figure S96b, Supporting Information). In contrast, the crosslinked composites did not show such peaks, demonstrating that crosslinking significantly enhances the chemical stability of these materials under CO_2_ exposure conditions relative to those produced using traditional impregnation approaches (Figure S96b, Supporting Information).

Last, it is commonly suggested throughout the literature that high isosteric heats of adsorption may potentially lead to high energy cost during the capture process. While the energy cost was not investigated herein as it requires extensive efforts to integrate adsorbents into realistic capture processes combined with modeling (process parameters like productivity, purity and recovery must be thoroughly assessed), we did explore the tunability of the *Q*_st_. Importantly, for the four composites presented above, structurally different amines and epoxides were used. Given that the TGA (Figures S29 and S30 and Table S7, Supporting Information) and EA (Tables S8 and S9, Supporting Information) indicate similarities in polymer loadings in all four composites, it is thought such differences in zero‐coverage *Q*_st_, which range from −98 to −124 kJ mol^−1^, are likely attributed to structural variations in the formed polymer. For example, significant differences in *Q*_st_ were observed between composites with different amines (Figure 1b), such as Cr‐BDC‐TAEA‐BDE (−110 kJ mol^−1^) and Cr‐BDC‐TEPA‐BDE (−98 kJ mol^−1^). This difference stems from TAEA having 3 primary amines compared to 2 for TEPA, resulting in TAEA‐BDE polymers with higher quantities of primary amines and consequently higher *Q*_st_. The same trend appears with Cr‐BDC‐TAEA‐TMPTE (−124 kJ mol^−1^) and Cr‐BDC‐TEPA‐TMPTE (−111 kJ mol^−1^).

To further investigate *Q*_st_ tunability, several new Cr‐BDC‐TAEA‐BDE variants were created by modifying either the amine:epoxide ratio or the polymer content in the MOF pores (Table S16 and Figures S97–S99, Supporting Information). Using the original Cr‐BDC‐TAEA‐BDE (42% polymer content and *Q*_st_ of −110 kJ mol^−1^) as a reference, several composite variants were made. First, doubling the quantity of Cr‐BDC in the amine crosslinking reaction reduced polymer content to 19.9%, accompanied by a dramatic drop in the *Q*_st_ to −55 kJ mol^−1^. This may indicate that the high *Q*_st_ of Cr‐BDC‐TAEA‐BDE containing 42% polymer is driven by interactions between polymer chains. Second, reducing the TAEA concentration to 75% of its original value, decreased the polymer content from 42% to 31.8% and the *Q*_st_ to −79 kJ mol^−1^. Third, increasing the quantity of crosslinking epoxide BDE by 50%, led to an increase in polymer content to 46.5% and a decrease in the *Q*_st_ to −62 kJ mol^−1^, likely due to increased capping of primary amines. This work indicates that the binding strength of CO_2_ in these composites is readily tunable. Thus, in the future, it will be interesting to understand how such variations impact the cyclable CO_2_ capacity of these adsorbents in humid postcombustion flue gas streams.

## 3 Conclusion

In this study, we present a simple and scalable method to crosslink amines and epoxides within the pores of a Cr‐MOF, resulting in high performance adsorbents for postcombustion CO_2_ capture (Table S17, Supporting Information). We employed a robust MOF, Cr‐BDC (also known as MIL‐101(Cr)), as the support for four different amine‐epoxide polymers. These polymers were composed of branched–branched, linear–linear, branched–linear, and linear–branched amine‐epoxide monomers. After optimizing the CO_2_ capacity of each composite, we sought to understand how the different polymeric structures influenced performance in PCC applications. We evaluated the composites based on five criteria: CO_2_ capacity (at 0.15 bar and 313 K), IAST CO_2_/N_2_ selectivity (313 K), isosteric heat of CO_2_ adsorption, breakthrough separation time in highly humid CO_2_/N_2_ mixtures (15/85 and 80% RH at 313 K), and stability over numerous TSA cycles (313/393 K adsorption/desorption).

Our findings concluded that Cr‐BDC‐TAEA‐BDE (branched amine‐linear epoxide) emerged as the top‐performing composite among those tested. It exhibited impressive CO_2_/N_2_ selectivity of 301, an intermediate isosteric heat of CO_2_ adsorption (−110 kJ mol^−1^), a CO_2_ capacity of 2.2 mmol g^−1^, and an outstanding CO_2_/N_2_ separation time of 142 min g^−1^. These metrics represent a 10% and 29% improvement in CO_2_ capacity and breakthrough time, respectively, compared to the second‐best performing composite. Additionally, the composites demonstrated exceptional stability after as many as 1000 TSA cycles (at 313/393K, adsorption/desorption) in humid gas mixtures containing ≈15% CO_2_. Lastly, we showed that this “ship‐in‐a‐bottle” strategy effectively minimizes amine leaching and improves their stability. This significantly enhances cycling performance compared to the same MOF impregnated with amines using traditional wet impregnation methods (excluding epoxide crosslinkers).

It should be noted that to apply these materials to CO_2_ capture from flue gases, one must consider the energy cost and environmental sustainability of each material, assess their performance in the presence of other common impurities found in flue gas streams (e.g., NO_x_ and SO_x_) develop strategies for shaping the fine powders into larger structures (e.g., pellets, beads, or extrudates), and integrate the structured materials into realistic processes that mimic real‐world separations. This, combined with future technoeconomic assessments of the capture cost will allow us to further assess their feasibility for large scale CO_2_ capture efforts.

## 4 Experimental Section

### Materials

All chemicals were obtained from commercial sources and used as received without further purification. Cr(NO_3_)_3_·9H_2_O (99%, 1000 g) was purchased from Acros Organics, 1,4‐benzenedicarboxylic acid (BDC) (99+ %, 500 g) from Acros Organics, HNO_3_ (69%, 1 L) from Roth, tris(2‐aminoethyl)amine (TAEA) (97%, 100 g) from Alfa Aesar, trimethylolpropane triglycidyl ether (TMPTE) (technical grade, 250 mL) from Sigma Aldrich, tetraethylene pentamine (TEPA) (technical grade, 500 g) from Sigma Aldrich, 1,3‐butadiene diepoxide (BDE) (97 %, 25 mL) from Sigma Aldrich, methanol (MeOH) (8 L) from Reactolab, *N*,*N*‐dimethylformamide (DMF) (99 %, 2.5L) from Fisher Chemical, ethanol (EtOH) (8 L) from Reactolab and anhydrous cyclohexane (99.5 %, 1 L) from Sigma Aldrich.

### Synthesis of Cr‐BDC

In a 1 L Teflon jar disperse 52.8 g Cr(NO_3_)_3_·9H_2_O (0.132 mol), 18.0 g BDC (0.108 mol) in 660 mL deionized H_2_O and add 8.56 mL HNO_3_ 69% (0.132 mol, 1 eq respect chromium nitrate). Stir the mixture for 30 min, bring the autoclave to a preheated oven at 473 K and let react for 16 h. Let cool down naturally in the oven for 12 h. Decant the supernatant and collect the solid in six 50 mL falcon tubes by centrifugation for 10 min at 7800 rpm. Discard the supernatant and wash once with 35 mL DMF/falcon tube shaking them for 10 min. Transfer the solid to a 500 mL glass jar, add 200 mL of fresh DMF and stir at 700 rpm for 6 h. Centrifuge the solids, discard the supernatant and repeat this process 3 more times to ensure proper removal of unreacted species. Proceed in a similar manner with ethanol, adding 200 mL of fresh solvent and stirring for 6 h repeating this process 3 times. After that, dry in the vacuum oven at room temperature for 24 h. The material was heated up to 423 K during 2 h and kept at this temperature for 12 h under vacuum prior to N_2_ adsorption isotherm measurement at 77 K for BET surface area calculation.

### Synthesis of Cr‐BDC‐Amine‐Epoxide Composites

Small scale: 55 mg of as‐synthesized Cr‐BDC was dispersed in 2 mL MeOH and the desired amount of amine was added directly to this suspension. Next, the selected amount of epoxide was dissolved in 2 mL MeOH, which was added to the original mixture. The suspension was stirred at 298 K for 3 h (or the desired temperature and time during optimization experiments). Finally, the mixture is centrifuged for 5 min at 7800 rpm and the supernatant is discarded. The resulting solid is vacuum dried at room temperature for 24 h. The material was activated by heating up to 393 K during 1 h and kept at this temperature for 12 h under vacuum prior to CO_2_ and N_2_ adsorption isotherm measurements.

10‐times scale: the original reactions were performed at 10 times larger scales. 550 mg of as‐synthesized Cr‐BDC was dispersed in 20 mL MeOH and the desired amount of amine (see below) was added directly to this suspension using a 100 mL round bottom flask. Next, the selected amount of epoxide (see below) was dissolved in 20 mL MeOH, which was added to the original mixture. The suspension was stirred at 298 K for 3 h. Finally, the mixture is centrifuged for 5 min at 7800 rpm and the supernatant is discarded. The resulting solid is vacuum dried at room temperature for 24 h. The material was activated by heating up to 393 K during 1 h and kept at this temperature for 12 h under vacuum prior to CO_2_ and N_2_ adsorption isotherm measurements, and TSA cycles.

*Amine‐epoxide ratios employed for the 10‐times scale‐up reactions*:

*Cr‐BDC‐101‐TAEA‐TMPTE (3:1)*

4.05 mL TAEA (36 equivalents) were employed as amine source and 2.56 mL TMPTE (12 equivalents) as epoxide source.

*Cr‐BDC‐101‐TEPA‐BDE (3:1)*

5.25 mL TEPA (36 equivalents) were employed as amine source and 0.71 mL BDE (12 equivalents) as epoxide source.

*Cr‐BDC‐101‐TAEA‐BDE (2:1)*

4.05 mL TAEA (36 equivalents) were employed as amine source and 1.07 mL BDE (18 equivalents) as epoxide source.

*Cr‐BDC‐TEPA‐TMPTE (3:1)*

5.25 mL TEPA (36 equivalents) were employed as amine source and 2.56 mL TMPTE (12 equivalents) as epoxide source.

Cr‐BDC‐TAEA‐TMPTE 100‐times scale: the original Cr‐BDC‐TAEA‐TMPTE reaction was performed at 100 times larger scale. 5.5 g of as‐synthesized Cr‐BDC was dispersed in 200 mL MeOH and 40.5 mL TAEA was added directly to this suspension using a 1 L round bottom flask. Next, 25.6 mL of TMPTE was dissolved in 200 mL MeOH, which was added to the original mixture. The suspension was stirred at 298 K for 3 h. Finally, the mixture is centrifuged for 5 min at 7800 rpm and the supernatant is discarded. The resulting solid is vacuum dried at room temperature for 24 h. The material was activated by heating up to 393 K during 1 h and kept at this temperature for 12 h under vacuum prior to CO_2_ and N_2_ adsorption isotherm measurements.

### Synthesis of Bare Polymers

The synthesis of the bare polymers was performed using the same amine‐epoxide ratios, solvent amount, temperature and time as per the composites, but without adding any MOF. In short, a certain amount of amine (see below) was dissolved in 20 mL MeOH in a 100 mL round bottom flask, to which a selected amount of epoxide (see below) dissolved in 20 mL MeOH was added. The solution was stirred at 298 K for 3 h. Next, the mixture is vacuum dried at room temperature for 24 h to obtain a honey‐like polymer. Finally, the gel was activated at 393 K under vacuum for 12 h to complete the crosslinking and obtain a rocky polymer.

*TAEA‐TMPTE (3:1)*

4.05 mL TAEA (36 equivalents) were employed as amine source and 2.56 mL TMPTE (12 equivalents) as epoxide source.

*TEPA‐BDE (3:1)*

5.25 mL TEPA (36 equivalents) were employed as amine source and 0.71 mL BDE (12 equivalents) as epoxide source.

*TAEA‐BDE (2:1)*

4.05 mL TAEA (36 equivalents) were employed as amine source and 1.07 mL BDE (18 equivalents) as epoxide source.

*TEPA‐TMPTE (3:1)*

5.25 mL TEPA (36 equivalents) were employed as amine source and 2.56 mL TMPTE (12 equivalents) as epoxide source.

### Synthesis of Cr‐BDC Amine Impregnated in Cyclohexane

The synthesis of these materials was done as a control to compare the effect of the epoxide crosslinking with the amines inside the MOF pores. This protocol was adapted from the literature.^[^ ^35^ ^]^ Cr‐BDC was previously activated in a Schlenk line at 423 K for 12 h under vacuum. Next, working in the glovebox, 405 µL TAEA in 4 mL of anhydrous cyclohexane were added to 55 mg of activated Cr‐BDC (Cr‐BDC‐TAEA in cyclohexane) or 525 µL TEPA in 4 mL of anhydrous cyclohexane were added to 55 mg of activated Cr‐BDC (Cr‐BDC‐TEPA in cyclohexane). In both cases the amine solution was stirred for only 5 min with the MOF powder and was subsequently centrifuged and washed 3 times with 15 mL cyclohexane outside the glovebox. Finally, the samples were dried in a vacuum oven at room temperature for 24 h. The material was activated by heating up to 393 K during 1 h and kept at this temperature for 12 h under vacuum prior to CO_2_ adsorption isotherm measurements and TSA cycles.

### Synthesis of Cr‐BDC Amine Impregnated in Methanol

The synthesis of these materials was done as a control to compare the effect of the epoxide crosslinking with the amines inside the MOF pores. The reaction conditions were the same as per the optimized amine‐epoxide composites, but in this case, the epoxide was not included during the synthesis. In short, 55 mg of Cr‐BDC was dispersed in 2 mL MeOH and the desired amount of amine (405 µL TAEA for the Cr‐BDC‐TAEA or 525 µL TEPA for the Cr‐BDC‐TEPA), which was also dissolved in 2 mL MeOH, was added to this suspension. The suspension was stirred at 298 K for 3 h. Finally, the mixture is centrifuged for 5 min at 7800 rpm and the supernatant is discarded. The resulting solid is vacuum dried at room temperature for 24 h. The material was activated by heating up to 393 K during 1 h and kept at this temperature for 12 h under vacuum prior to CO_2_ adsorption isotherm measurements and TSA cycles.

## Conflict of Interest

Gaznat SA has filed a European patent protecting the results disclosed in this article (EP patent application No. 23219539.6).

## Supporting information



Supporting Information

## Data Availability

The authors have already uploaded the data to zenodo, but have not yet released the data yet due to the patenting process. The authors will delay the data release until the final manuscript is accepted.
