# Supplementary material for: A Ship‐in‐a‐Bottle Strategy: Crosslinking Amines and Epoxides inside MOF Pores for Enhanced CO2 Capture Performance
Source: Adv Mater. 2025 Sep 1;37(44):e10138. doi: 10.1002/adma.202410138 (PMC12592911; doi:10.1002/adma.202410138)
Supplement: Supplementary file 1 — Supporting Information [file ADMA-37-e10138-s001.pdf]

ADVANCED MATERIALS

Supporting Information

for *Adv. Mater.*, DOI 10.1002/adma.202410138

A Ship-in-a-Bottle Strategy: Crosslinking Amines and Epoxides inside MOF Pores for Enhanced CO_2 Capture Performance

*Jordi Espín, Anita Justin, Alexandre Hueber, Anne Belin, Sanjay Venkatachalam, Himan Dev Singh, Emad Oveisi and Wendy L. Queen**

Supporting Information

A Ship-in-a-Bottle Strategy: Crosslinking Amines and Epoxides Inside MOF Pores for Enhanced CO_2 Capture Performance

*Jordi Espín,† Anita Justin,† Alexandre Hueber,†*Anne Belin,† Sanjay Venkatachalam,† *Himan Dev Singh, † Emad Oveisi,‡ Wendy L. Queen†**

† Institute of Chemical Sciences and Engineering, École Polytechnique Fédérale de Lausanne (EPFL), CH-1951 Sion, Switzerland

‡ Interdisciplinary Center for Electron Microscopy, École Polytechnique Fédérale de Lausanne (EPFL), CH-1015 Lausanne, Switzerland.

Table of contents

Section S1: Description of characterization techniques S3
X-Ray Diffraction (XRD) S3
Nuclear Magnetic Resonance (NMR) S3
Gas Adsorption S3
High Resolution Electron Spray Ionization Mass Spectrometry (HR ESI-MS)..................... S4
Heat of Adsorption (HOA) calculation S4
Ideal Adsorption Solution Theory (IAST) selectivity calculation S5
Thermogravimetric Analysis (TGA) S5
TGA cycles... S6
Elemental Analysis.. S7
Scanning Electron Microscopy (SEM)... S7
Energy Dispersive X-rays Spectroscopy (EDXS) S7
Breakthrough measurements S8
Diffuse Reflectance Infrared Fourier-Transform Spectroscopy (DRIFTS) S8
Section S2: Cr-BDC characterization... S9
Section S3: Amine-epoxide crosslinking optimization.. S14
CO_2 adsorption isotherms... S19
Reproducibility... S24
Scalability of the method... S28
PXRD.. S30
Pore size distribution and BET surface areas... S31
Imaging... S34
Polymer content (TGA and EA) S40
NMR spectroscopy.. S45
ESI-MS.. S63
N_2 adsorption isotherms.. S71
CO_2 adsorption at different temperatures and Heat of Adsorption.................................S72
TSA cycles in humid environments.. S87
CO_2 isotherms of control, wet impregnation in Cr-BDC S90
TSA cycling in pure CO_2.. S93
Accelerated CO_2 aging S109
Q_{st} for composites with tuned polymer composition... S110
References.. S114

Section S1: Characterization Techniques

X-Ray Diffraction:

X-ray diffraction (XRD) patterns were performed on a Bruker D8 diffractometer with Cu Kα radiation (λ= 1.5418 Å) at 40 kV and 40 mA.

Nuclear Magnetic Resonance (NMR)

Solution NMR spectra were recorded in deuterated solvents on a BRUKER AVIII HD 400 spectrometer; data are given in ppm relative to 1% TMS solution in D_2O using the solvent signals as a secondary reference (^1H). NMR spectra were recorded at room temperature. Digestion of the MOF composites was carried out by adding a NaOD/D_2O mixture (60 µL NaOD 40% in D_2O in 500 µL D_2O) to 2-3 mg of composite and subsequently applying sonication for 30 min until full dissolution. The Bruker Topspin software package (version 3.2) was used for measuring and MestReNova NMR software (version 11.0.1) was used for processing of the spectra.

Gas Adsorption:

For surface area adsorption measurements (N_2 at 77 K) and CO_2 adsorption isotherms at 313 K and higher temperatures, 80 - 100 mg of bare MOF and composites was transferred to a pre-weighed glass adsorption cell. For N_2 adsorption at 313 K, at least 300 mg of sample were used given the low adsorption of this gas in the porous materials reported in this work.

The adsorption cells with the samples were then transferred to the activation station Belsorp vac II and placed in vacuum at 150 ºC (bare MIL-101(Cr)) or 120 ºC (MIL-101-amine-epoxide composites) for 12 h for complete activation. Evacuated tubes containing degassed samples were then transferred to a balance and weighed to determine the mass of sample. The tube was transferred to the analysis port of the instrument Belsorp max. Nitrogen gas adsorption isotherms

at 77 K was measured in a dewar containing liquid nitrogen, while CO_2 and N_2 measurements at 313 K and above were performed using a recirculating water bath connected to an isothermal bath.

High Resolution Electron Spray Ionization Mass Spectrometry (HR ESI-MS):

High resolution mass spectrometry (HRMS) analysis was performed using Q Exactive HF Hybrid Quadrupole-Orbitrap mass spectrometer (Thermo Scientific, Germany) in ESI ionization mode with ionization source TriVersa NanoMate (Advion, USA) and quadrupole mass analyzer. Blank polymers were dissolved in methanol prior to the analysis, while 2-3 mg of composite samples were digested using a mixture of 100 µL NaOH 10M in 500 µL methanol and sonicated for 30 min.

Heat of Adsorption (HOA) calculation:

Procedure to determinate the isosteric enthalpies of adsorption:

CO_2 adsorption isotherms were collected at 313, 333 and 353 K and fitted to a dual-site Langmuir model following Equation 1.

$$q = q_{sat,1}\frac{b_1 P}{1+b_1 P} + q_{sat,2}\frac{b_2 P}{1+b_2 P}$$

where q is the adsorbed amount in mmol/g, $q_{sat,1}$ is the adsorption capacity for site 1, b_1 is the Langmuir parameter for site 1 ($q_{sat,2}$ and b_2 are the equivalent for site 2) and P is the pressure in Pa.

Next, the Clausius-Clapeyron equation (Equation 2) was subsequently used to calculate the isosteric enthalpy of adsorption, Q_{st}, for CO_2.

$$Ln\,P = -\frac{Q_{st}}{R}\left(\frac{1}{T}\right) + C$$

Ideal Adsorbed Solution Theory (IAST) selectivity calculation:

Equation 1 was used to calculate the selectivity (α):

$$(1) \qquad \alpha = \frac{q_{CO_2|P_{CO_2}}}{q_{N_2|P_{N_2}}} \frac{P_{N_2}}{P_{CO_2}}$$

where $q_{CO_2|P_{CO_2}}$ is the is the adsorption amount of CO_2 under the corresponding CO_2 partial pressure, $q_{N_2|P_{N_2}}$ is the adsorption amount of N_2 under the corresponding N_2 partial pressure, and P_{CO_2} and P_{N_2} and are partial pressure of CO_2 and N_2. In order to better assess the selectivity, the values of $q_{CO_2|P_{CO_2}}$ and $q_{N_2|P_{N_2}}$ were obtained using a developed python package of Ideal Adsorption Solution Theory (IAST), which simulates the adsorption amount of an adsorbent material in multicomponent stream by using single component adsorption isotherms for each individual component.[1]

Thermogravimetric analysis (TGA):

For TGA runs from 20 to 800 ºC we used a Q300 instrument from TA under air flow (15 mL/min) and a 5 ºC/min temperature ramp. Approximately 5 mg of sample was employed in each test.

Equation 2 was used to calculate the organic content in the MOF composites:

Bare MOF residue at 800 ºC = 28.6 %

MOF composite residue at 800 ºC = 17.4 %

$$(2) \qquad Organic\ content = \left(1 - \frac{17.4}{28.6}\right) * 100 = 39\ \%$$

TGA cycles:

Dry tests:

A Q300 instrument from TA was used to perform the dry cycling experiments. Gas line 1 was connected to a N_2 cylinder (balance) and gas line 2 to a CO_2 cylinder (sample). The balance flow was set to 15 mL/min and the sample flow to 30 mL/min. A 60 min pretreatment at 120 ºC and under N_2 was applied prior to the start of all the cycles. The pure CO_2 gas stream was continuously flown while the temperature of the furnace was switched between 40 and 120 ºC, with isotherm times of 5 min in each step to mimic a TSA process. Approximately 10 mg of sample were used in each test.

Humid tests:

A TGA 55 from TA was used for the humid experiments. The setup includes 2 electrovalves controlled by the Event segment in the TA software Trios which open/closes the flow of the 15% CO_2 85% N_2 gas mixture cylinder used in the adsorption step and the 100% CO_2 gas cylinder used in the desorption step. The gas flow was controlled with 2 MFCs for each respective gas line from Alicat, which sent the selected gas to a water bubbler. The water bubbler was immersed in an oil bath at 40 ºC and the lines after the bubbler were heated to 40 ºC with a heating coil. The gas line 1 (balance) was connected to a 15% CO_2 85% N_2 gas mixture cylinder to avoid concentration dilution of the sample flow in the furnace. The reactive gas port (sample) was connected to the line downstream of the water bubbler. The temperature of the furnace was switched between 40 and 120 ºC, with isotherm times of 7 min in each step to mimic a TSA process. Approximately 10 mg of sample were used in each test.

Elemental Analysis (EA):

EA tests were carried out using a Unicube instrument from Elementar.

Equation 3 was used to calculate the organic content in the MOF composites:

$$(3) \qquad Organic\ content = \frac{CHN\ \%\ Composite - CHN\ \%\ Bare\ MOF}{CHN\ \%\ Bare\ MOF} * 100$$

Scanning Electron Microscopy (SEM):

The sample was deposited on an aluminum stub with a carbon tape. SEM analysis was performed on a Thermo Fisher Scientific Teneo at an accelerating voltage of 1.00 kV and using a beam current of 25 pA. SEM images were acquired with an in-column (Trinity) detector.

Energy Dispersive X-rays Spectroscopy (EDXS):

Energy dispersive X-ray spectroscopy (EDXS) in scanning transmission electron microscopy (STEM) was performed on a Thermo Fisher Scientific Titan-Themis 60-300 equipped with a high brightness Schottky X-FEG gun and Super-X EDXS system comprising four silicon drift detectors and Velox acquisition software. Samples for STEM-EDX analysis were prepared by embedding the crystals in an epoxy resin and serial sectioning in 40-80 nm thickness by cryo-ultramicrotomy using a Leica EM FC7 machine that was equipped with a Diatome cryo-knife (35° angle). Ultramicrotomy was carried out at −50 °C with a cutting speed of 0.2 mm s^{-1}, and ultramicrotomed sample slices were deposited on an ultrathin carbon support grid. EDXS data were collected in the form of spectrum images, in which a focused electron probe was scanned in a raster across a region of interest. For each scan point, structural information was obtained from the electron scattering incident on a high-angle annular dark-field (HAADF) detector, and simultaneously, an EDX spectrum was obtained by collecting X-rays emitted from the local volume probed by the electron

beam. Spectrum images were acquired with a probe current of approximately 0.3 nA, and a beam energy of 200 keV.

Breakthrough measurements:

Approximately 700 mg of each sample were activated at 120 ºC for 12 h under vacuum. For every test, 300 mg of activated sample and 300 mg of glass beads (108 um diameter) were mixed and used to fill the breakthrough bed inside the glovebox.

For dry experiments, dry He was used to purge the bed at 2 mL/min for 2 h at 313 K. After purging, the N_2:CO_2 mixture (85:15) was prepared for 20 min and then connected to the bed at the same flow and temperature. The outlet of the bed was connected to a mass spectrometer (Hiden HPR-20), which allowed for the simultaneous determination of the different gas molecules.

For the humid experiments, 300 mg of previously activated sample were mixed with 300 mg of glass beads and used to fill the bed. In this case, pre-saturation of the bed with water was done by flowing humid He (80 % RH) at 2 mL/min for 2 h at 313 K. After purging, the N_2:CO_2 mixture (85:15) with 80 % RH was prepared for 20 min and then connected to the bed at the same flow and temperature.

Diffuse Reflectance Infrared Fourier-Transform Spectroscopy (DRIFTS):

Diffuse Reflectance Infrared Fourier-Transform (DRIFT) spectra were recorded in the range of 400–4000 cm^{-1} using a PerkinElmer Frontier spectrometer equipped with a diamond ATR cell at a resolution of 4 cm^{-1}. Powder samples were pressed against the ATR crystal surface, and spectra were collected using air as the background.

MIL-101(Cr) $Cr_3(OH)(H_2O)_2O(BDC)_3$ (Cr-BDC) characterization

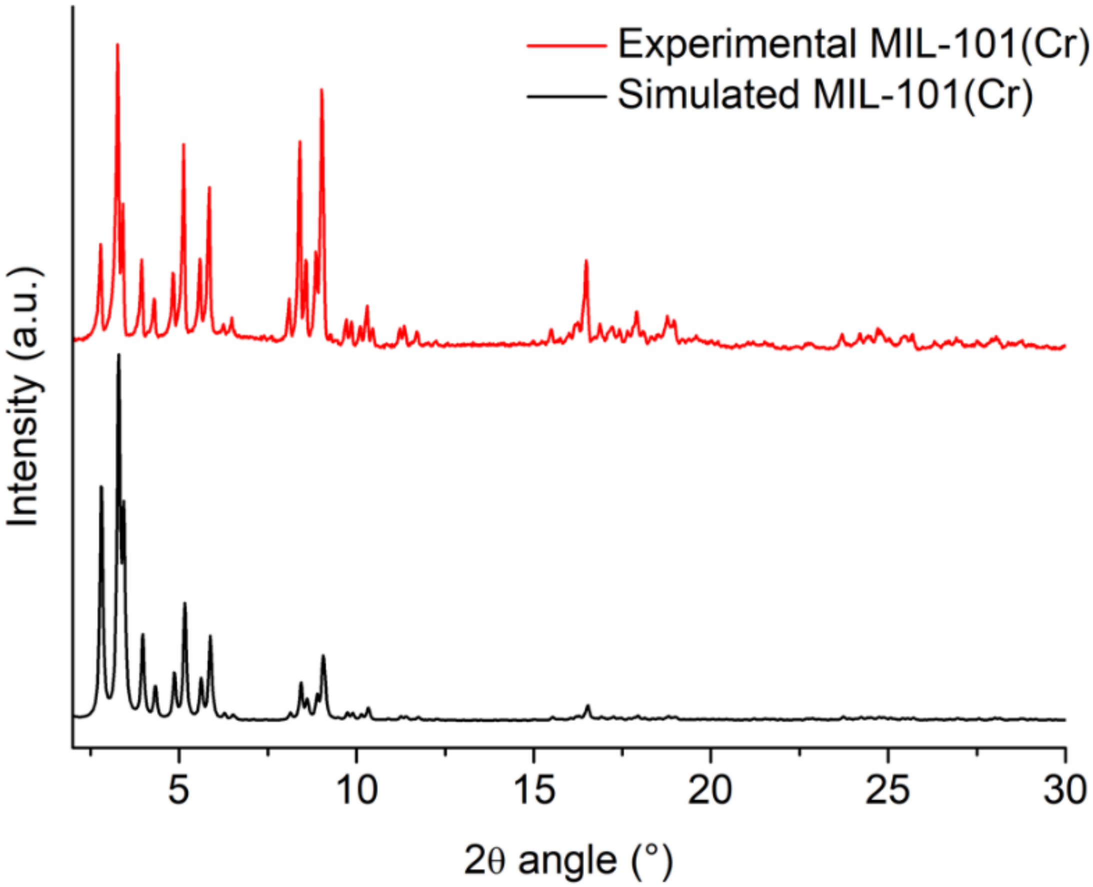

Figure S1. PXRD patterns of experimental and simulated Cr-BDC.

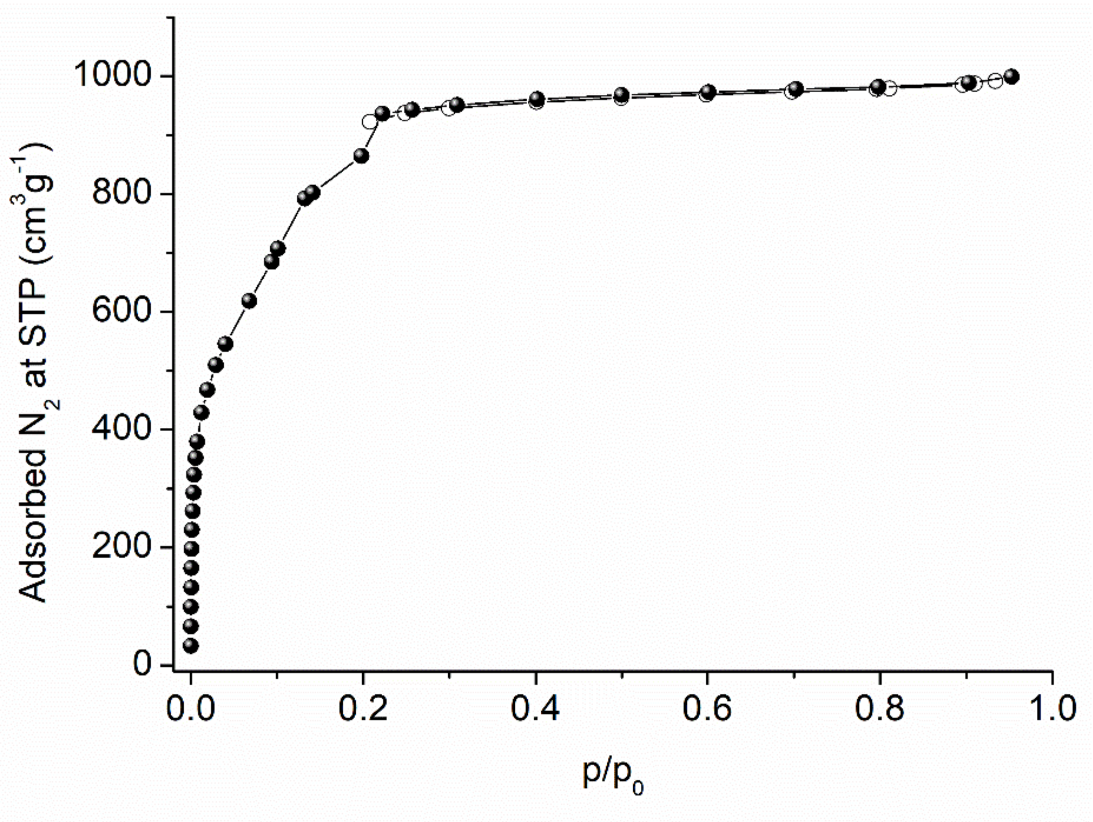

Figure S2. N_2 adsorption isotherm at 77 K of the synthesized Cr-BDC.

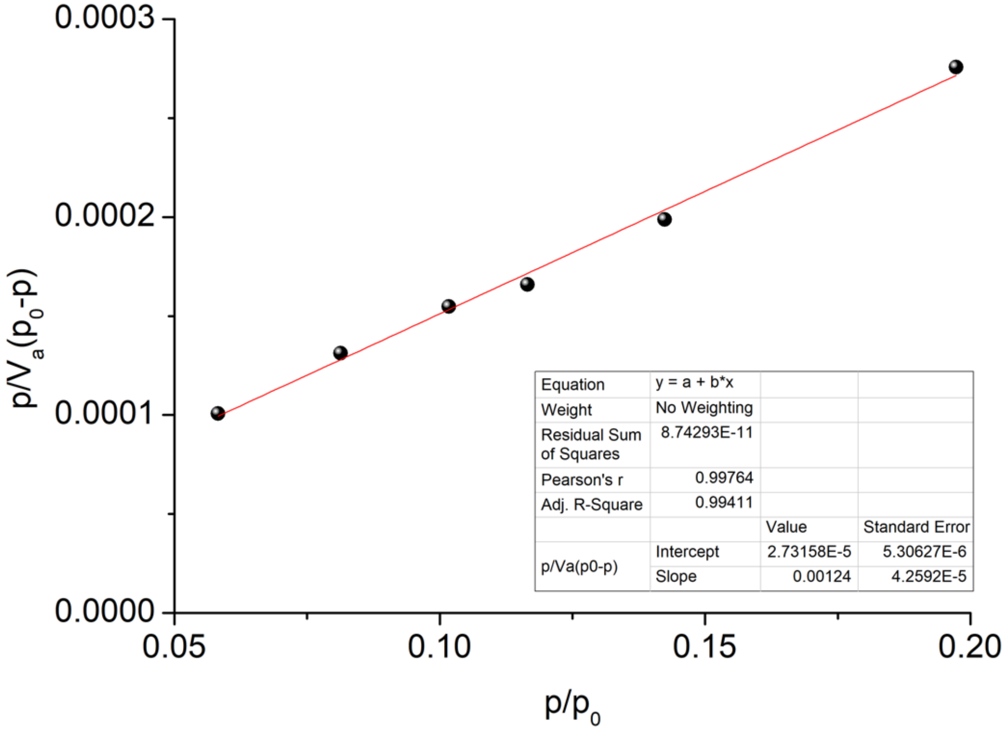

Figure S3. BET plot of Cr-BDC.

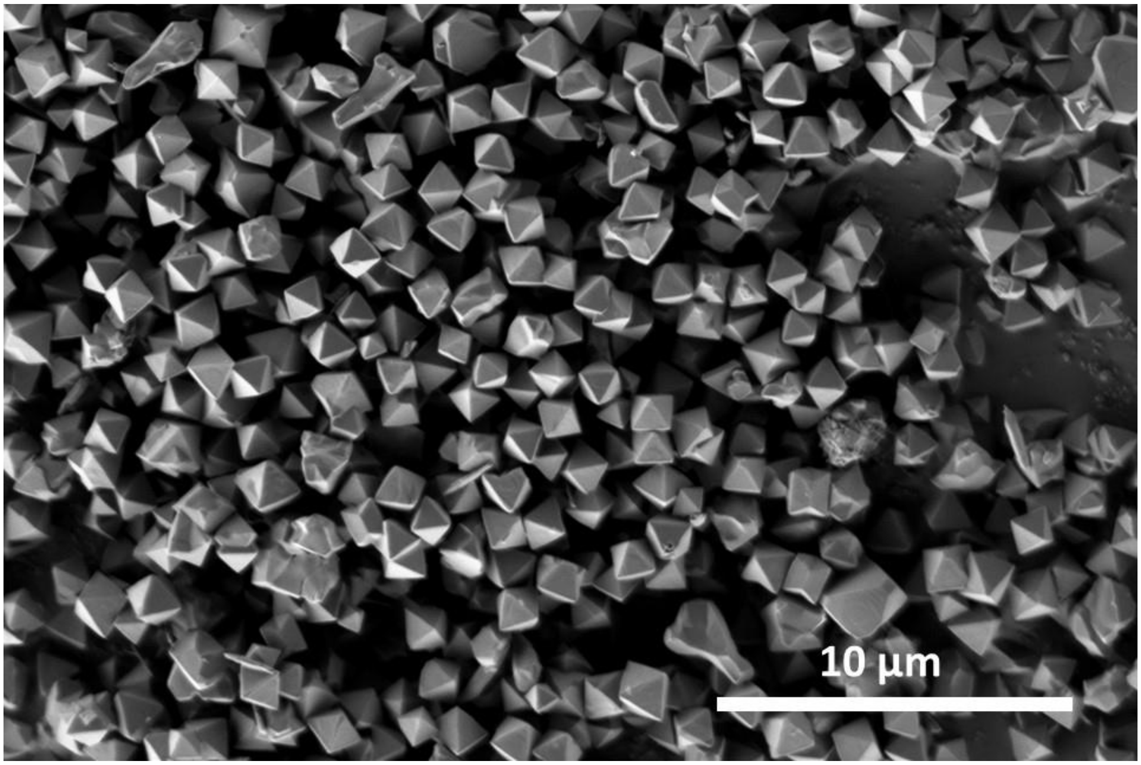

Figure S4. SEM image of the synthesized Cr-BDC.

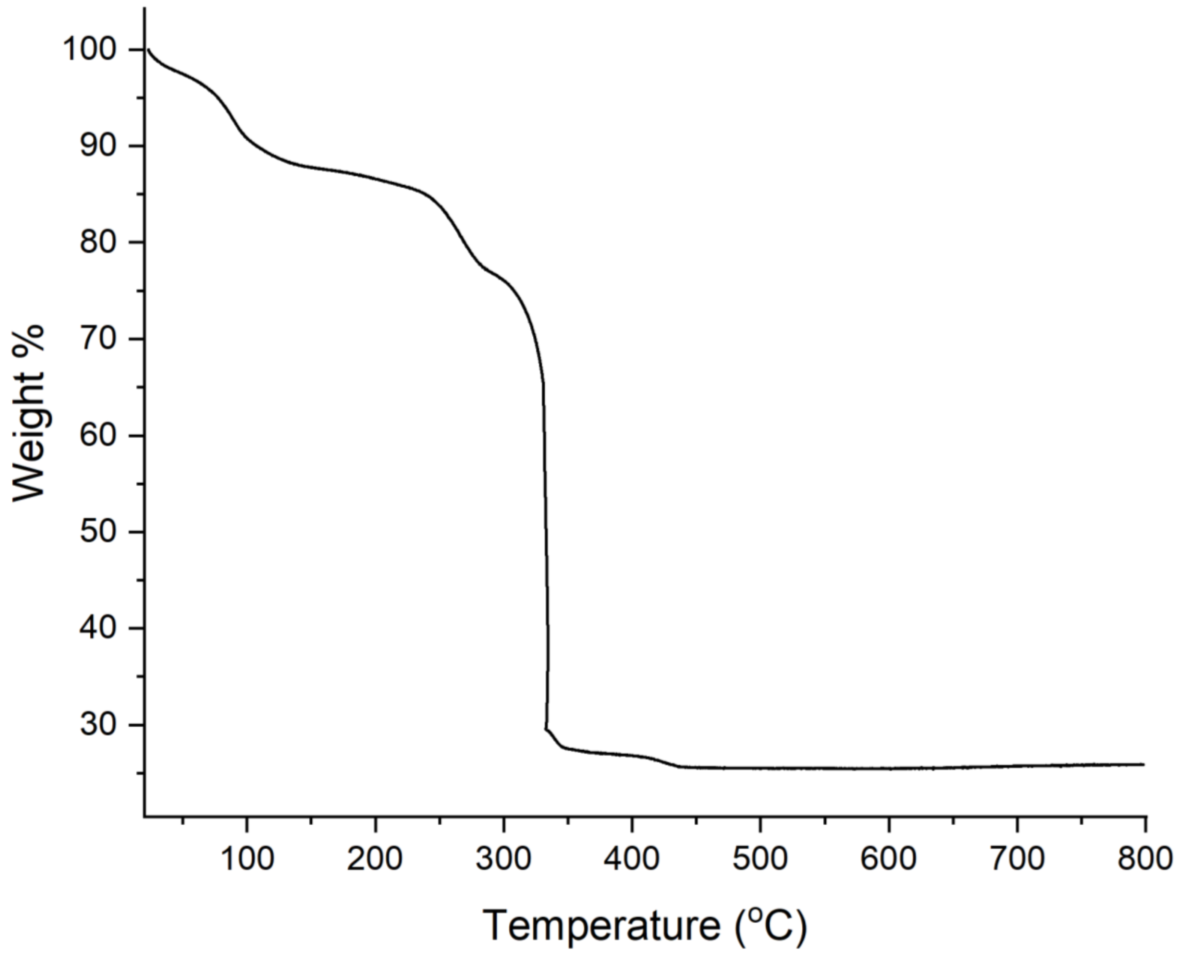

Figure S5. TGA plot of the synthesized Cr-BDC.

Amine-epoxide crosslinking optimization

Cr-BDC-TAEA-TMPTE

Table S1. Summary of the screening experiments tested for Cr-BDC-TAEA-TMPTE.

	Equivalents			Amount			Solvent	Time	Temperature	Wash	CO_2@313K, 0.15 bar
Entry	Cr-BDC	TAEA	TMPTE	MIL-101(Cr) (mg)	TAEA (uL)	TMPTE (uL)	MeOH (mL)	hours	(°C)		mmol/g
1	1	24 (2)	12 (1)	55	270	256	8	3	25	NO	1.02
2	1	48 (4)	12 (1)	55	540	256	8	3	25	NO	1.63
3	1	24 (1)	24 (1)	55	270	512	8	3	25	NO	0.53
4	1	24 (2)	12 (1)	55	270	256	4	3	25	NO	1.35
5	1	48 (4)	12 (1)	55	540	256	4	3	25	NO	2.15 (leach)
6	1	24 (1)	24 (1)	55	270	512	4	3	25	NO	0.54
7	1	24 (2)	12 (1)	55	270	256	2	3	25	NO	1.56
8	1	48 (4)	12 (1)	55	540	256	2	3	25	NO	Amine leach
9	1	24 (1)	24 (1)	55	270	512	2	3	25	NO	0.38
10	1	24 (1)	48 (2)	55	270	1024	4	3	25	NO	0.13
11	1	12 (1)	24 (2)	55	135	512	4	3	25	NO	0.23

12	1	12 (1)	48 (4)	55	135	1024	4	3	25	NO	0.07
13	1	48 (4)	12 (1)	55	540	256	4	3	50	NO	2.42 (leach)
14	1	48 (4)	12 (1)	55	540	256	4	12	25	NO	2.39 (leach)
15	1	48 (4)	12 (1)	55	540	256	4	12	50	NO	2.29 (leach)
16	1	36 (3)	12 (1)	55	405	256	4	3	25	NO	1.86
17	1	48 (2)	24 (1)	55	540	512	4	3	25	NO	1.46
18	1	48 (3)	16 (1)	55	540	341	4	3	25	NO	1.74
19	1	48 (4) rep1	12 (1)	55	540	256	4	3	25	NO	Amine leach
20	1	36 (3) rep1	12 (1)	55	405	256	4	3	25	NO	1.82
21	1	42 (3.5)	12 (1)	55	473	256	4	3	25	NO	2.08 (Slight leach)
22	1	48 (4)	12 (1)	55	540	256	4	3	50	NO + 2nd step 12 eq TMPTE	1.56
23	1	48 (4)	12 (1)	55	540	256	4	3	50	NO + 2nd step 12 eq BDE	1.07
24	1	36 (3)	12 (1)	55	405	256	4	3	50	NO	1.87
25	1	36 (3)	12 (1)	55	405	256	4	12	25	NO	2.10

26	1	36 (3)	12 (1)	55	405	256	4	12	50	NO	2.01
27	1	36 (3) repl	12 (1)	55	405	256	4	12	25	NO	1.8
28	1	36 (3)	12 (1)	55	405	256	4	3	25	YES after vac RT curing	1.43
29	1	36 (3)	12 (1)	55	405	256	4	3	25	NO + oven 50 ^{o}C curing	1.67
30	1	36 (3)	12 (1)	55	405	256	4	3	25	YES after oven 50 ^{o}C curing	1.50

Table S2. Summary of the screening experiments tested for Cr-BDC-TEPA-BDE.

	Equivalents			Amount			Solvent	Time	Temperature	Wash	CO_2@313K, 0.15 bar
Entry	Cr-BDC	TEPA	BDE	MIL-101(Cr) (mg)	TEPA (uL)	BDE (uL)	MeOH (mL)	hours	(°C)		mmol/g
1	1	24 (2)	12 (1)	55	350	71	4	3	25	NO	1.31
2	1	36 (2)	18 (1)	55	525	107	4	3	25	NO	0.91
3	1	36 (3)	12 (1)	55	525	71	4	3	25	NO	1.58
4	1	48 (4)	12 (1)	55	700	71	4	3	25	NO	Amine leach

Table S3. Summary of the screening experiments tested for Cr-BDC-TAEA-BDE.

	Equivalents			Amount			Solvent	Time	Temperature	Wash	CO_2@313K, 0.15 bar
Entry	Cr-BDC	TAEA	BDE	MIL-101(Cr) (mg)	TAEA (uL)	BDE (uL)	MeOH (mL)	hours	(°C)		mmol/g
1	1	24 (2)	36 (3)	55	270	213	4	3	25	NO	0.91
2	1	36 (2)	18 (1)	55	405	107	4	3	25	NO	1.83
3	1	24 (2)	12 (1)	55	270	71	4	3	25	NO	1.08
4	1	36 (3)	12 (1)	55	405	71	4	3	25	NO	2.81 slight leach

Table S4. Summary of the screening experiments tested for Cr-BDC-TEPA-TMPTE.

	Equivalents			Amount			Solvent	Time	Temperature	Wash	CO_2@313K, 0.15 bar
Entry	Cr-BDC	TEPA	TMPTE	MIL-101(Cr) (mg)	TEPA (uL)	TMPTE (uL)	MeOH (mL)	hours	(°C)		mmol/g
1	1	36 (3)	24 (2)	55	525	512	4	3	25	NO	1.53
2	1	36 (2)	18 (1)	55	525	384	4	3	25	NO	1.30
3	1	24 (2)	12 (1)	55	350	256	4	3	25	NO	1.55
4	1	36 (3)	12 (1)	55	525	256	4	3	25	NO	1.85

CO_2 adsorption isotherm triplicates at 313 K

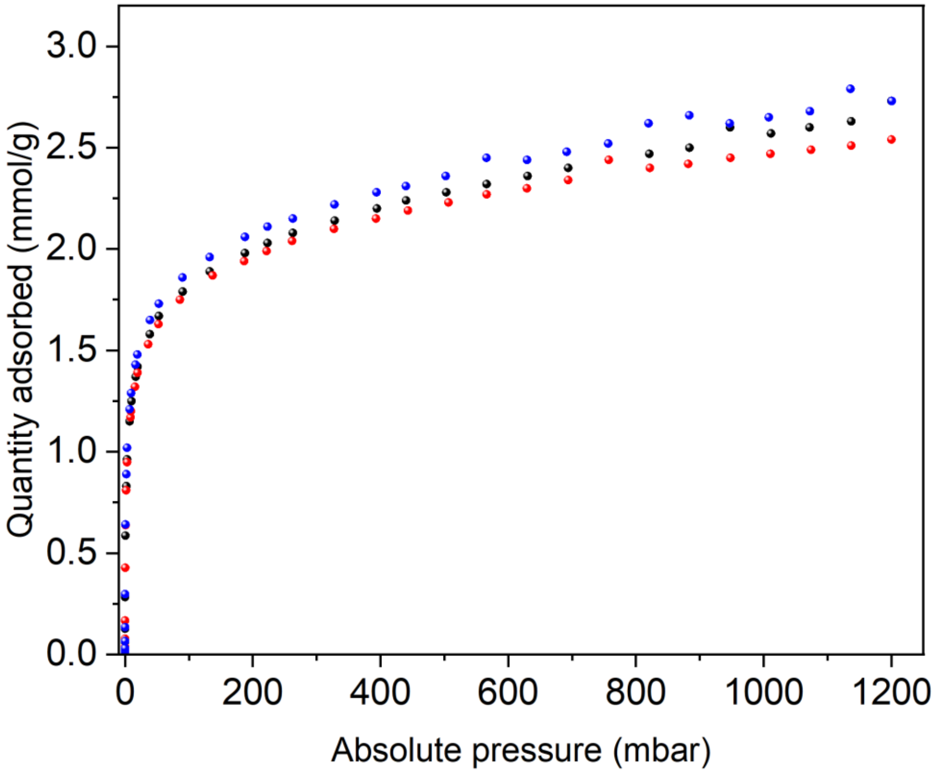

Figure S6. Triplicate Cr-BDC-TAEA-TMPTE (3:1) CO_2 adsorption isotherms at 313 K.

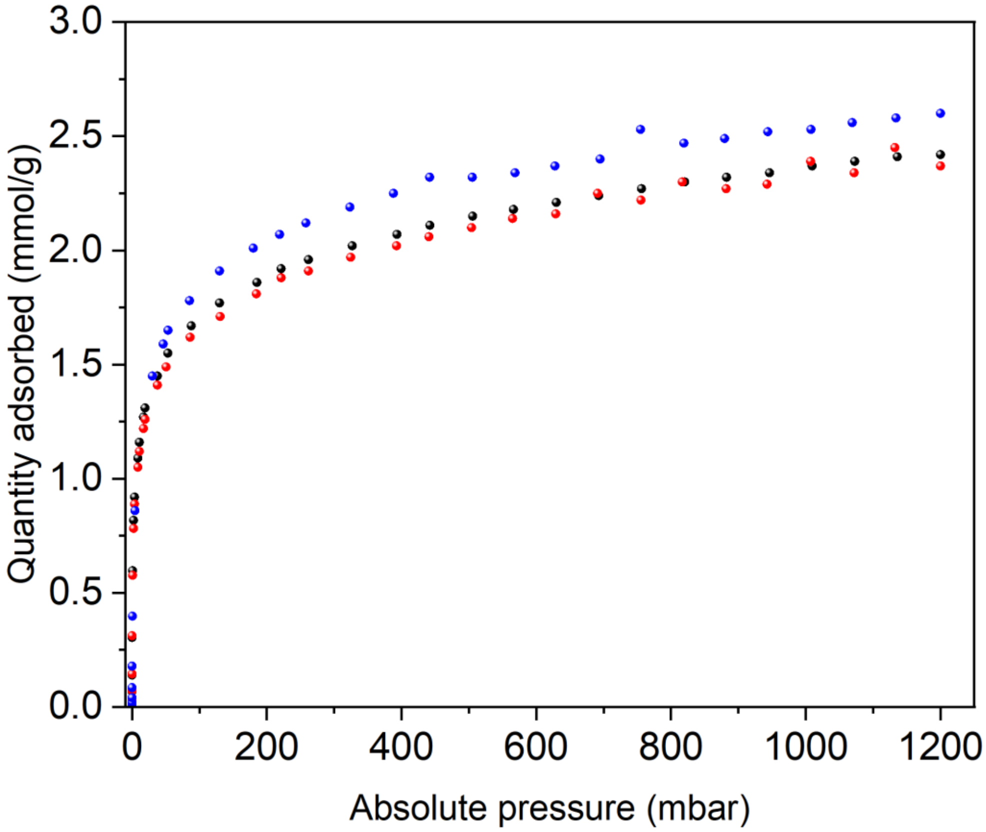

Figure S7. Triplicate Cr-BDC-TEPA-BDE (3:1) CO_2 adsorption isotherms at 313 K.

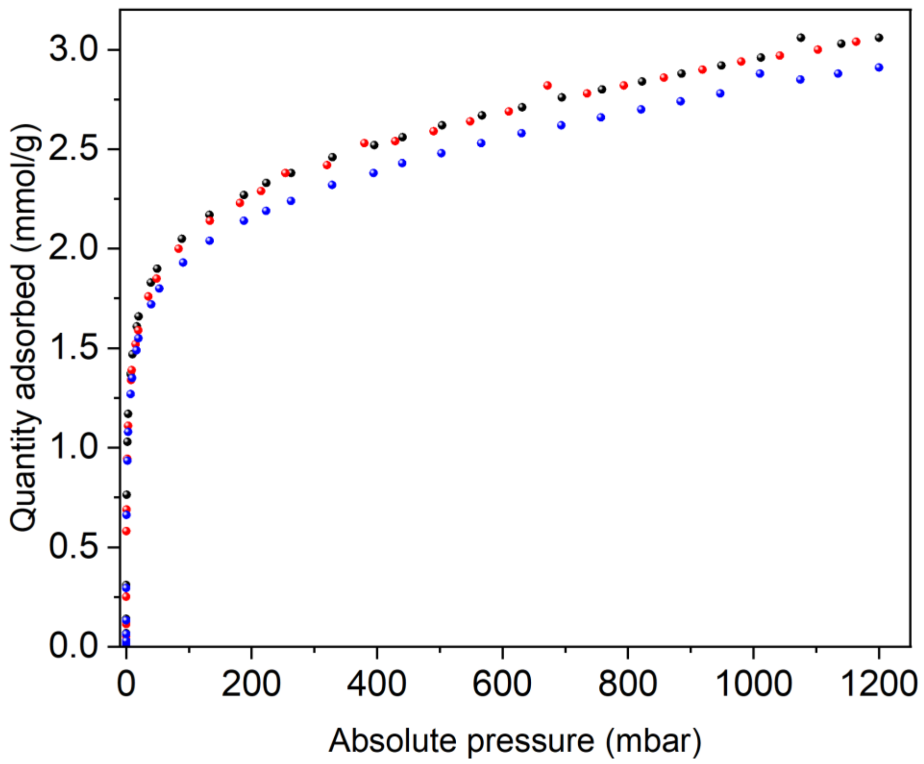

Figure S8. Triplicate Cr-BDC-TAEA-BDE (2:1) CO_2 adsorption isotherms at 313 K.

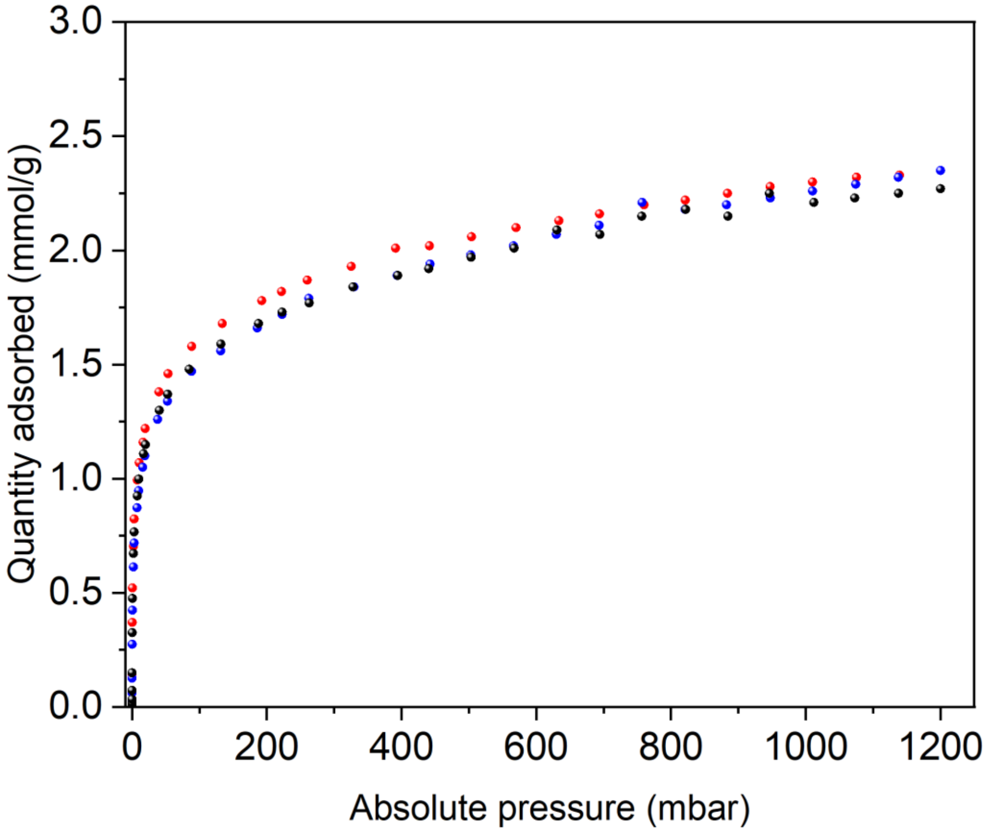

Figure S9. Triplicate Cr-BDC-TEPA-TMPTE (3:1) CO_2 adsorption isotherms at 313 K.

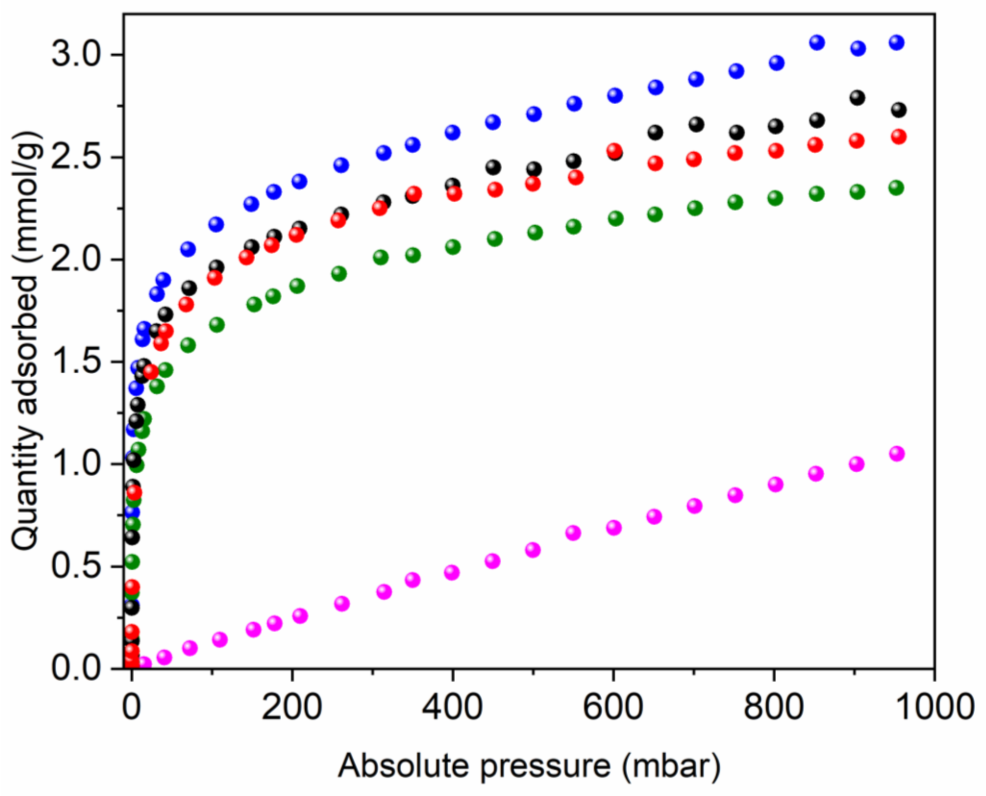

Figure S10. CO_2 adsorption isotherms at 313 K of the bare Cr-BDC (magenta) compared with the Cr-BDC-amine-epoxide composites; TEPA-TMPTE (black), TEPA-BDE (red), TAEA-BDE (blue) and TAEA-TMPTE (green). The synthesis of these materials was done at small scales (55 mg of starting Cr-BDC).

Table S5. Summary of the CO_2 adsorption capacities at 313 K for the 4 different composites synthesized by triplicate at the small scale (55 mg of starting Cr-BDC).

**Composite**	**CO_2 capacity at 313 K and 0.15 bar ± Standard deviation**
Cr-BDC-TAEA-TMPTE	2.00 ± 0.06 mmol/g
Cr-BDC-TEPA-BDE	1.90 ± 0.11 mmol/g
Cr-BDC-TAEA-BDE	2.22 ± 0.07 mmol/g
Cr-BDC-TEPA-TMPTE	1.71 ± 0.06 mmol/g

Reproducibility of the method using different Cr-BDC batches and synthesized by different collaborators

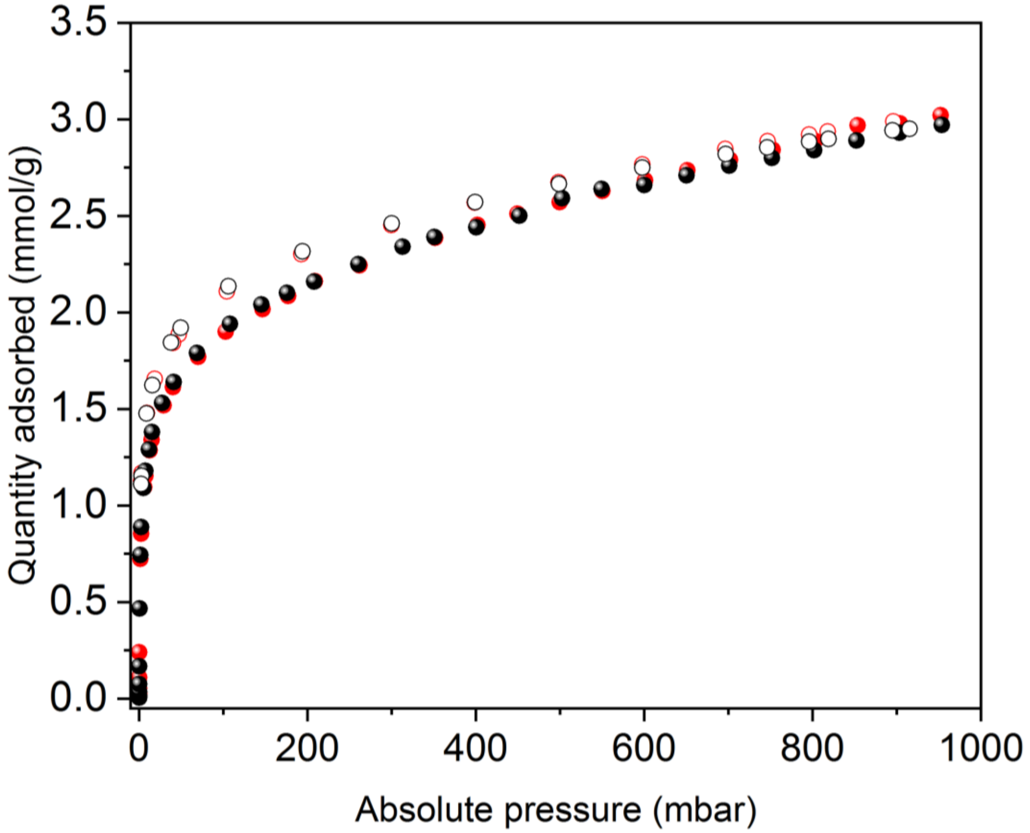

Figure S11. CO_2 adsorption isotherms at 313 K of Cr-BDC-TAEA-TMPTE synthesized by different people using different batches of Cr-BDC (550 mg of starting Cr-BDC).

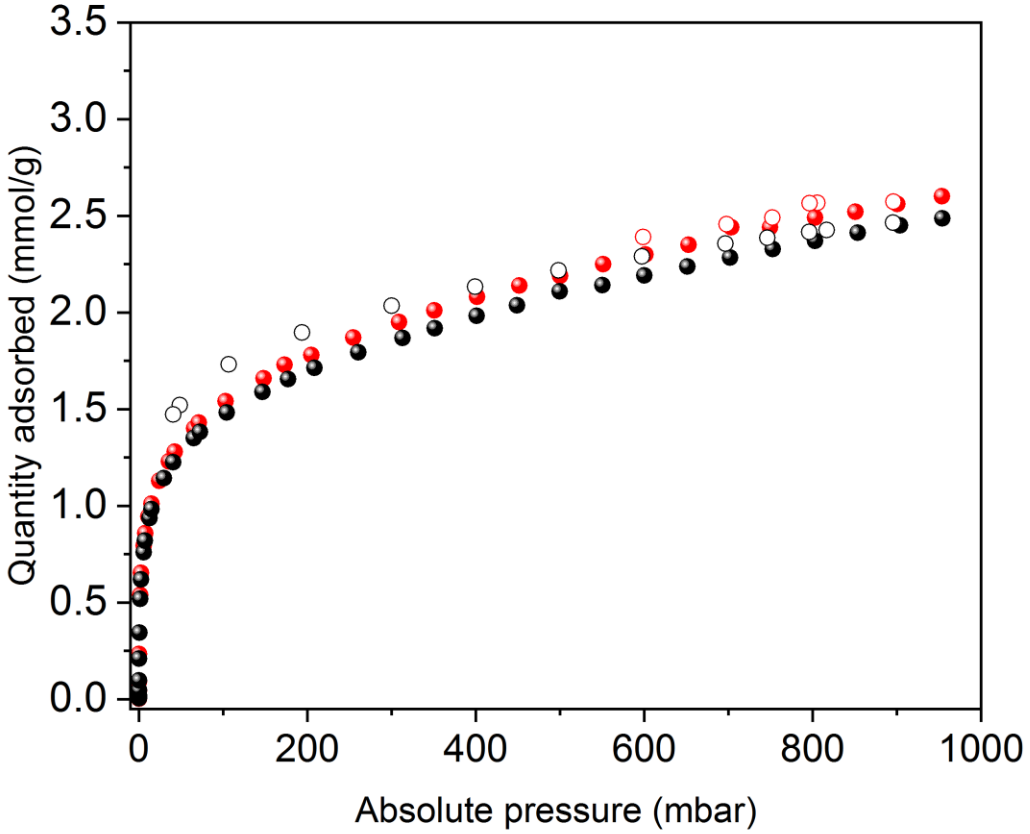

Figure S12. CO_2 adsorption isotherms at 313 K of Cr-BDC-TEPA-BDE synthesized by different people using different batches of Cr-BDC (550 mg of starting Cr-BDC).

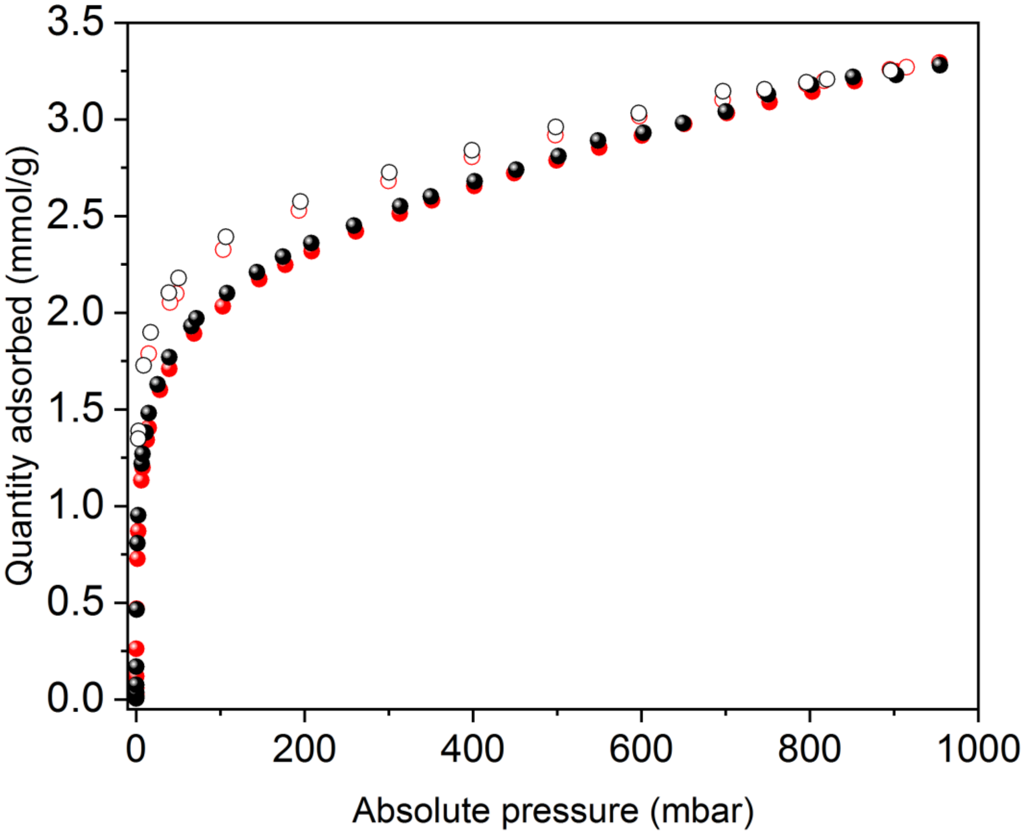

Figure S13. CO_2 adsorption isotherms at 313 K of Cr-BDC-TAEA-BDE synthesized by different people using different batches of Cr-BDC (550 mg of starting Cr-BDC).

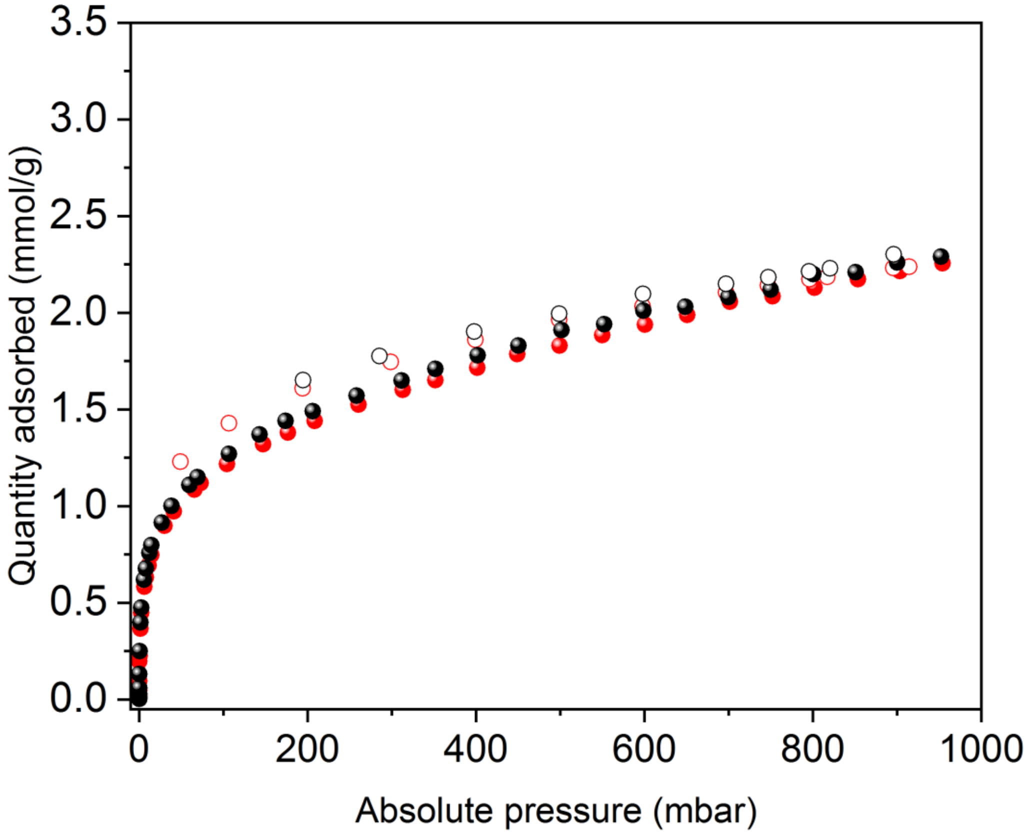

Figure S14. CO_2 adsorption isotherms at 313 K of Cr-BDC-TEPA-TMPTE synthesized by different people using different batches of Cr-BDC (550 mg of starting Cr-BDC).

Scalability of the method

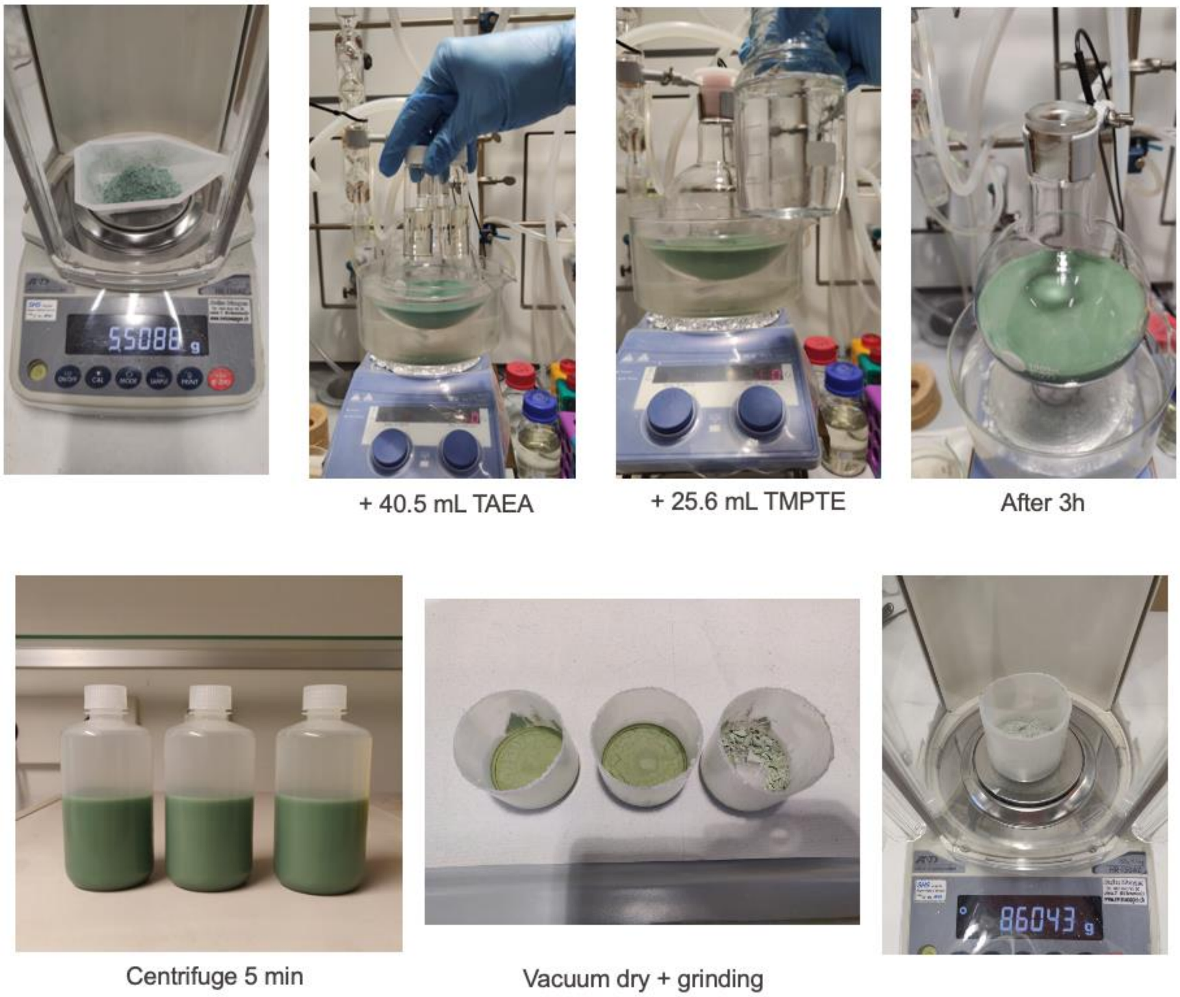

Figure S15. Pictures describing the 100-time scale-up process of Cr-BDC-TAEA-TMPTE. The scalability was proven by keeping the same MOF:solvent:reagents ratio as well as temperature and time (5.5 g of starting Cr-BDC).

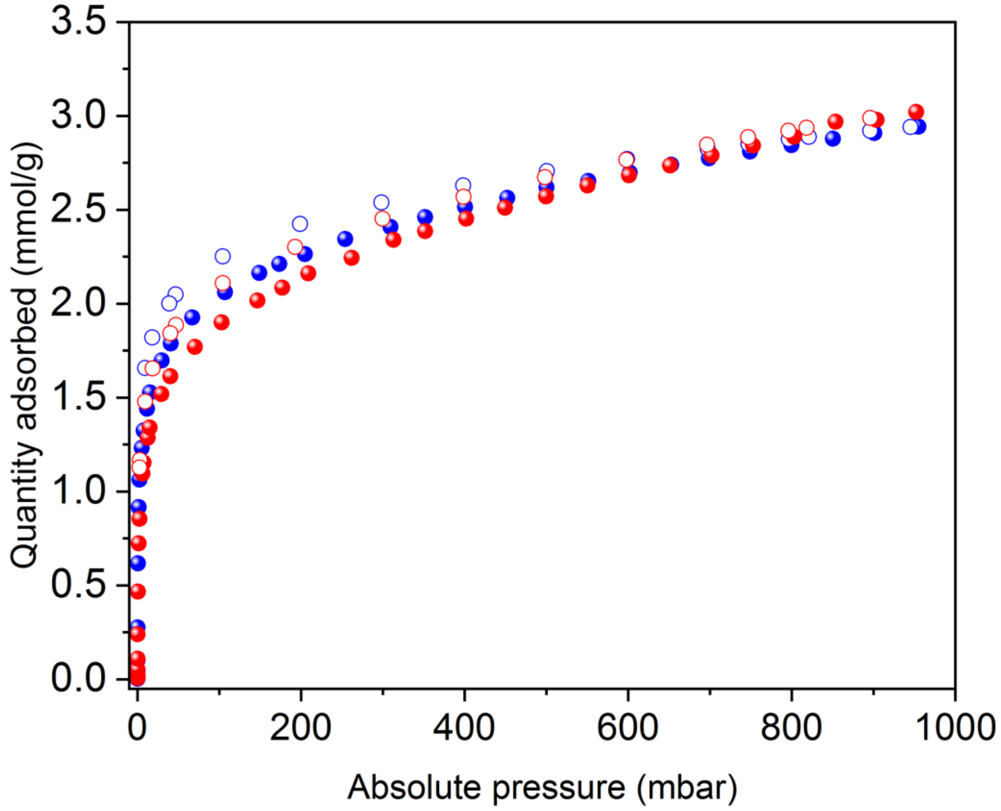

Figure S16. CO_2 adsorption isotherms at 313 K for the 10-times scale (red) and the 100-times scale (blue) Cr-BDC-TAEA-TMPTE composite.

PXRD

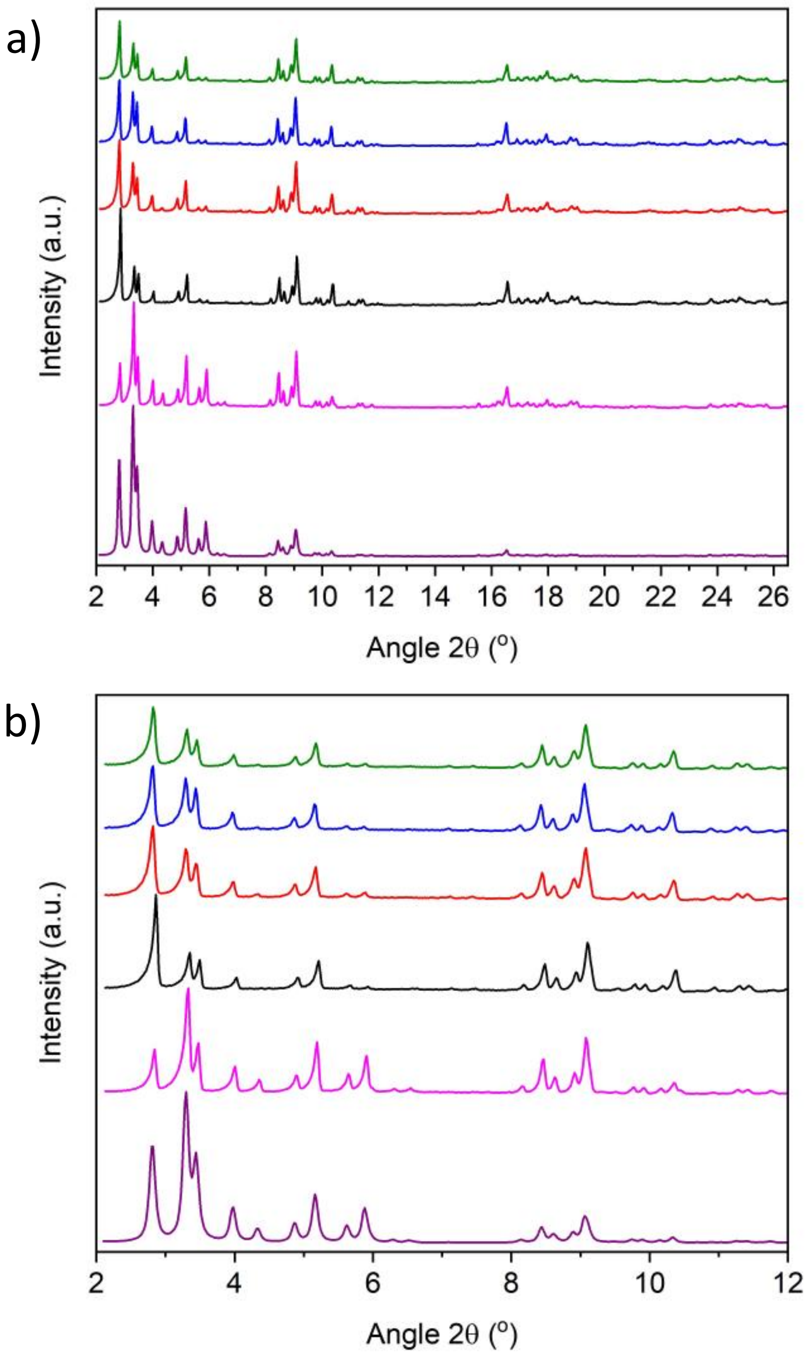

Figure S17. a) PXRD patterns of the simulated Cr-BDC (purple) compared with the bare Cr-BDC (magenta) and the amine-epoxide composites; Cr-BDC-TAEA-TMPTE (black), Cr-BDC-TEPA-BDE (red), Cr-BDC-TAEA-BDE (blue) and Cr-BDC-TEPA-TMPTE (green). b) Enlarged PXRD patterns between 2 and 12°.

BET measurements

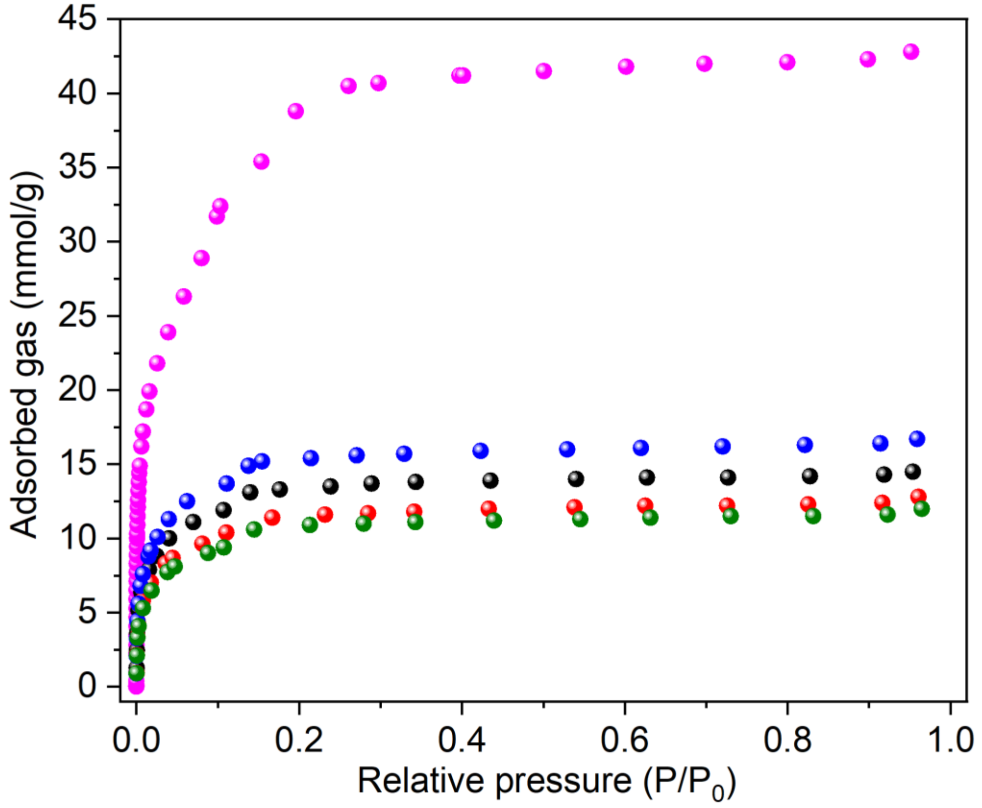

Figure S18. N_2 adsorption isotherms at 77 K of Cr-BDC (magenta) compared with the amine-epoxide modified composites Cr-BDC-TAEA-TMPTE (black), Cr-BDC-TEPA-BDE (red), Cr-BDC-TAEA-BDE (blue) and Cr-BDC-TEPA-TMPTE (green).

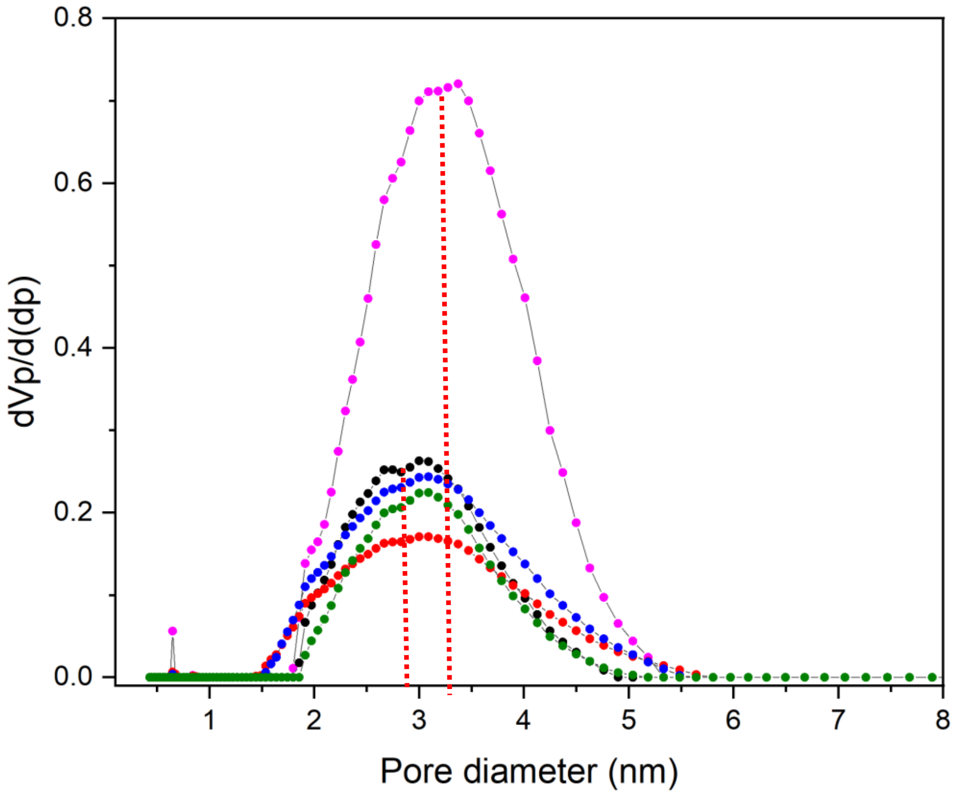

Figure S19. Pore size distribution plot for the obtained from the GCMC cage model using N_2 adsorption isotherms at 77 K of Cr-BDC (magenta) compared with the amine-epoxide modified composites Cr-BDC-TAEA-TMPTE (black), Cr-BDC-TEPA-BDE (red), Cr-BDC-TAEA-BDE (blue) and Cr-BDC-TEPA-TMPTE (green).

Table S6. Summary of the S_{BET}, V_p and pore size distribution for the bare Cr-BDC compared with the amine-epoxide modified composites.

**Sample**	**BET surface area (m^2/g)**	**Pore volume (cm^3/g)**	**Pore size distribution (nm)**
Cr-BDC	3309	1.65	3.34
Cr-BDC-TAEA-TMPTE	1126	0.50	3.03
Cr-BDC-TEPA-BDE	894	0.44	3.03
Cr-BDC-TAEA-BDE	1234	0.57	3.05
Cr-BDC-TEPA-TMPTE	866	0.41	3.05

SEM Images

Cr-BDC-TAEA-TMPTE

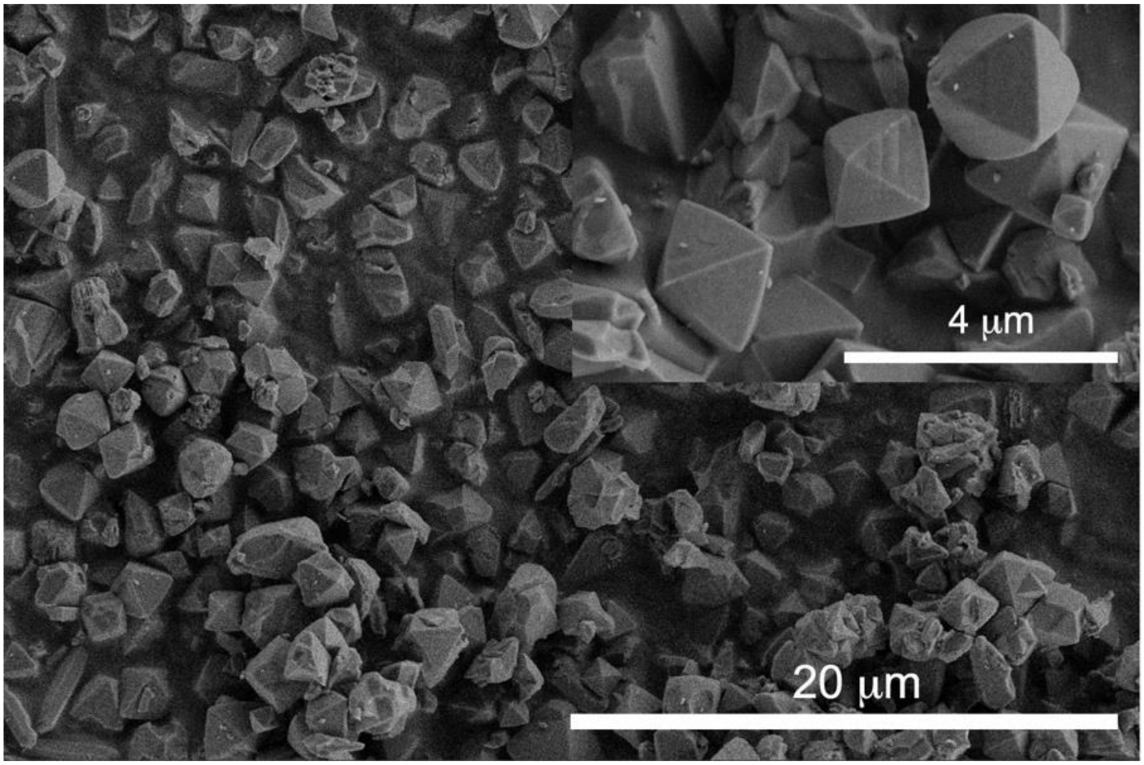

Figure S20. SEM image of Cr-BDC-TAEA-TMPTE.

Cr-BDC-TEPA-BDE

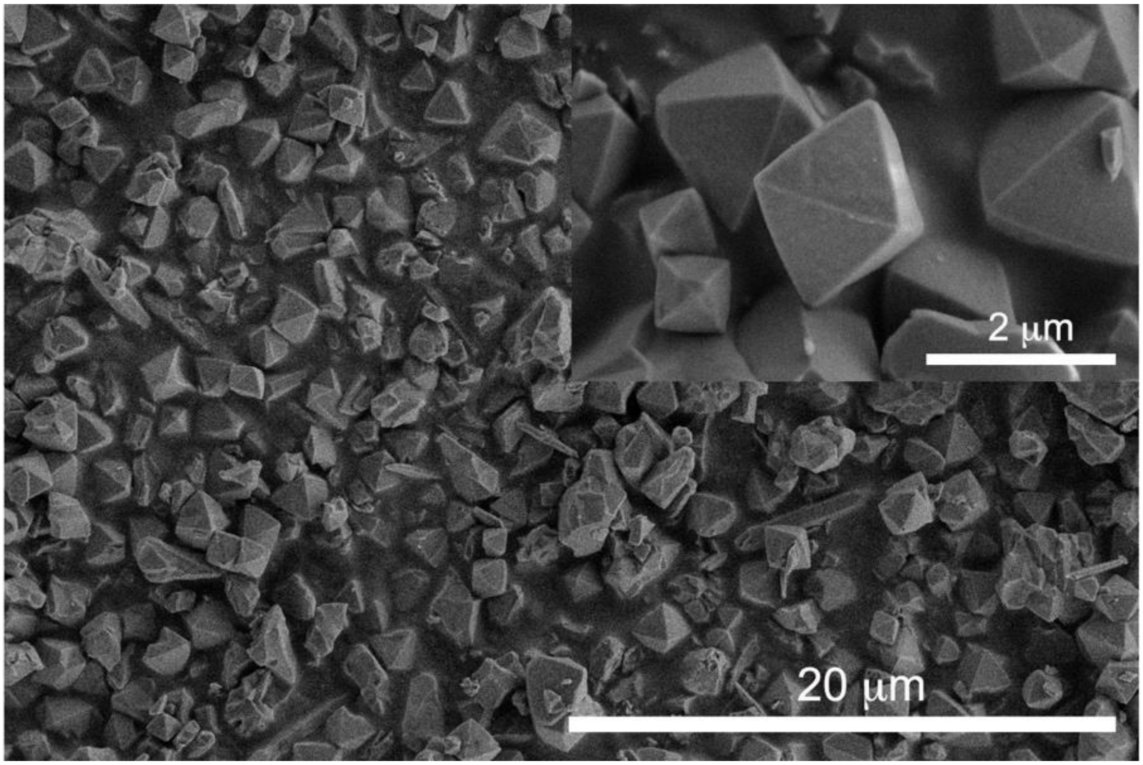

Figure S21. SEM image of Cr-BDC-TEPA-BDE.

Cr-BDC -TAEA-BDE

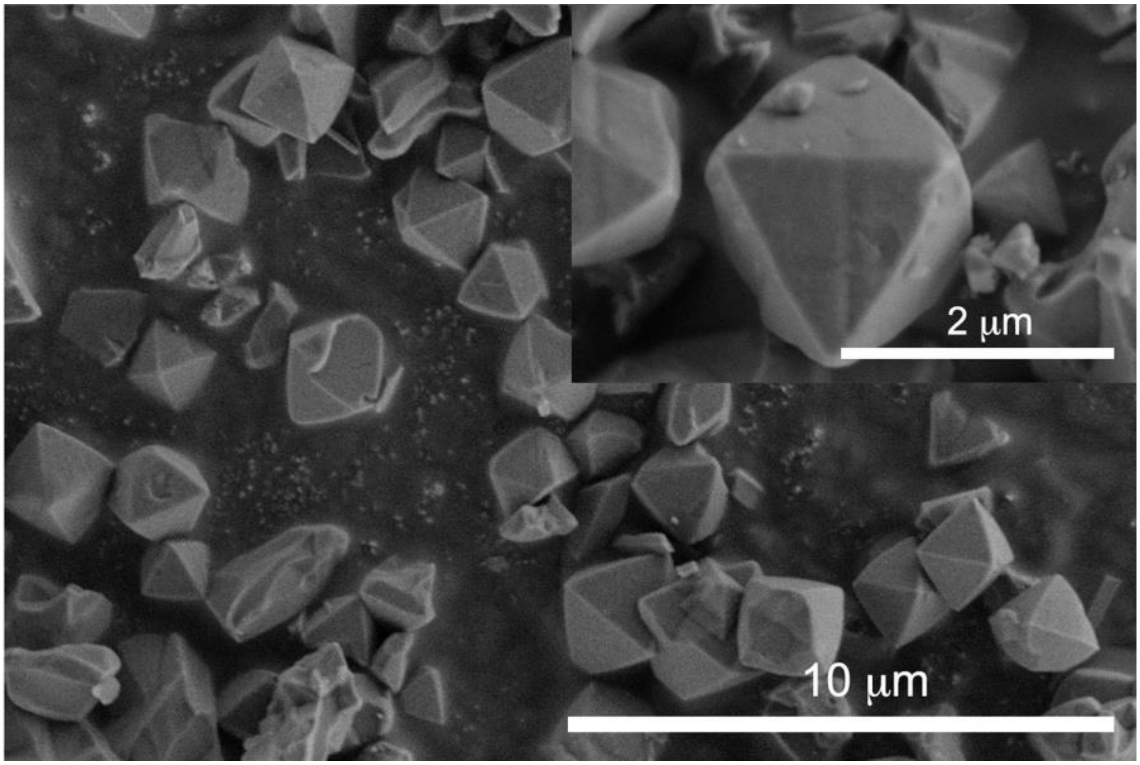

Figure S22. SEM image of Cr-BDC-TAEA-BDE.

Cr-BDC -TEPA-TMPTE

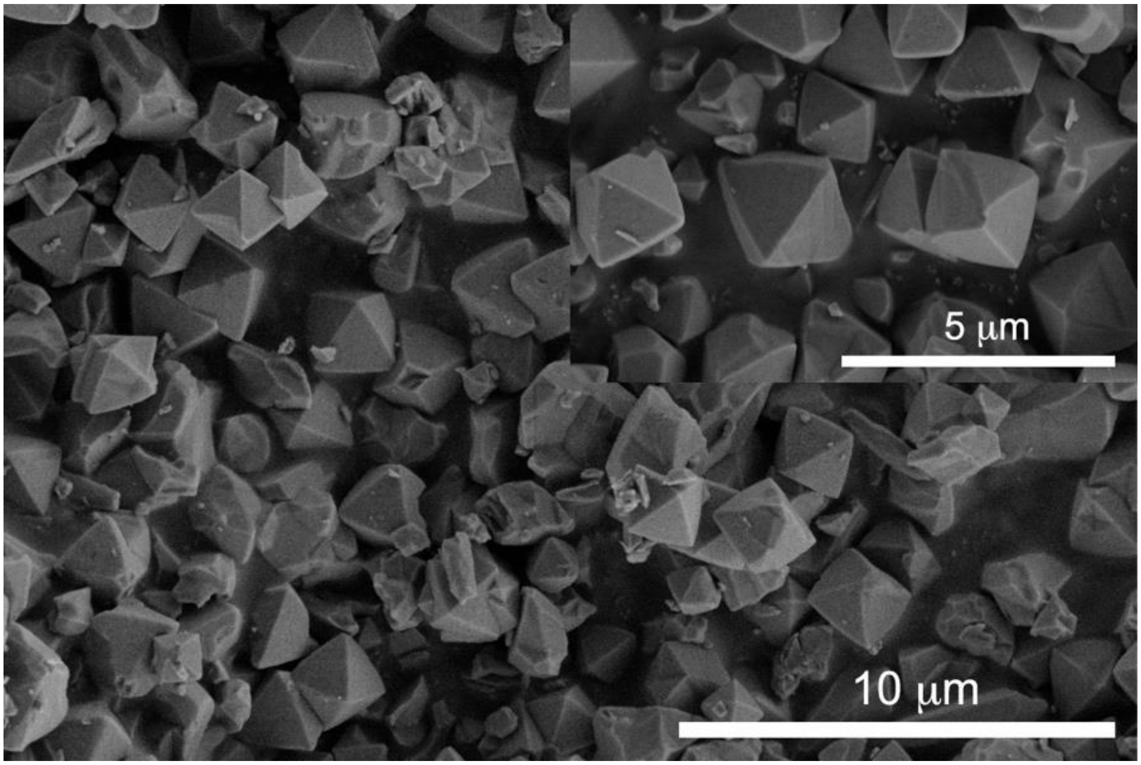

Figure S23. SEM image of Cr-BDC-TEPA-TMPTE.

EDX of sliced crystals

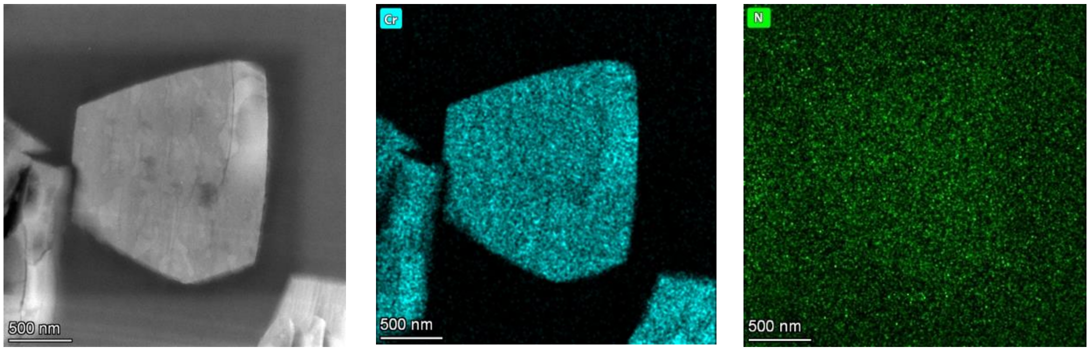

Figure S24. HAADF-STEM image of sliced crystals of bare Cr-BDC and corresponding EDXS elemental maps of Cr (turquoise) and N (green).

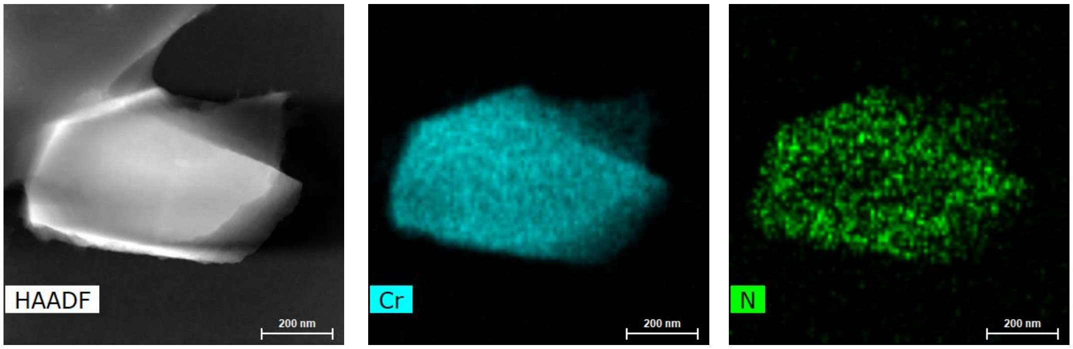

Figure S25. HAADF-STEM image of sliced crystals of bare Cr-BDC-TAEA-TMPTE and corresponding EDXS elemental maps of Cr (turquoise) and N (green).

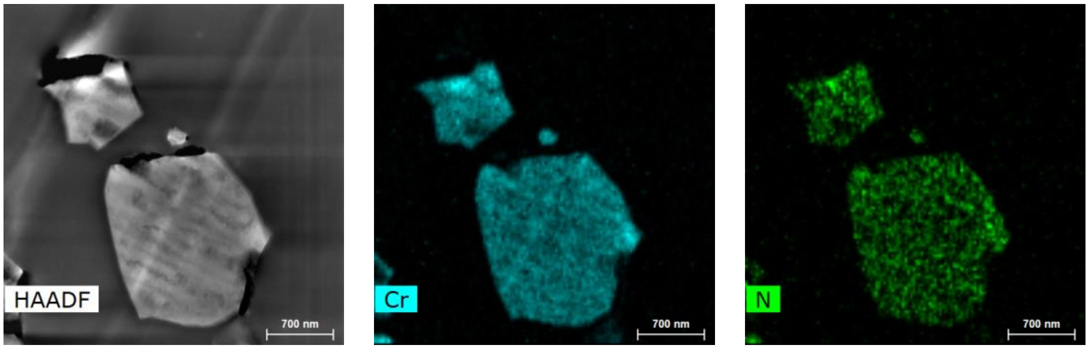

Figure S26. HAADF-STEM image of sliced crystals of bare Cr-BDC-TEPA-BDE and corresponding EDXS elemental maps of Cr (in turquoise) and N (in green).

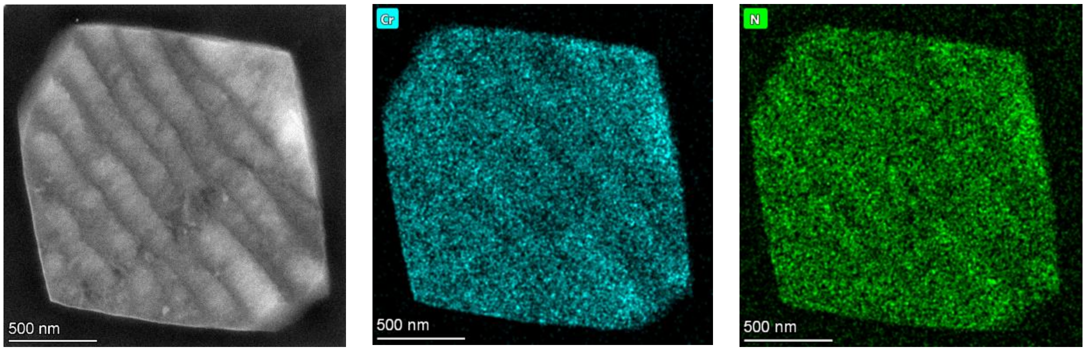

Figure S27. HAADF-STEM image of sliced crystals of bare Cr-BDC-TAEA-BDE and corresponding EDXS elemental maps of Cr (in turquoise) and N (in green).

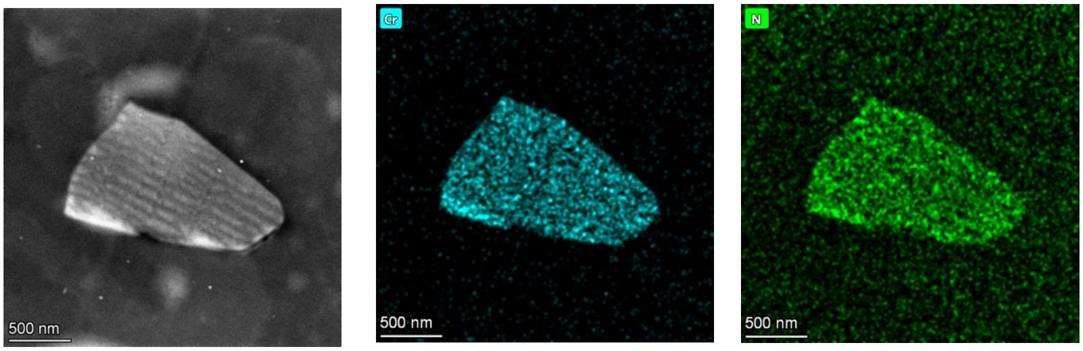

Figure S28. HAAFD-STEM image of sliced crystals of bare Cr-BDC-TEPA-TMPTE and corresponding EDXS elemental maps of Cr (in turquoise) and N (in green).

TGA

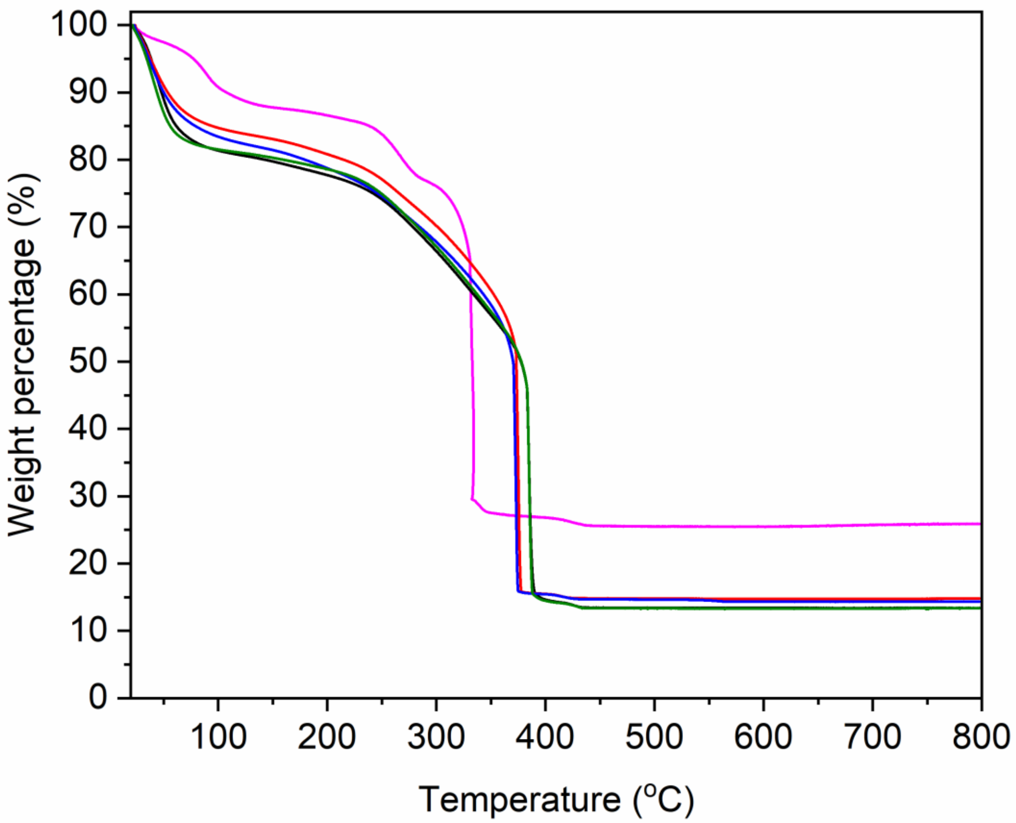

Figure S29. TGA plot from 20 to 800 °C of the bare Cr-BDC (magenta) compared with the activated amine-epoxide composites; Cr-BDC-TAEA-TMPTE (black), Cr-BDC-TEPA-BDE (red), Cr-BDC-TAEA-BDE (blue) and Cr-BDC-TEPA-TMPTE (green).

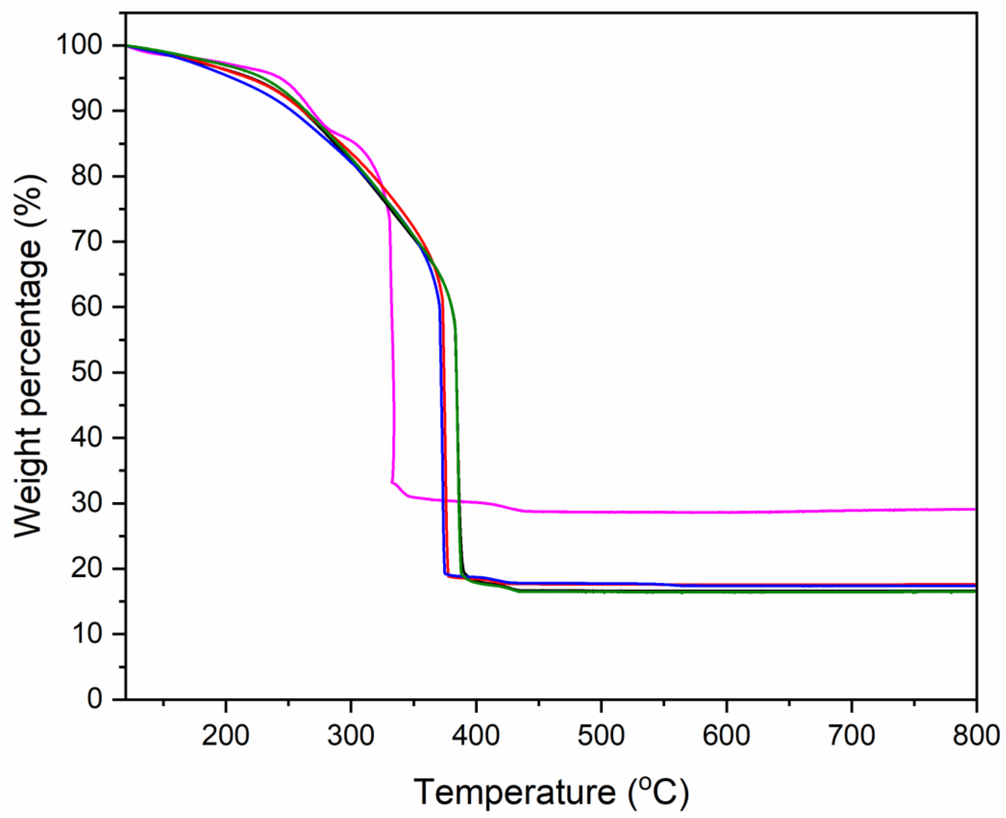

Figure S30. Normalized TGA plot at 120 °C of the bare Cr-BDC (magenta) compared with the activated amine-epoxide composites; Cr-BDC-TAEA-TMPTE (black), Cr-BDC-TEPA-BDE (red), Cr-BDC-TAEA-BDE (blue) and Cr-BDC-TEPA-TMPTE (green).

Table S7. Summary of the polymer content % calculated from TGA curves.

**Sample**	**Polymer content calculated from TGA (%)**
Cr-BDC-TAEA-TMPTE	39
Cr-BDC-TEPA-BDE	39
Cr-BDC-TAEA-BDE	42
Cr-BDC-TEPA-TMPTE	43

Elemental analysis

Table S8. Elemental analysis data including C, H, N, S % of the bare Cr-BDC and the amine-epoxide composites.

Sample	Element	%	%	%	Avg	stdev
**MIL-101(Cr)**	**C**	36.27	36.23	36.08	**36.19**	0.10
	**H**	3.61	3.526	3.573	**3.57**	0.04
	**N**	2.29	2.31	2.28	**2.29**	0.02
	**S**	0.081	0.04	0.039	**0.053**	0.02
**MIL-101(Cr)-TAEA-TMPTE**	**C**	47.05	46.79		**46.92**	0.18
	**H**	7.133	7.129		**7.13**	0.002
	**N**	10.29	10.41		**10.35**	0.08
	**S**	0	0		**0**	0
**MIL-101(Cr)-TEPA-BDE**	**C**	44.67	44.48		**44.57**	0.13
	**H**	6.757	7.197		**6.97**	0.31
	**N**	12.96	12.73		**12.84**	0.16
	**S**	0	0		**0**	0
**MIL-101(Cr)-TAEA-BDE**	**C**	44.54	43.23	43.39	**43.72**	0.71
	**H**	6.605	6.712	6.577	**6.63**	0.07
	**N**	12.06	11.71	11.97	**11.91**	0.18
	**S**	0	0	0	**0**	0
**MIL-101(Cr)-TEPA-TMPTE**	**C**	45.86	46.24	44.85	**45.65**	0.72
	**H**	7.165	6.891	6.856	**6.97**	0.17
	**N**	10.86	11.27	10.59	**10.90**	0.34
	**S**	0	0	0	**0**	0

Table S9. Summary of the polymer content % calculated from EA tests.

**Sample**	**Polymer content calculated from EA (%)**
Cr-BDC-TAEA-TMPTE	35
Cr-BDC-TEPA-BDE	35
Cr-BDC-TAEA-BDE	33
Cr-BDC-TEPA-TMPTE	34

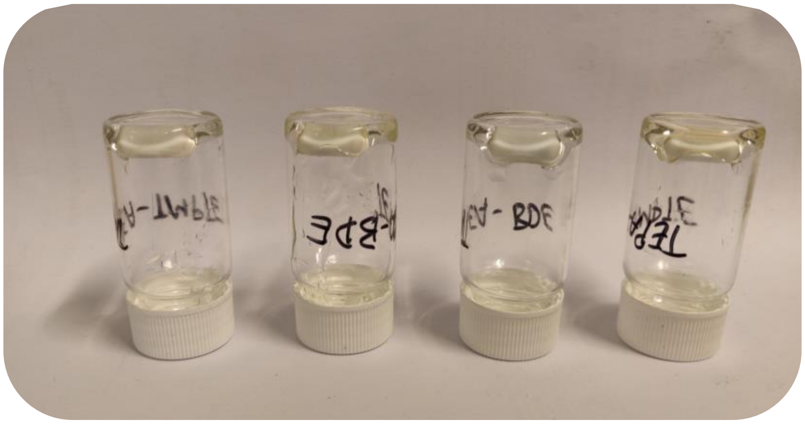

Figure S31. Bare amine-epoxide polymers synthesized reproducing the composite synthesis, but without introducing MOF. The honey-like texture obtained after vacuum drying at room temperature confirms some degree of crosslinking.

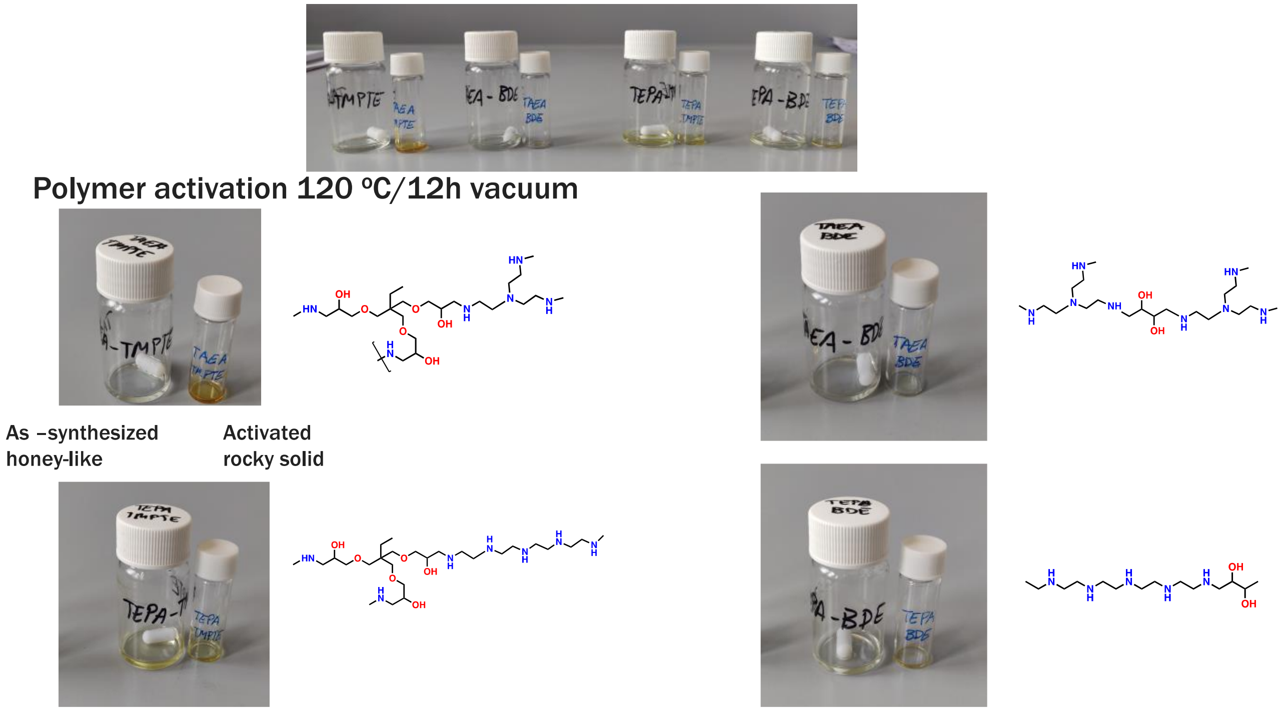

Figure S32. Texture of the bare amine-epoxide polymers before and after activation step at 120 ºC for 12 h under vacuum. A clear change from honey-like polymer to a rocky solid can be observed as effect of the activation step under such conditions, which favors further crosslinking between both monomers.

NMR characterization

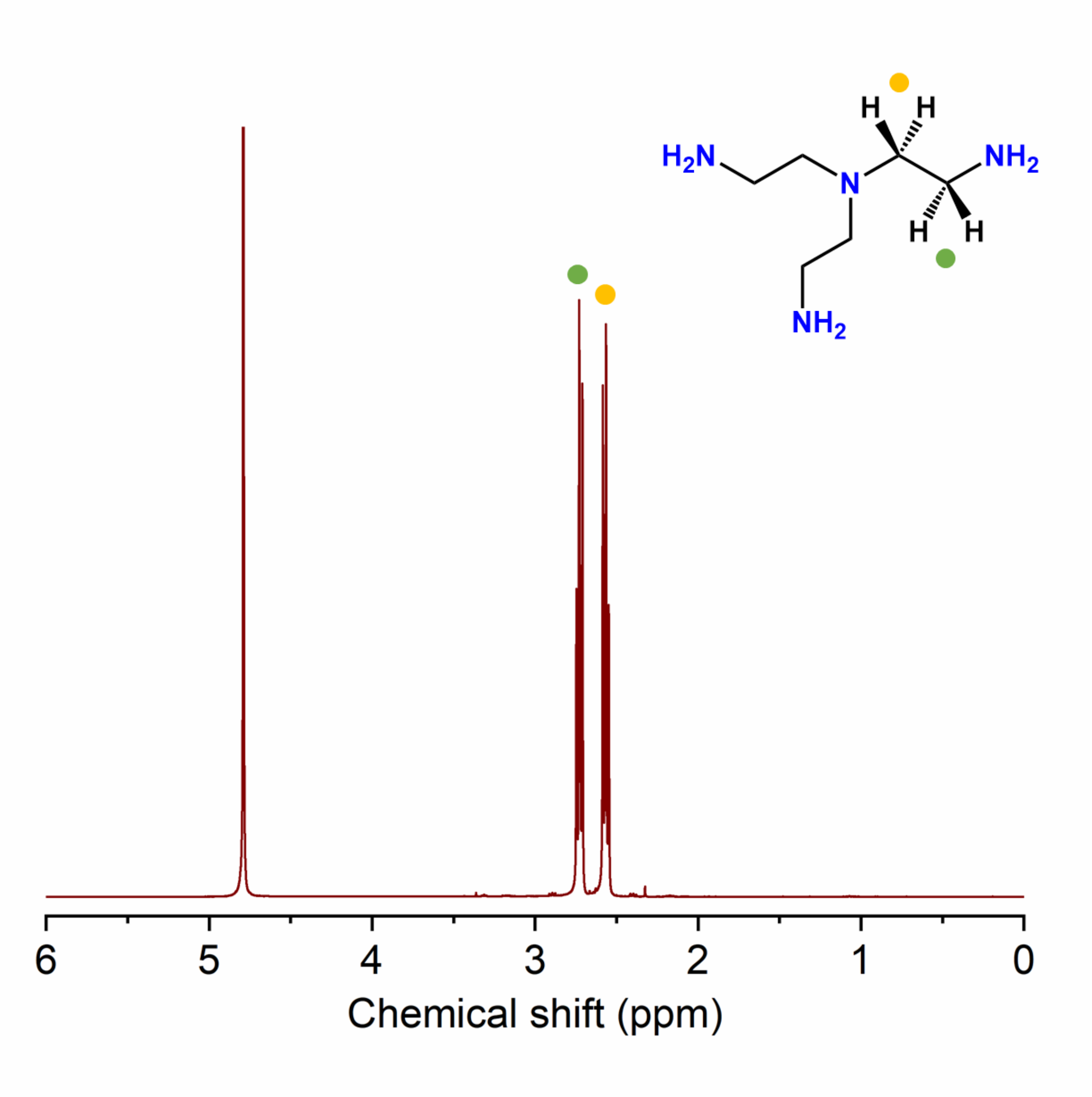

Figure S33. ^{1}H NMR spectrum of TAEA in D_2O. Non-symmetric protons are drawn, while the rest are removed for clarity.

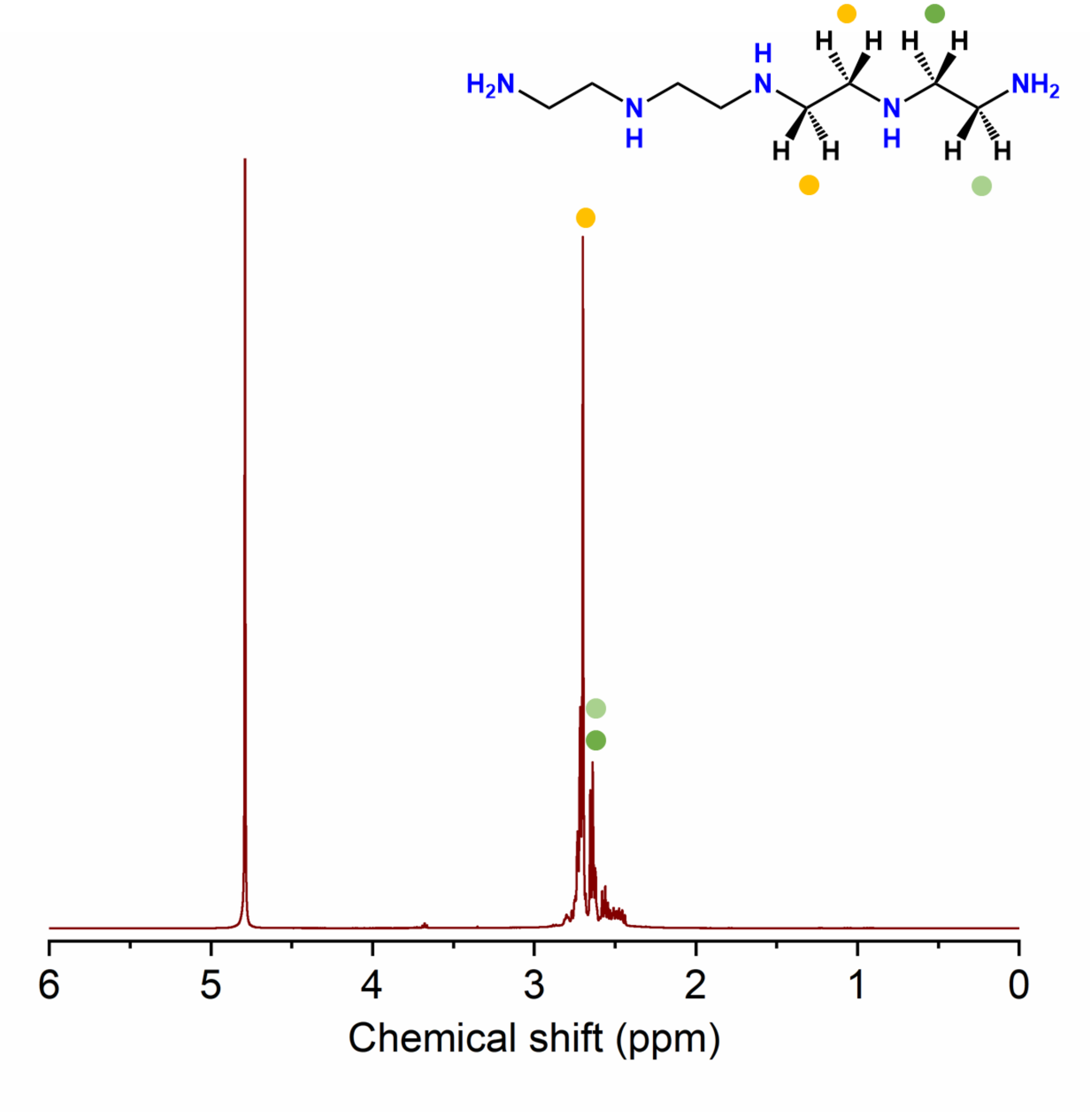

Figure S34. ^{1}H NMR spectrum of TEPA in D_2O. Non-symmetric protons are drawn, while the rest are removed for clarity.

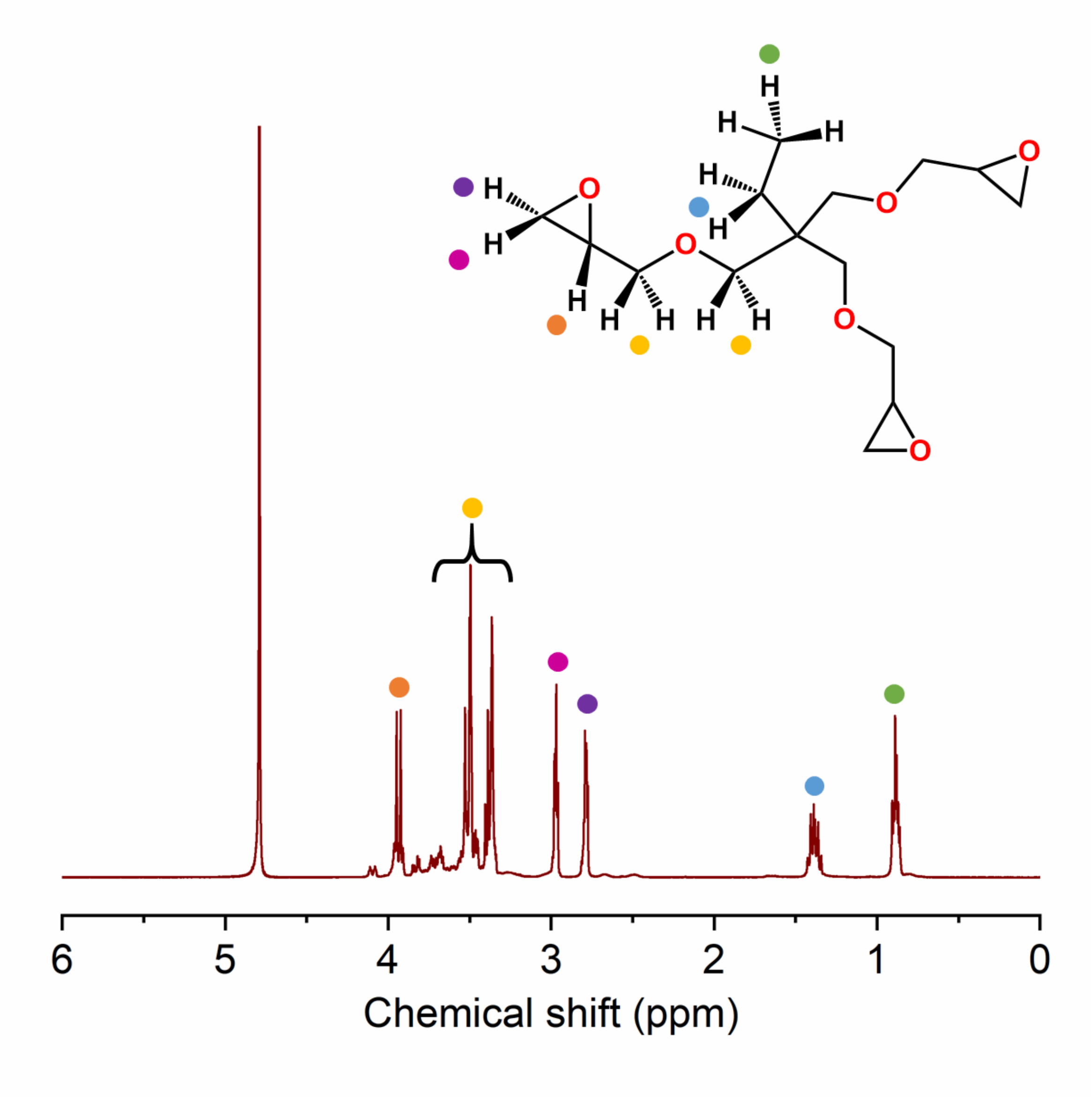

Figure S35. ^{1}H NMR spectrum of TMPTE in D_2O. Non-symmetric protons are drawn, while the rest are removed for clarity.

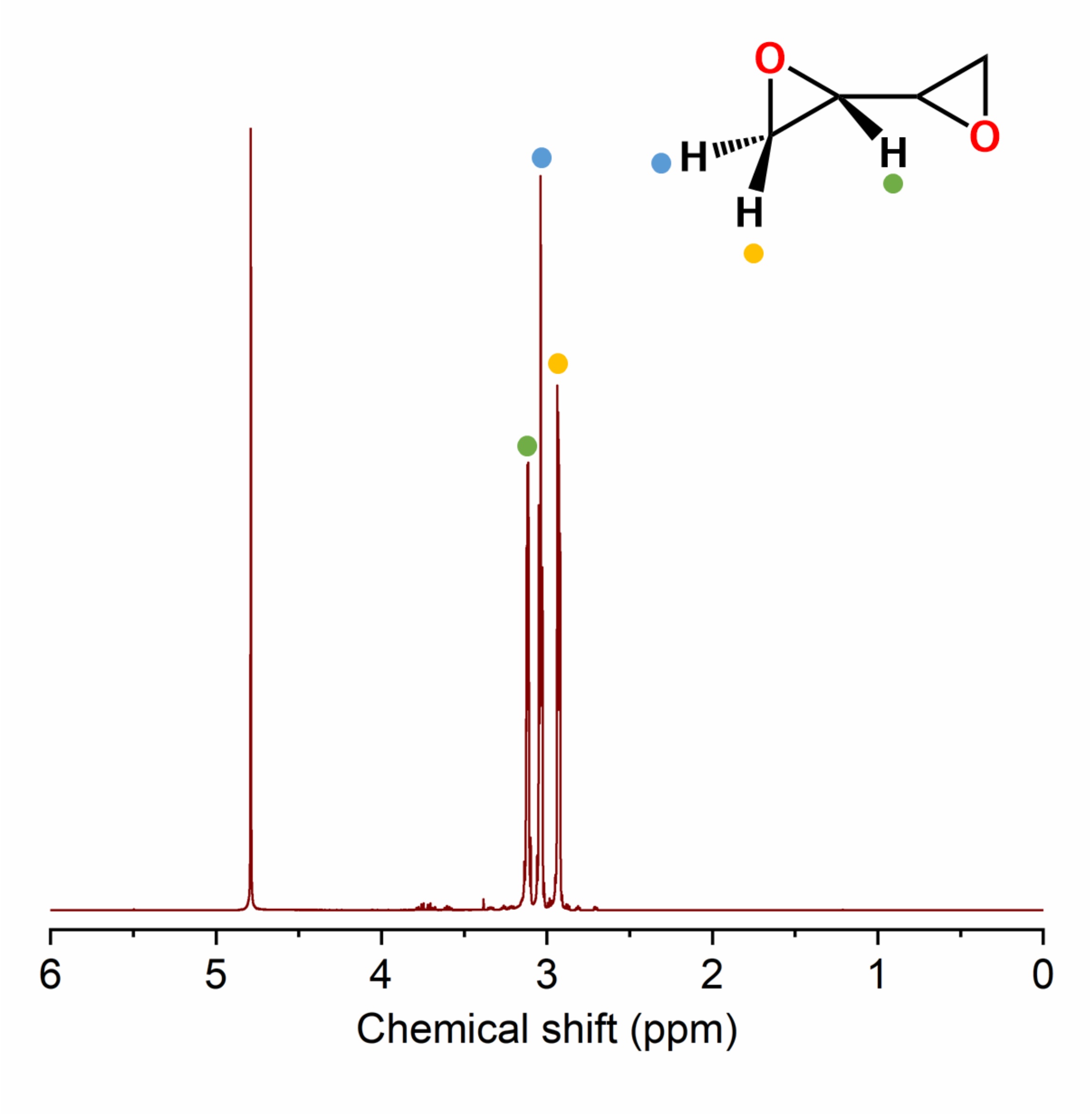

Figure S36. ^{1}H NMR spectrum of BDE in D_2O. Non-symmetric protons are drawn, while the rest are removed for clarity.

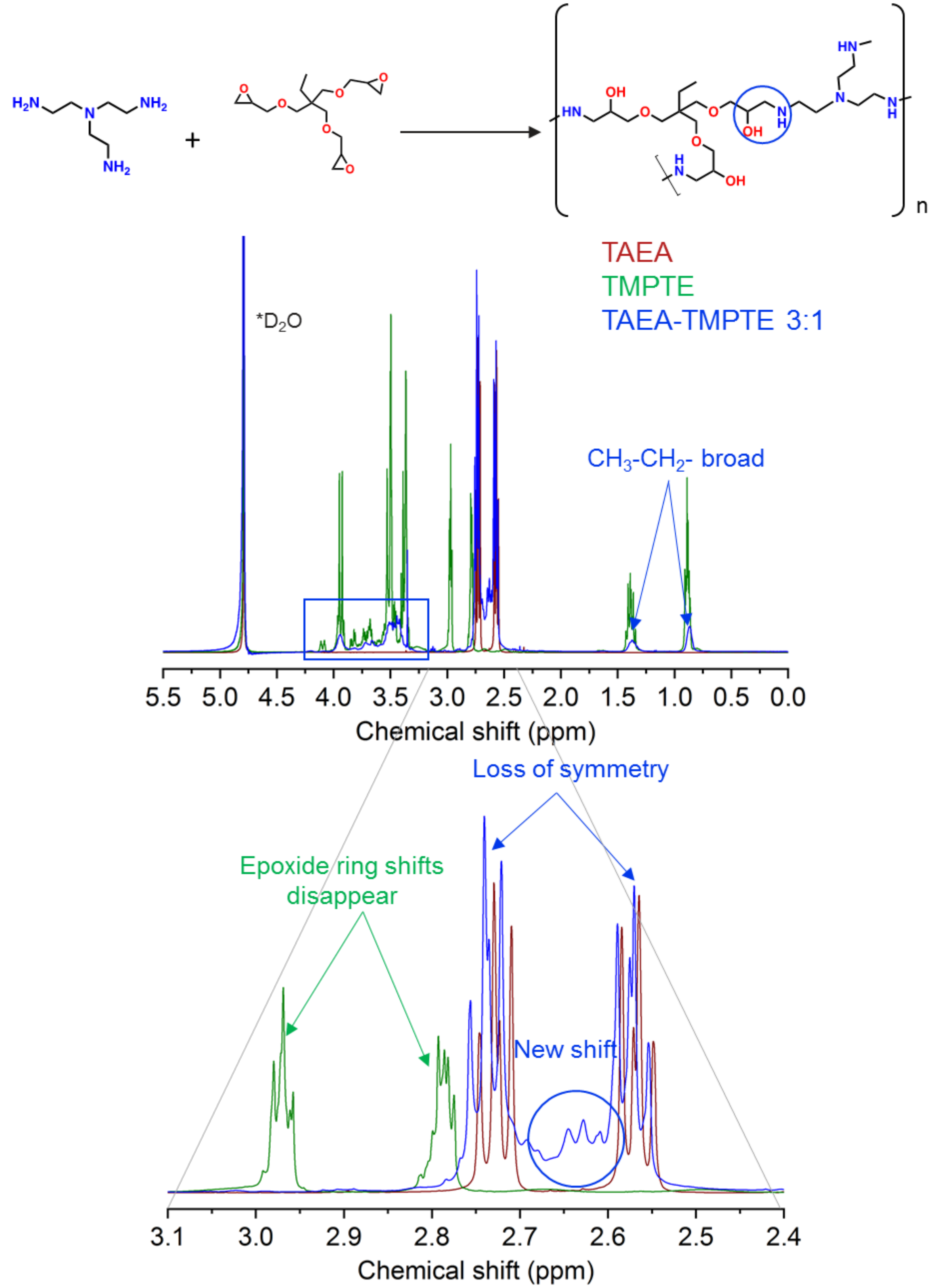

Figure S37. ^{1}H NMR spectra of TAEA, TMPTE and TAEA-TMPTE in D_2O.

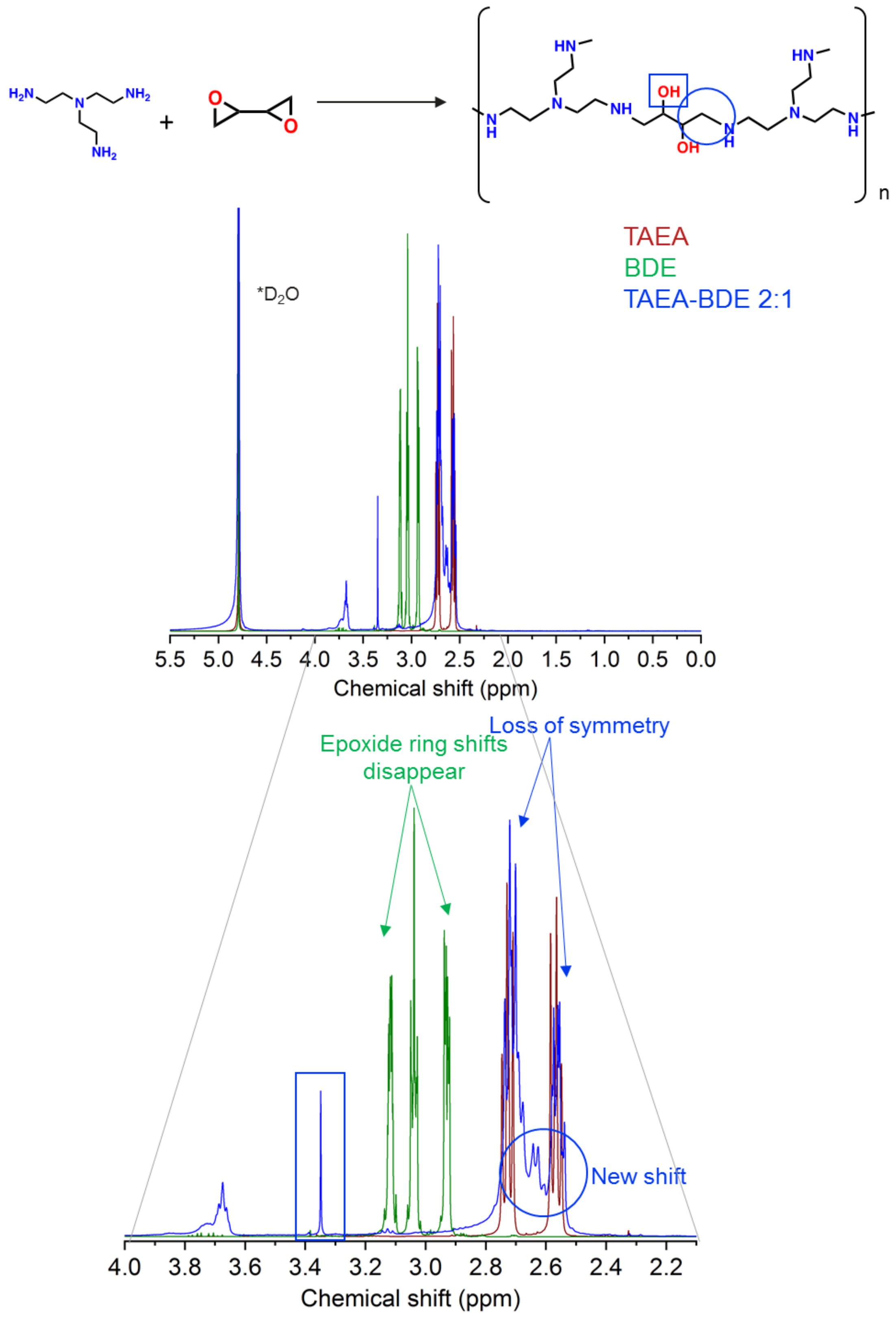

Figure S38. ^{1}H NMR spectra of TAEA, BDE and TAEA-BDE in D_2O.

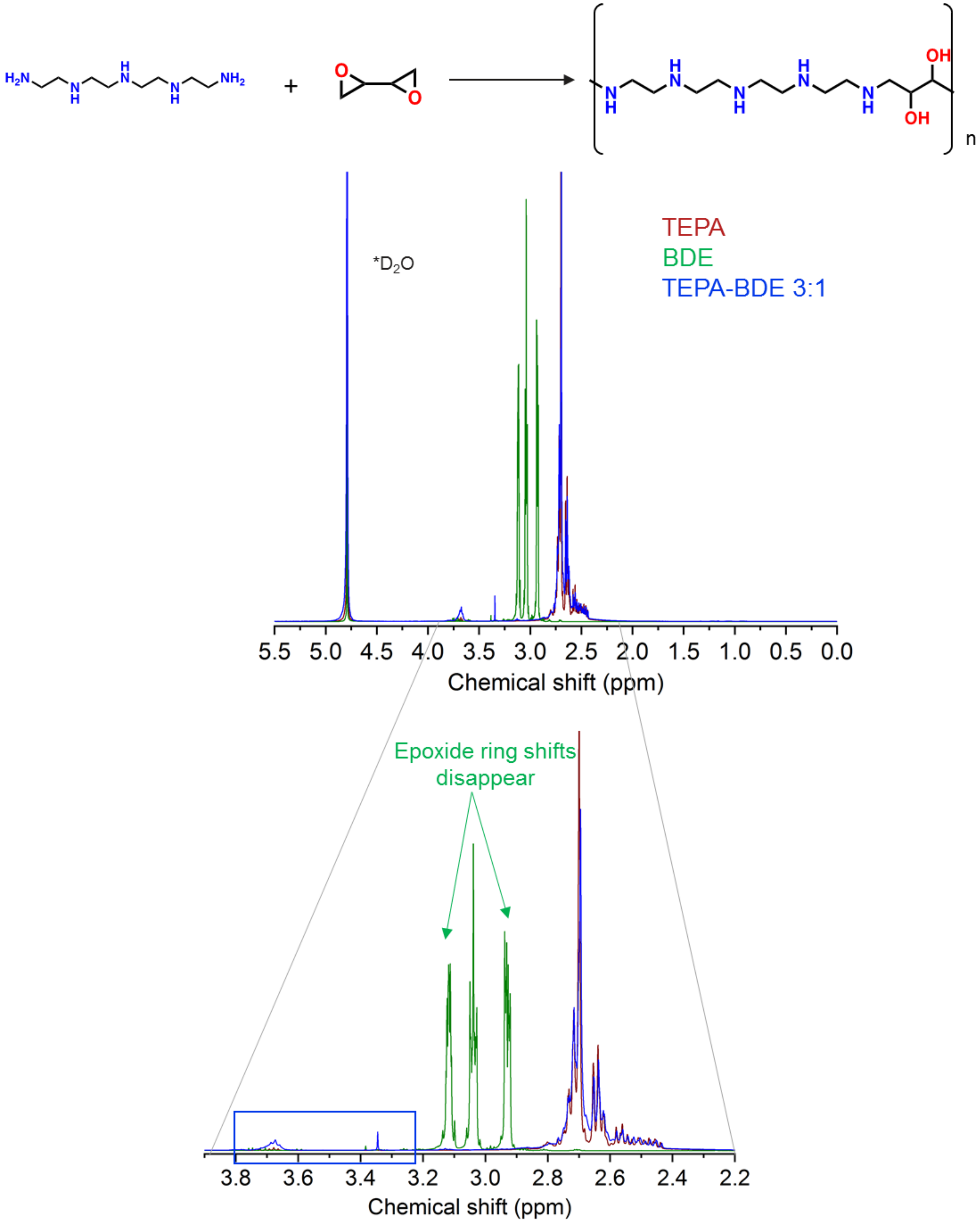

Figure S39. ^{1}H NMR spectra of TEPA, BDE and TEPA-BDE in D_2O.

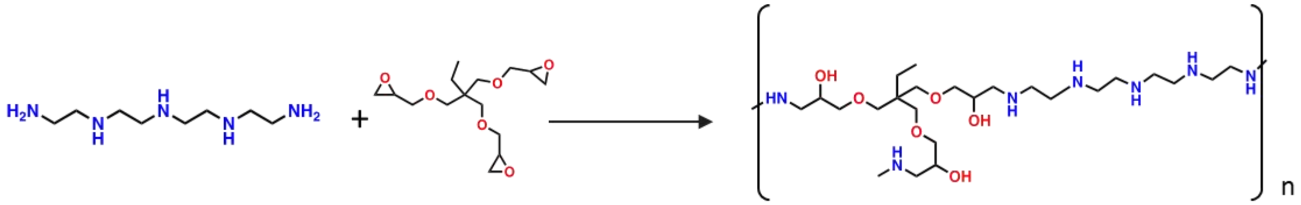

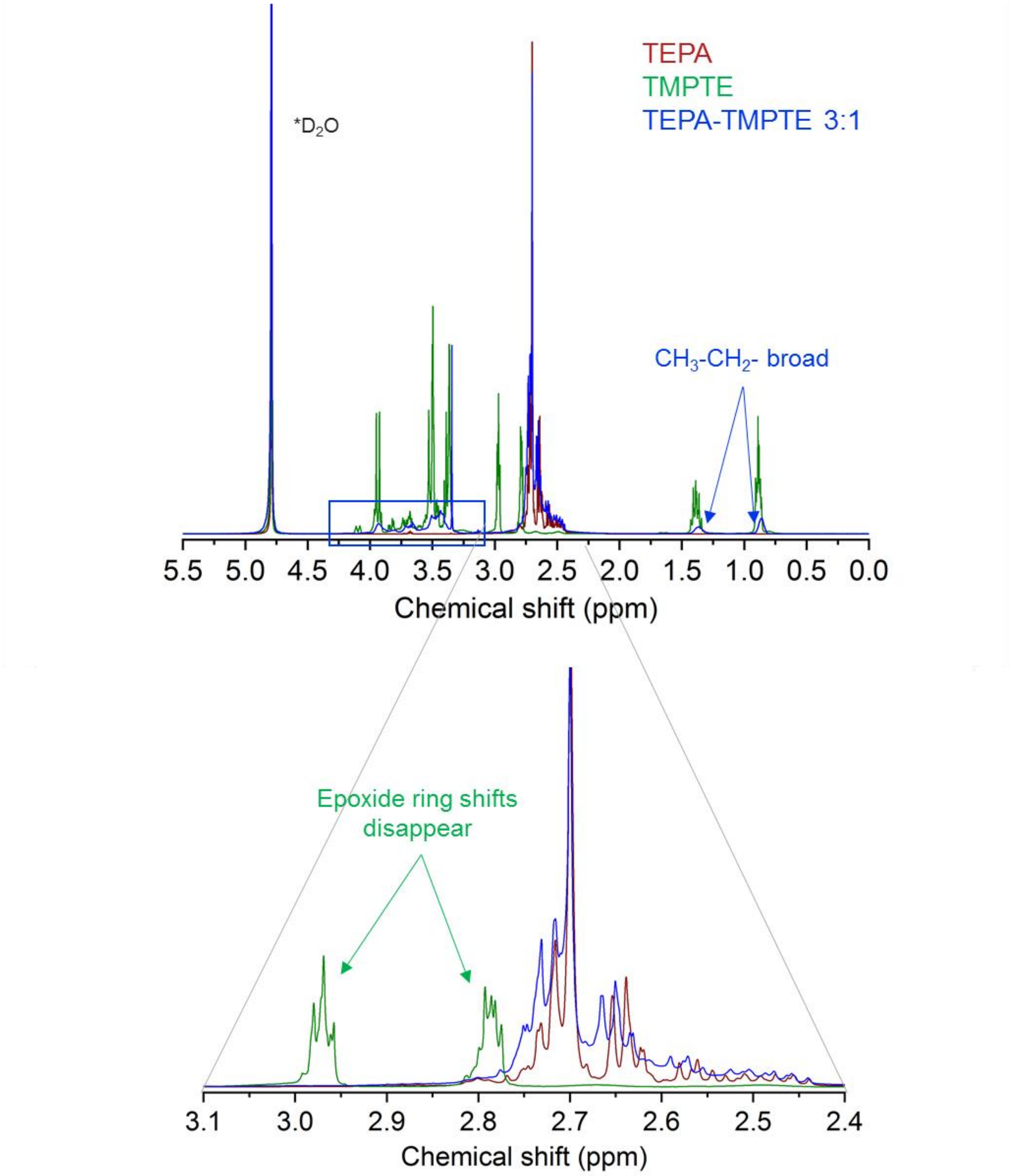

Figure S40. ^{1}H NMR spectra of TEPA, TMPTE and TEPA-TMPTE in D_2O.

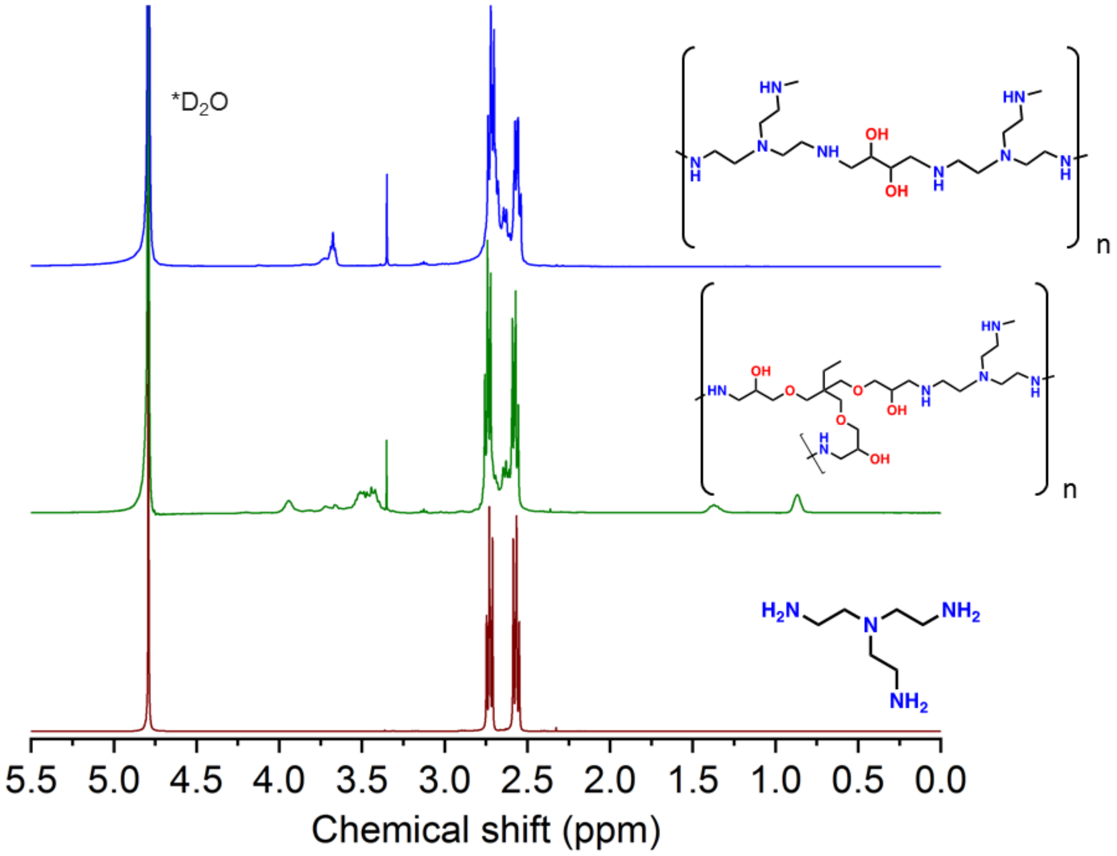

Figure S41. ^{1}H NMR spectra of TAEA (red) and the potential branched polymers TAEA-BDE (blue) and TAEA-TMPTE (green) in D_2O.

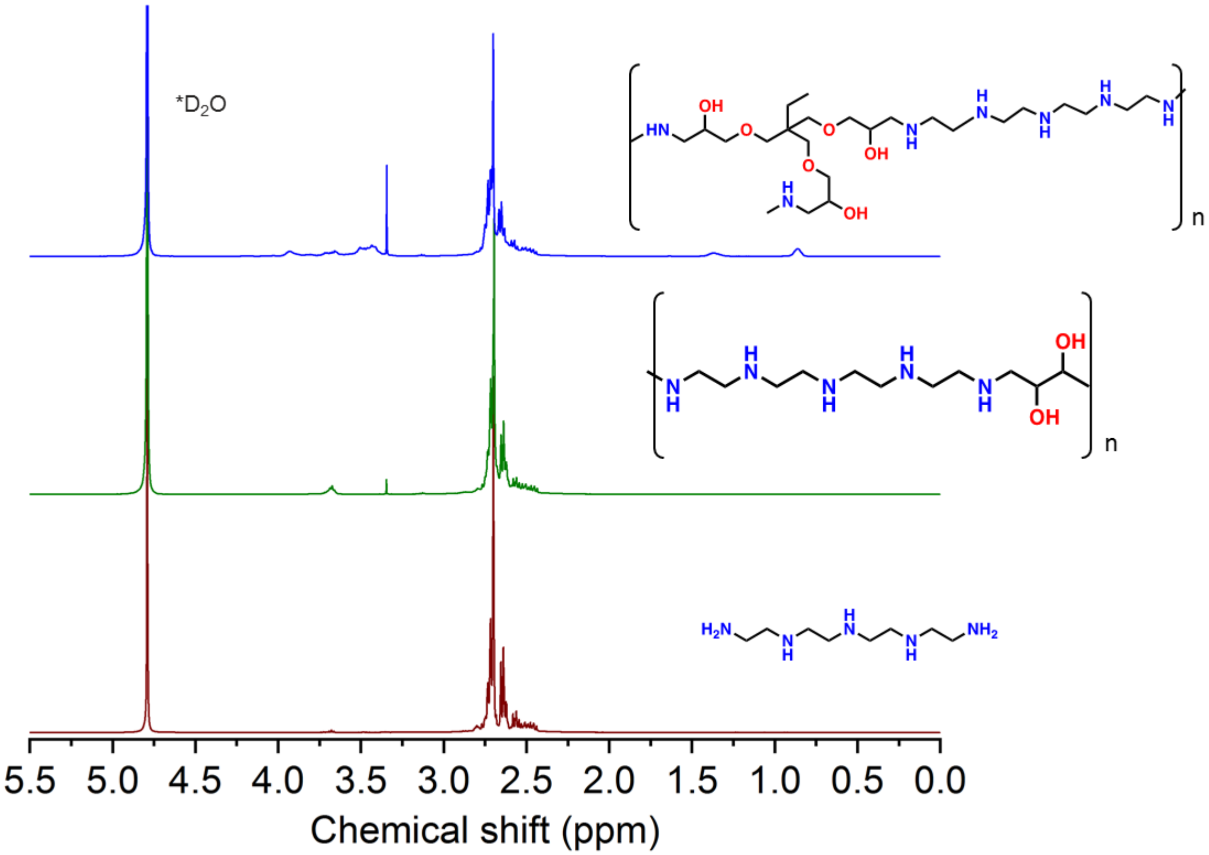

Figure S42. ^{1}H NMR spectra of TEPA (red) and the potential branched TEPA-TMPTE (blue) and linear TEPA-BDE (green) polymers and in D_2O.

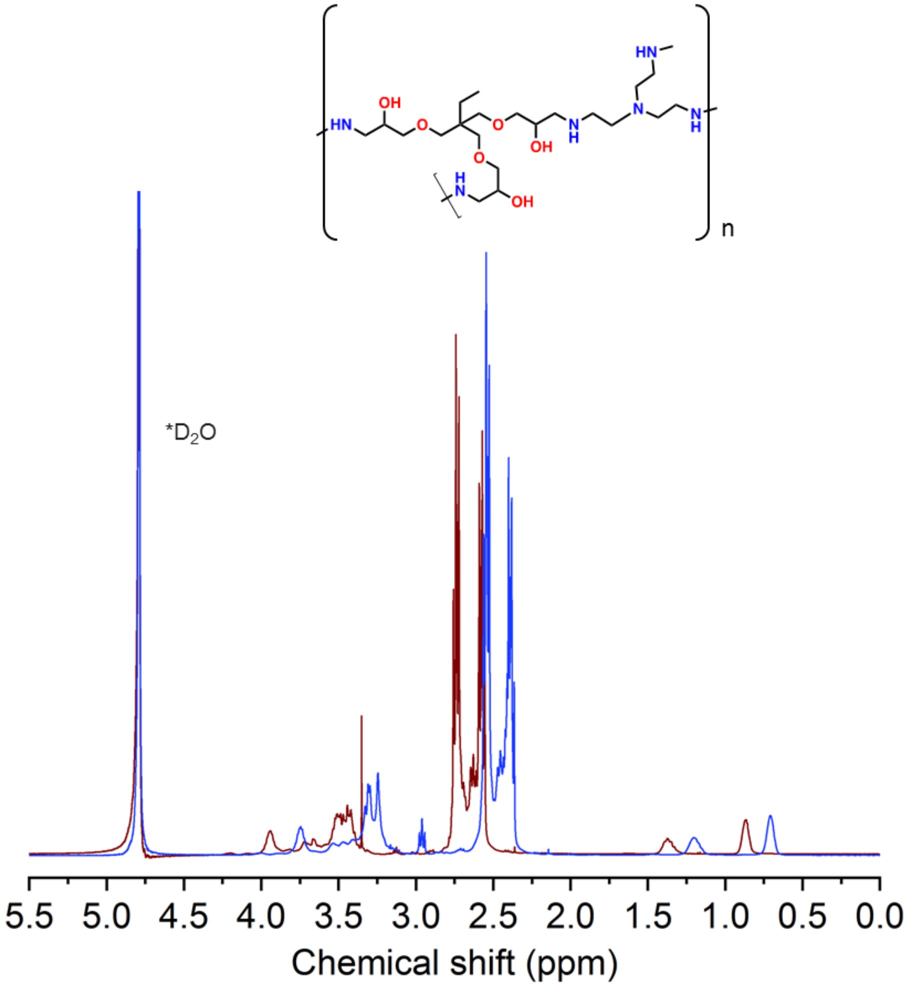

Figure S43. ^{1}H NMR spectra of TAEA-TMPTE polymer dissolved in D_2O (red) and in D_2O/NaOD solution (60 µL NaOD 40% in D_2O in 500 µL D_2O) (blue).

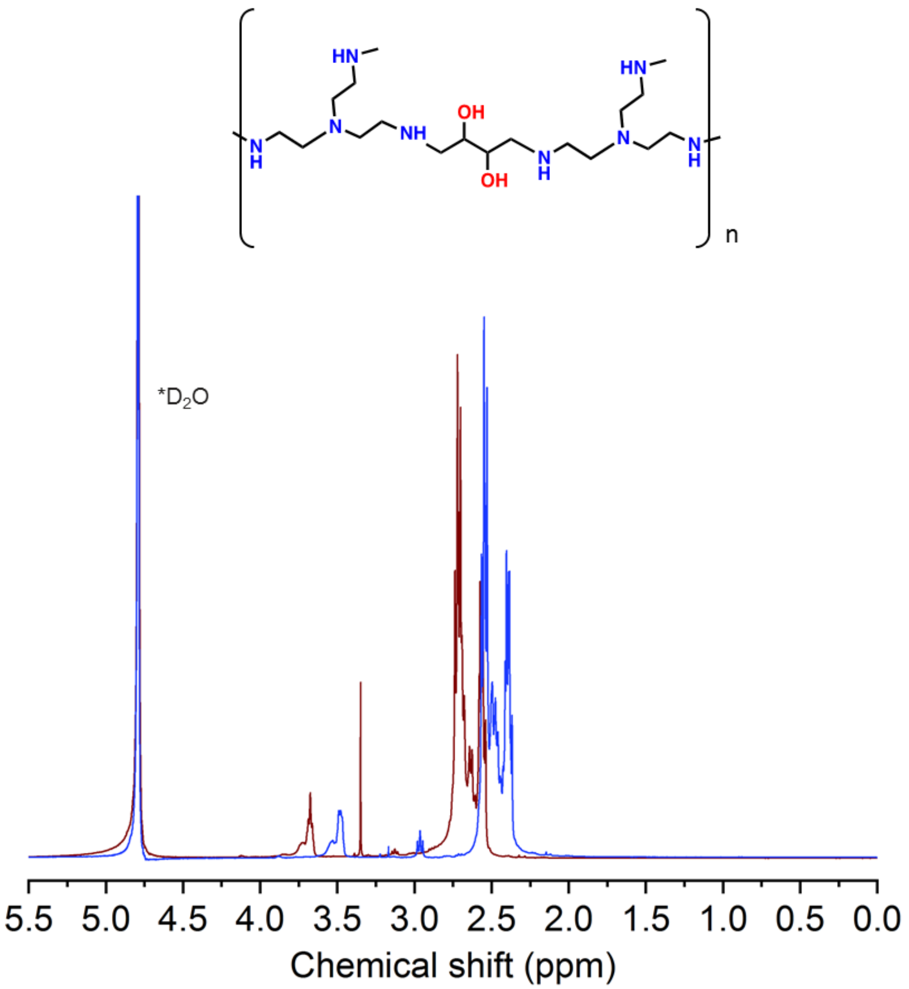

Figure S44. ^{1}H NMR spectra of TAEA-BDE polymer dissolved in D_2O (red) and in D_2O/NaOD solution (60 µL NaOD 40% in D_2O in 500 µL D_2O) (blue).

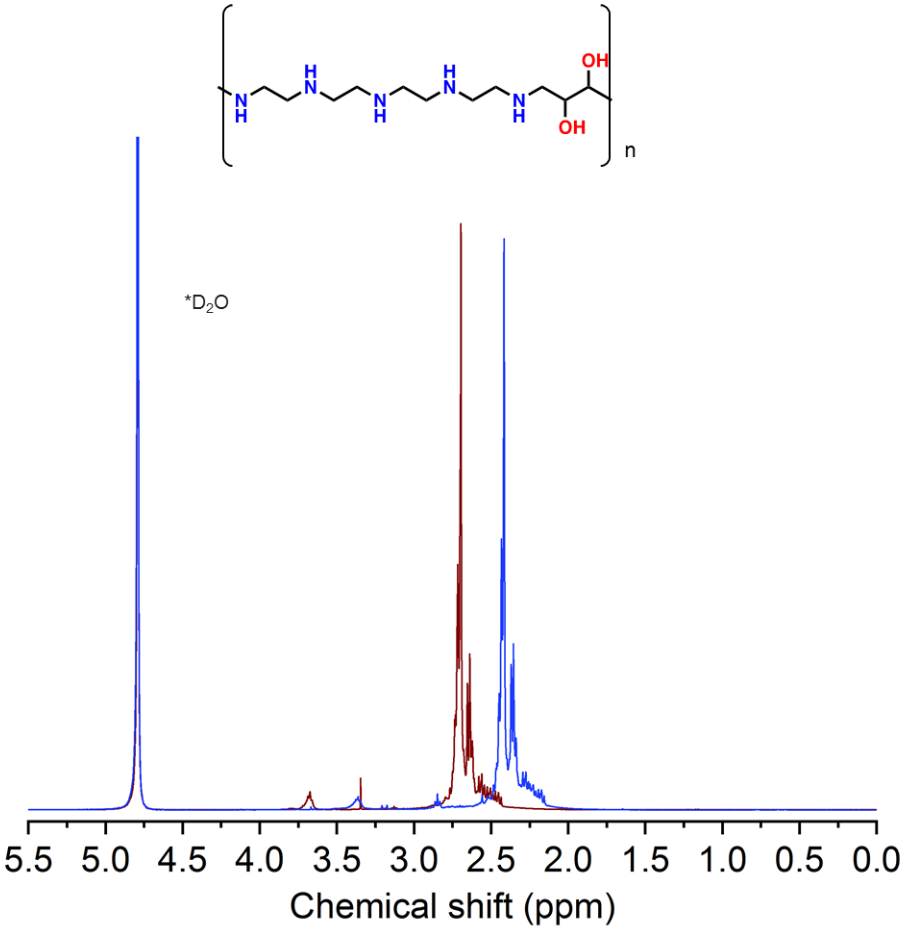

Figure S45. ^{1}H NMR spectra of TEPA-BDE polymer dissolved in D_2O (red) and in D_2O/NaOD solution (60 µL NaOD 40% in D_2O in 500 µL D_2O) (blue).

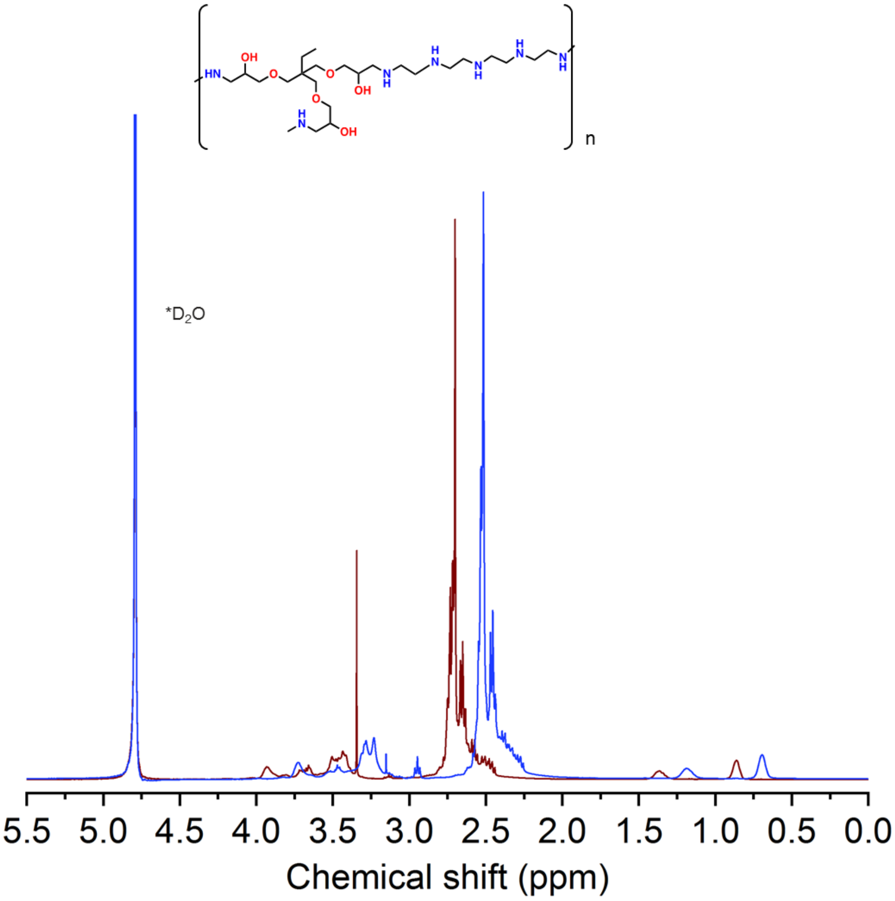

Figure S46. ^{1}H NMR spectra of TEPA-TMPTE polymer dissolved in D_2O (red) and in D_2O/NaOD solution (60 µL NaOD 40% in D_2O in 500 µL D_2O) (blue).

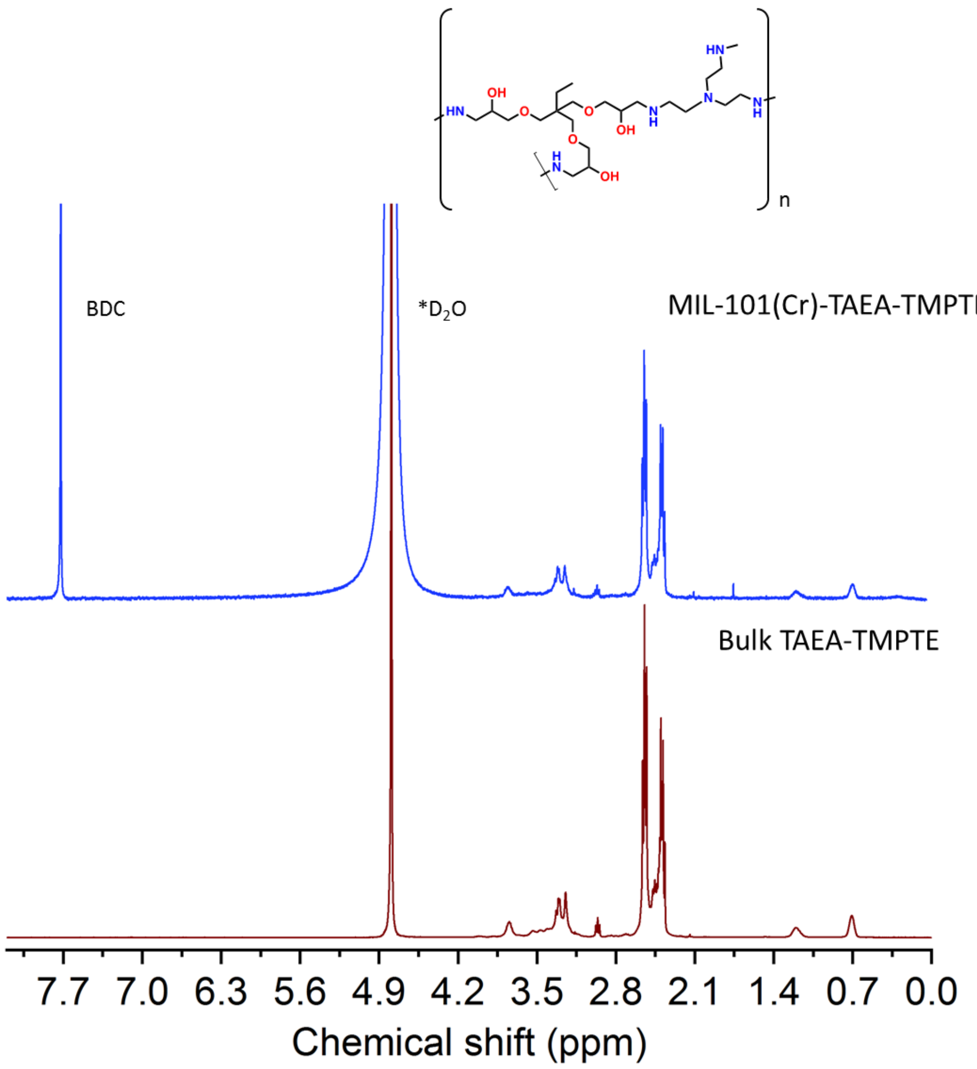

Figure S47. ^{1}H NMR spectra of Cr-BDC-TAEA-TMPTE digested in D_2O/NaOD solution (60 µL NaOD 40% in D_2O in 500 µL D_2O) (blue) compared with the TAEA-TMPTE polymer.

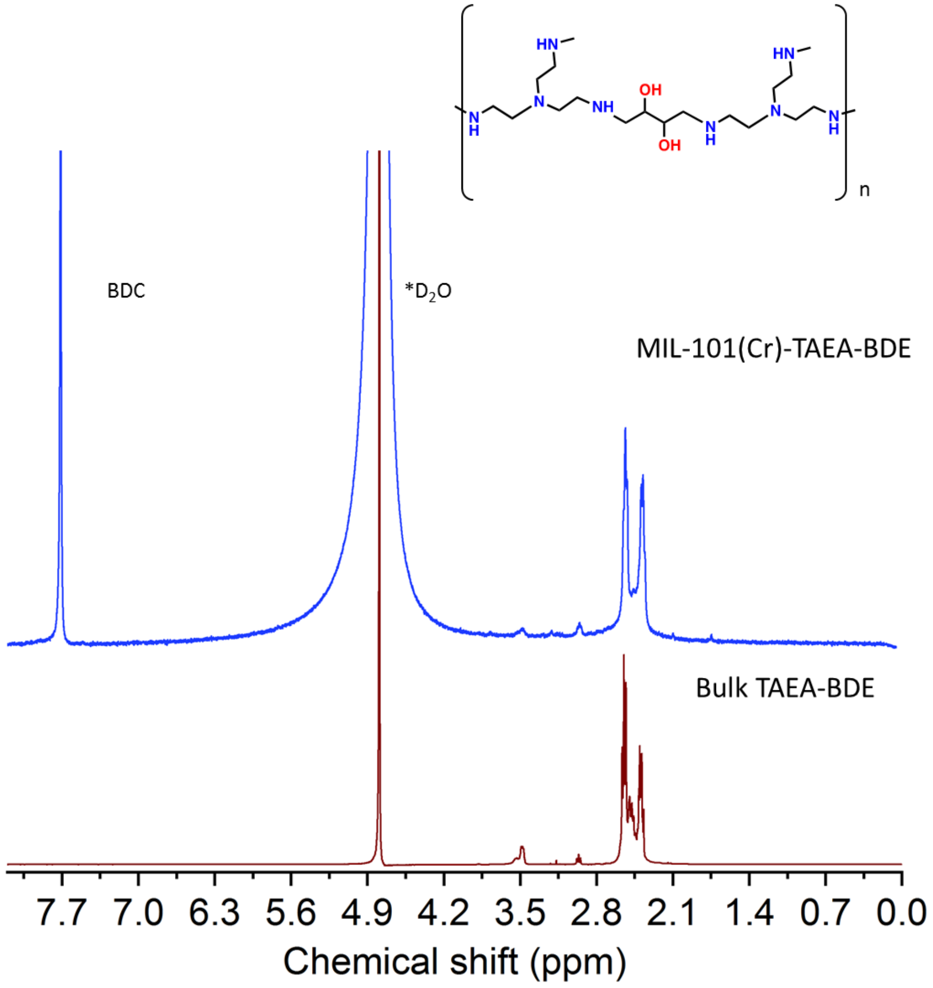

Figure S48. ^{1}H NMR spectra of Cr-BDC-TAEA-BDE digested in D_2O/NaOD solution (60 µL NaOD 40% in D_2O in 500 µL D_2O) (blue) compared with the TAEA-BDE polymer.

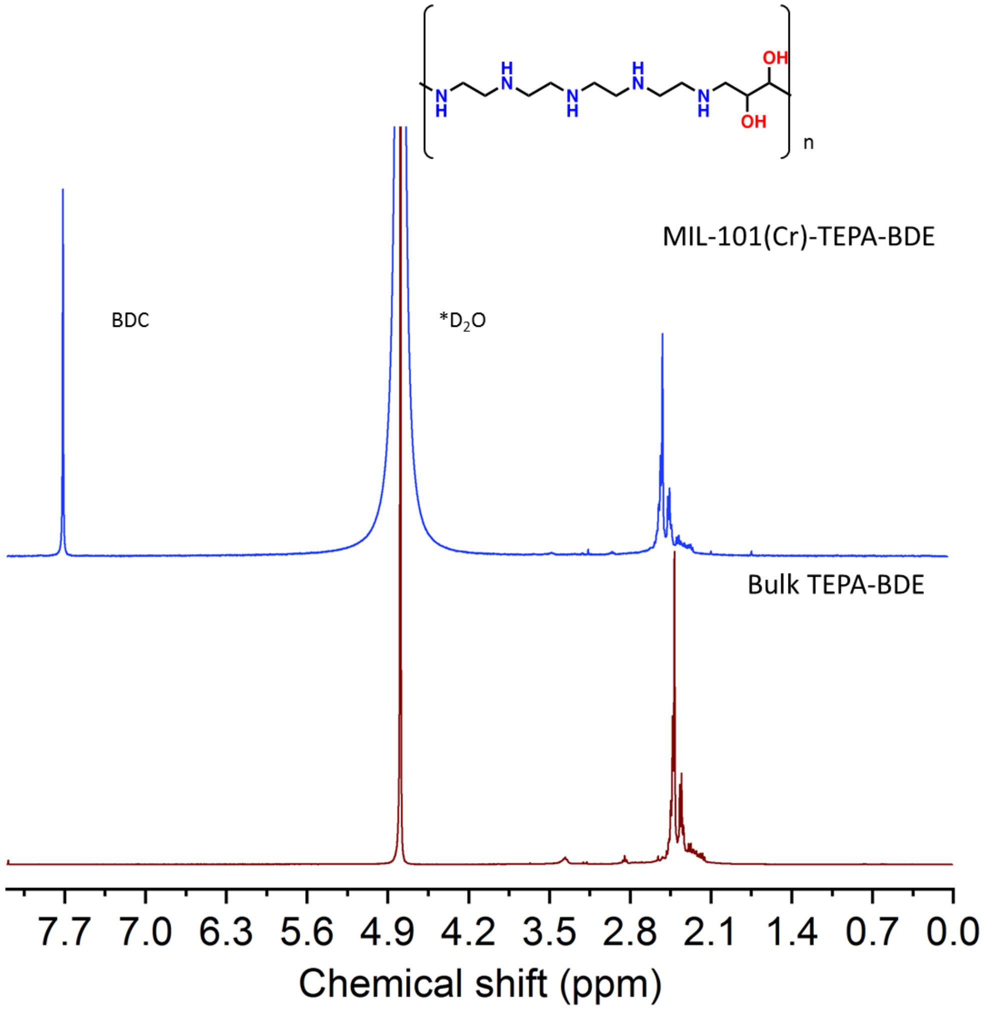

Figure S49. ^{1}H NMR spectra of Cr-BDC-TEPA-BDE digested in D_2O/NaOD solution (60 µL NaOD 40% in D_2O in 500 µL D_2O) (blue) compared with the TEPA-BDE polymer.

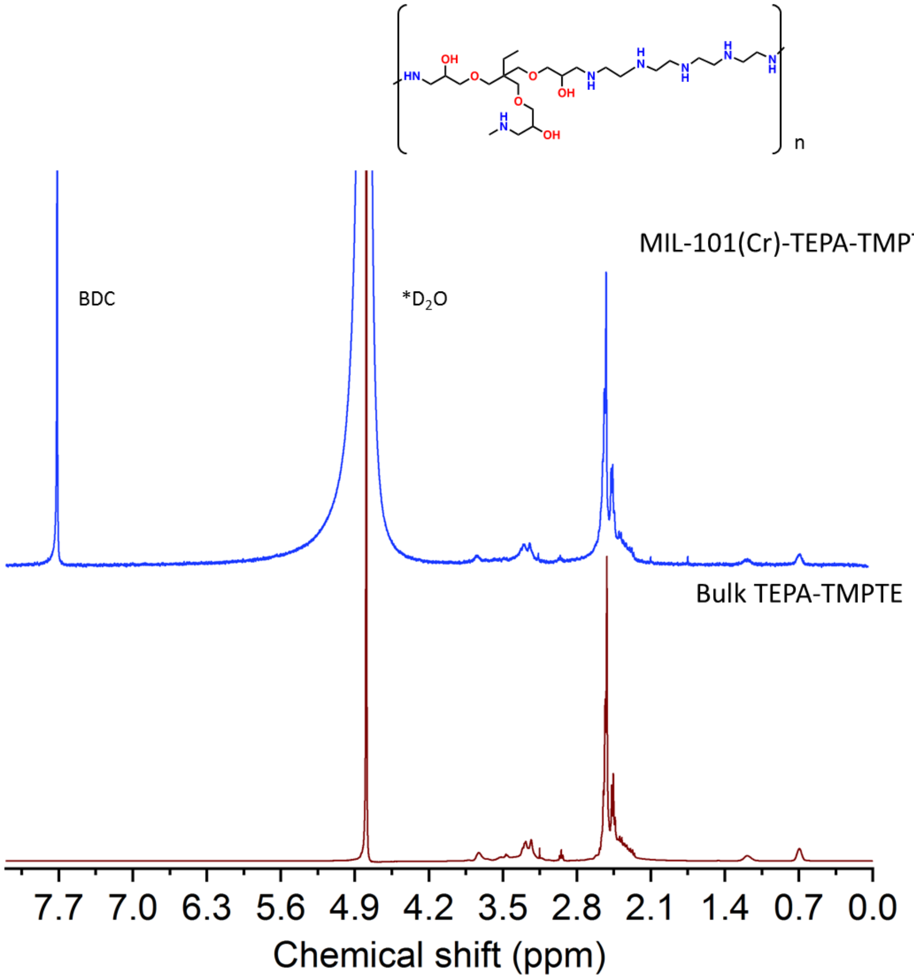

Figure S50. ^{1}H NMR spectra of Cr-BDC-TEPA-TMPTE digested in D_2O/NaOD solution (60 µL NaOD 40% in D_2O in 500 µL D_2O) (blue) compared with the TEPA-TMPTE polymer.

ESI-MS characterization

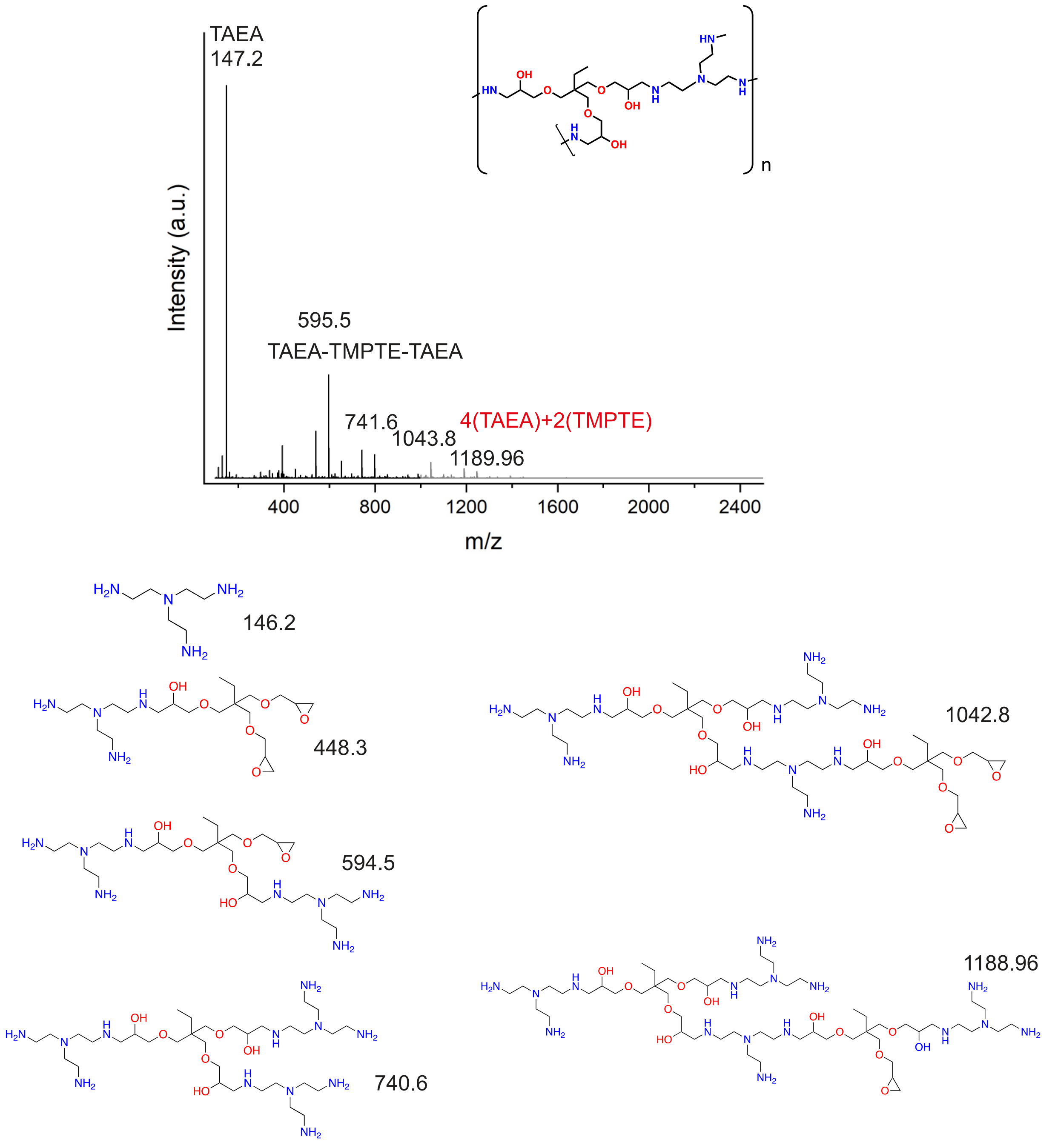

Figure S51. ESI-MS spectrum of TAEA-TMPTE bulk polymer in MeOH and potential structures derived from the amine-epoxide crosslinking. Note that the different plot color at higher m/z values indicates enlarged intensity.

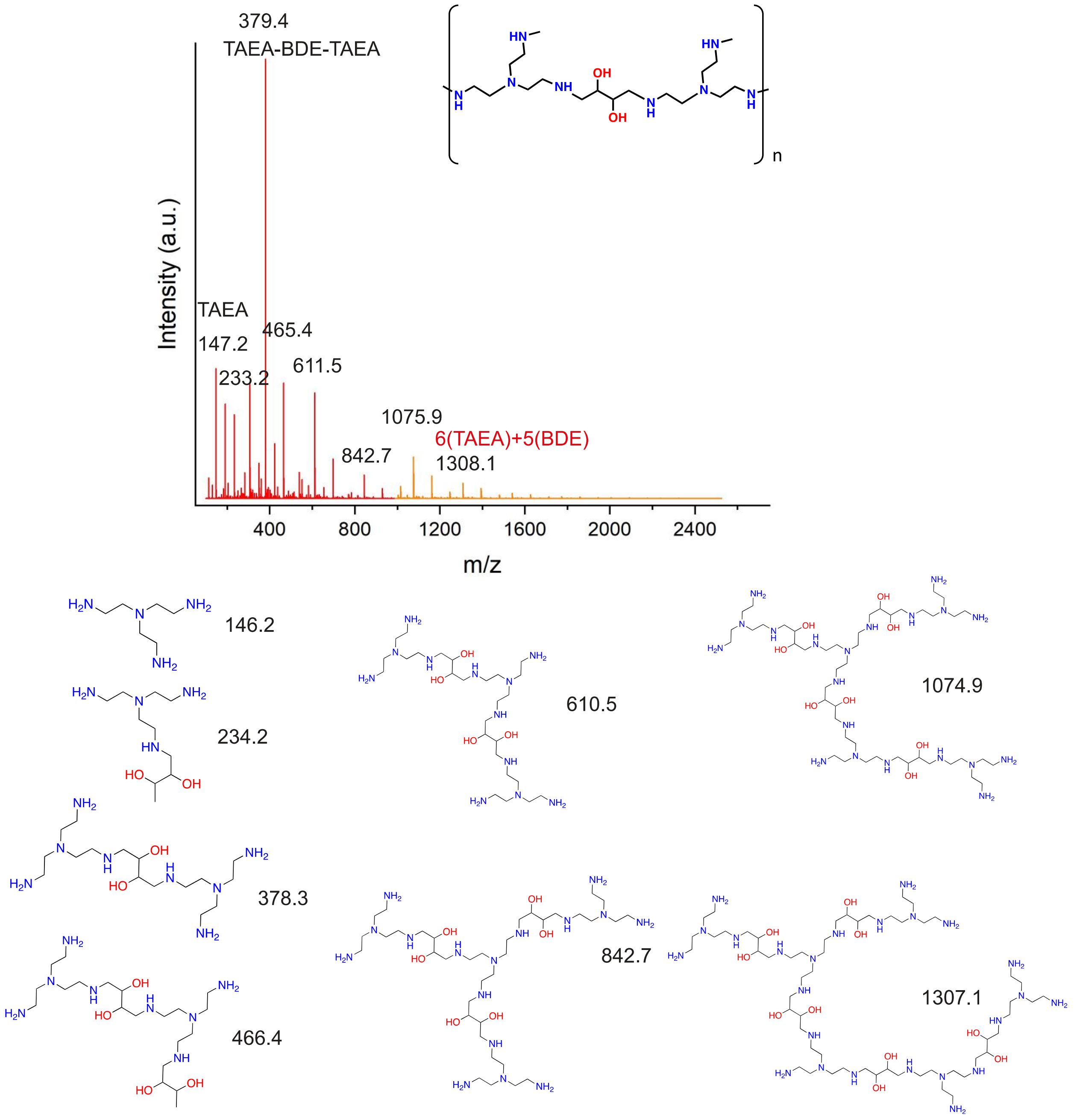

Figure S52. ESI-MS spectrum of TAEA-BDE bulk polymer in MeOH and potential structures derived from the amine-epoxide crosslinking. Note that the different plot color at higher m/z values indicates enlarged intensity.

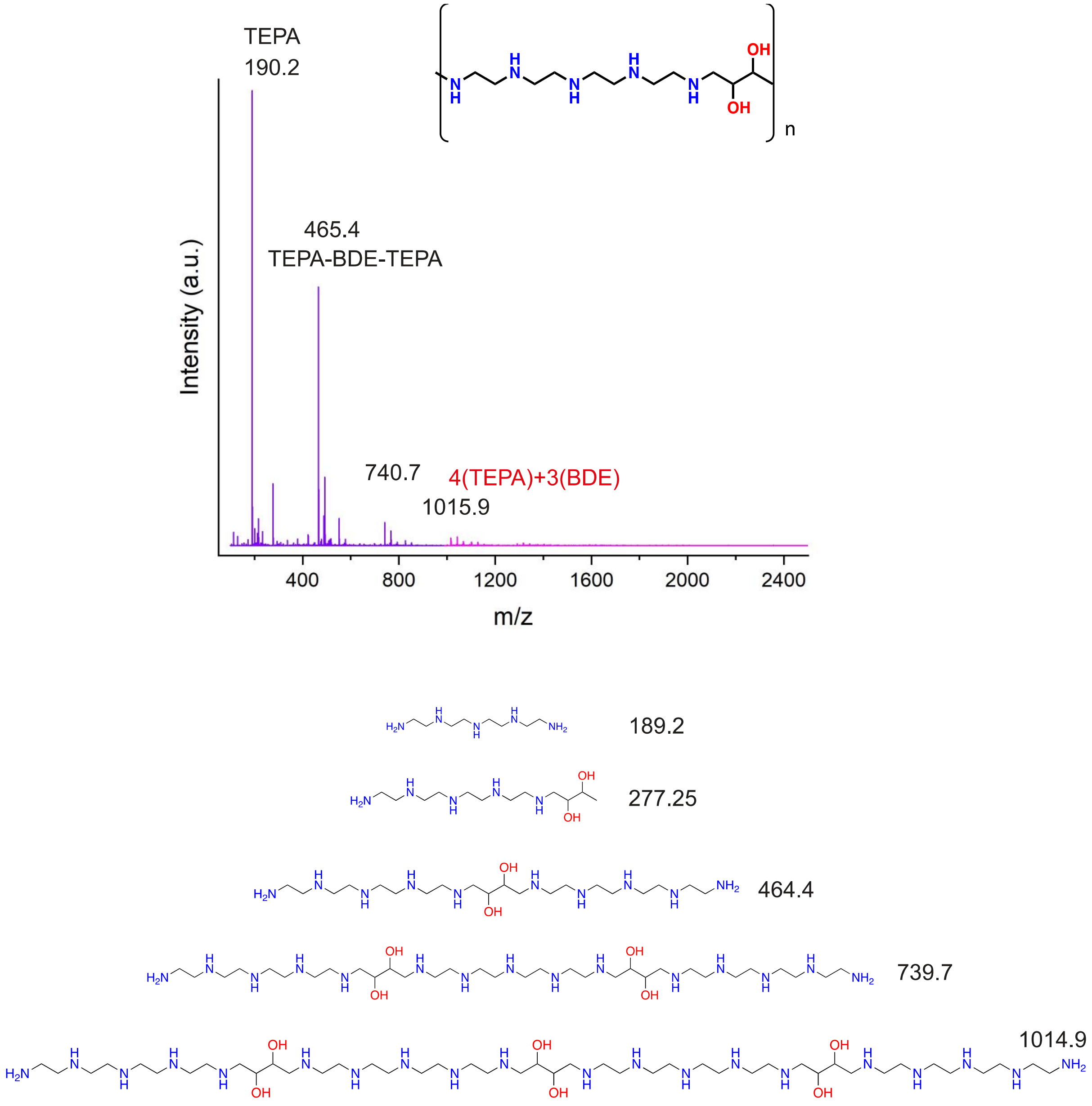

Figure S53. ESI-MS spectrum of TEPA-BDE bulk polymer in MeOH and potential structures derived from the amine-epoxide crosslinking. Note that the different plot color at higher m/z values indicates enlarged intensity.

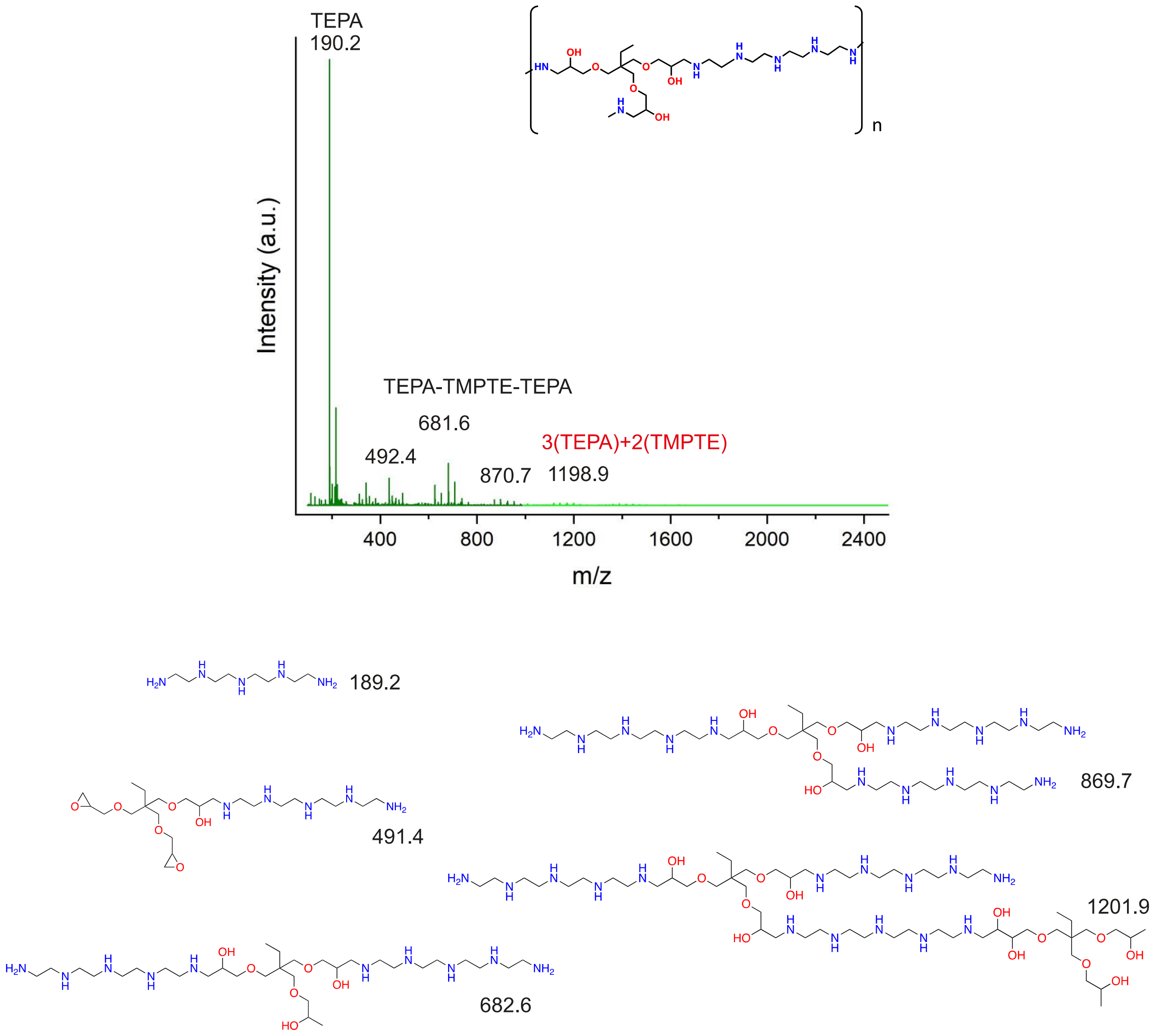

Figure S54. ESI-MS spectrum of TEPA-TMPTE bulk polymer in MeOH and potential structures derived from the amine-epoxide crosslinking. Note that the different plot color at higher m/z values indicates enlarged intensity.

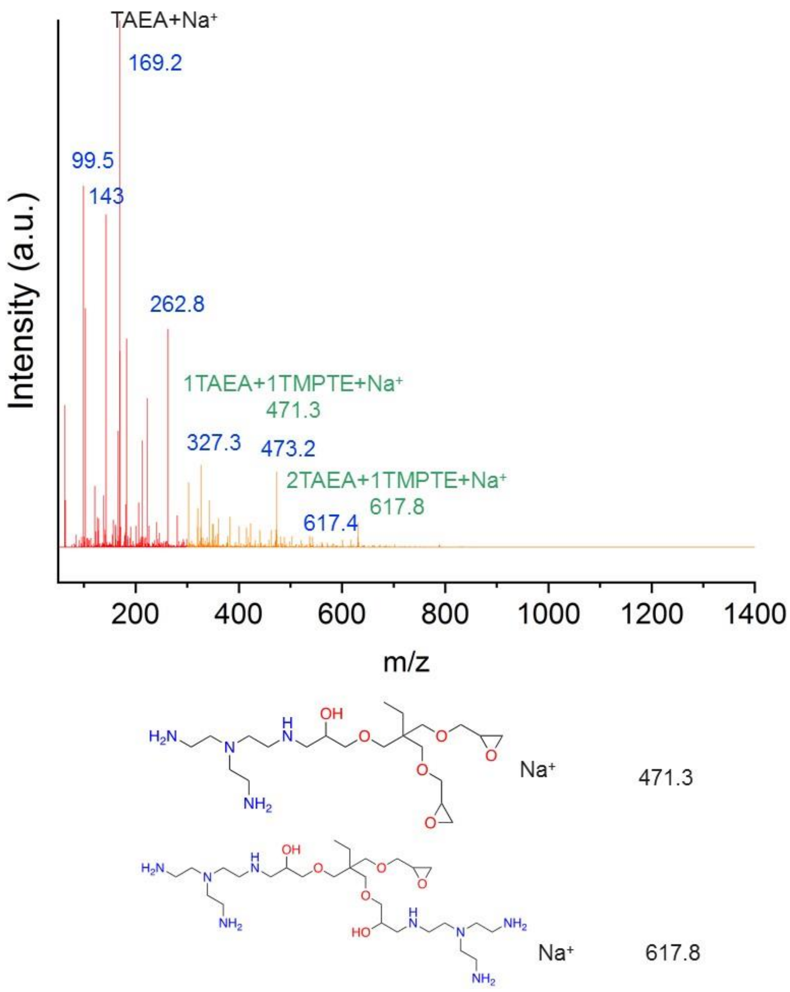

Figure S55. ESI-MS spectrum of polymer liberated from Cr-BDC-TAEA-TMPTE in MeOH/NaOH and potential structures derived from the amine-epoxide crosslinking inside the MOF pores. Note that the different plot color at higher m/z values indicates enlarged intensity.

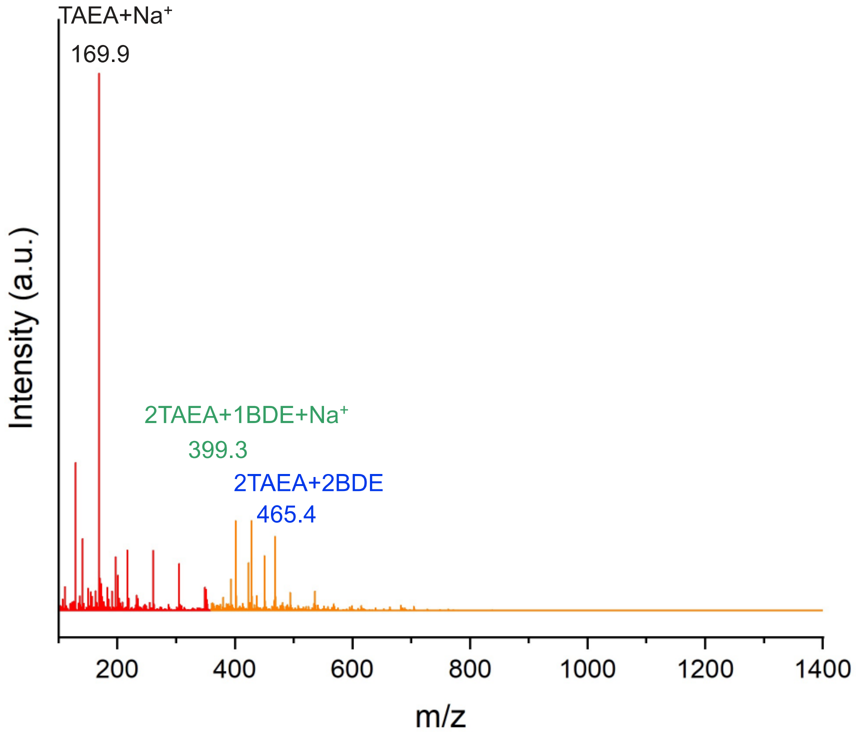

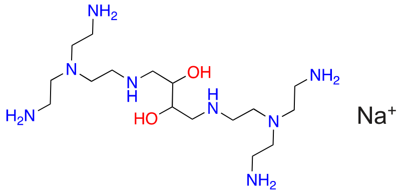

399.3

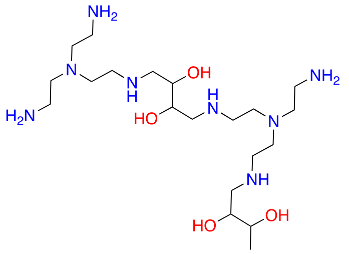

465.4

Figure S56. ESI-MS spectrum of polymer liberated from Cr-BDC-TAEA-BDE in MeOH/NaOH and potential structures derived from the amine-epoxide crosslinking inside the MOF pores. Note that the different plot color at higher m/z values indicates enlarged intensity.

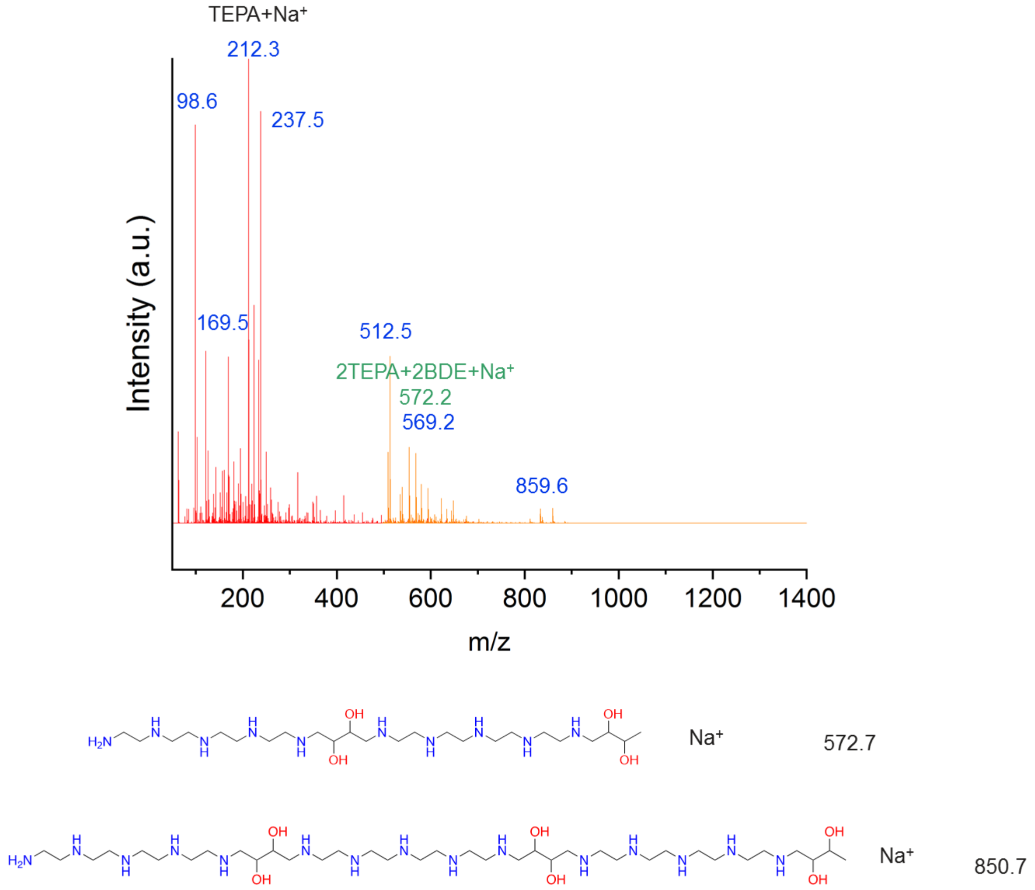

Figure S57. ESI-MS spectrum of polymer liberated from Cr-BDC-TEPA-BDE in MeOH/NaOH and potential structures derived from the amine-epoxide crosslinking inside the MOF pores. Note that the different plot color at higher m/z values indicates enlarged intensity.

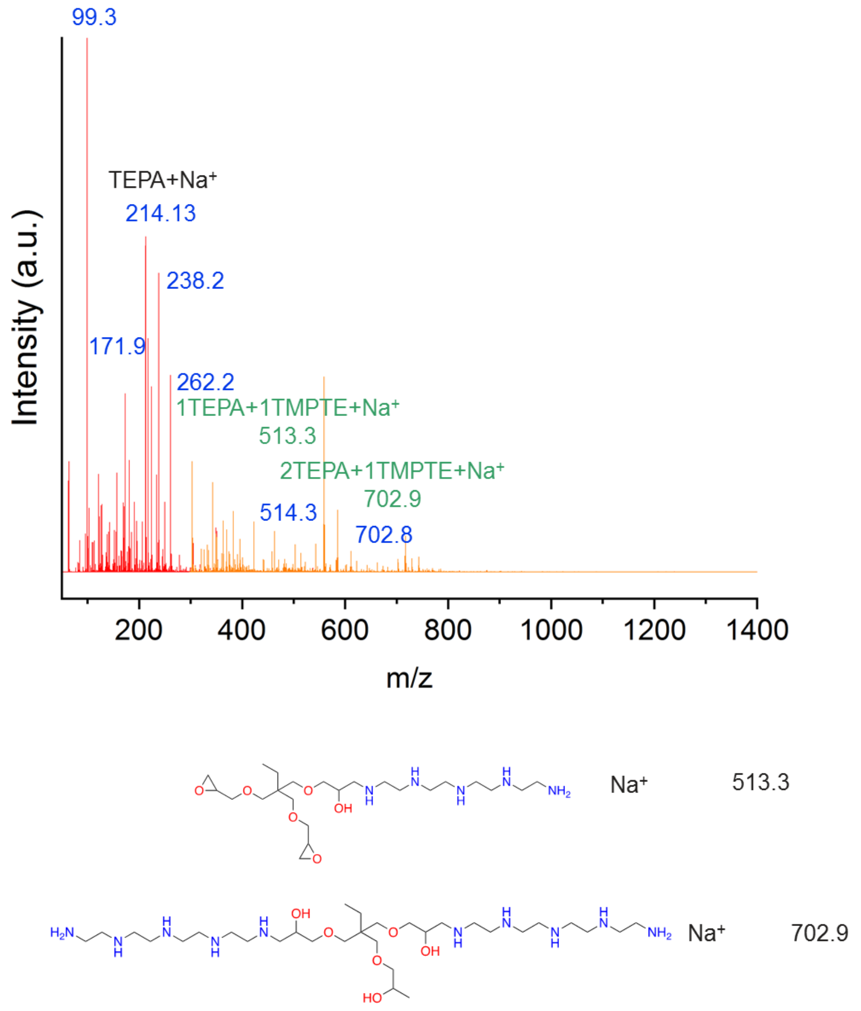

Figure S58. ESI-MS spectrum of polymer liberated from Cr-BDC-TEPA-TMPTE in MeOH/NaOH and potential structures derived from the amine-epoxide crosslinking inside the MOF pores. Note that the different plot color at higher m/z values indicates enlarged intensity.

N_2 adsorption isotherms

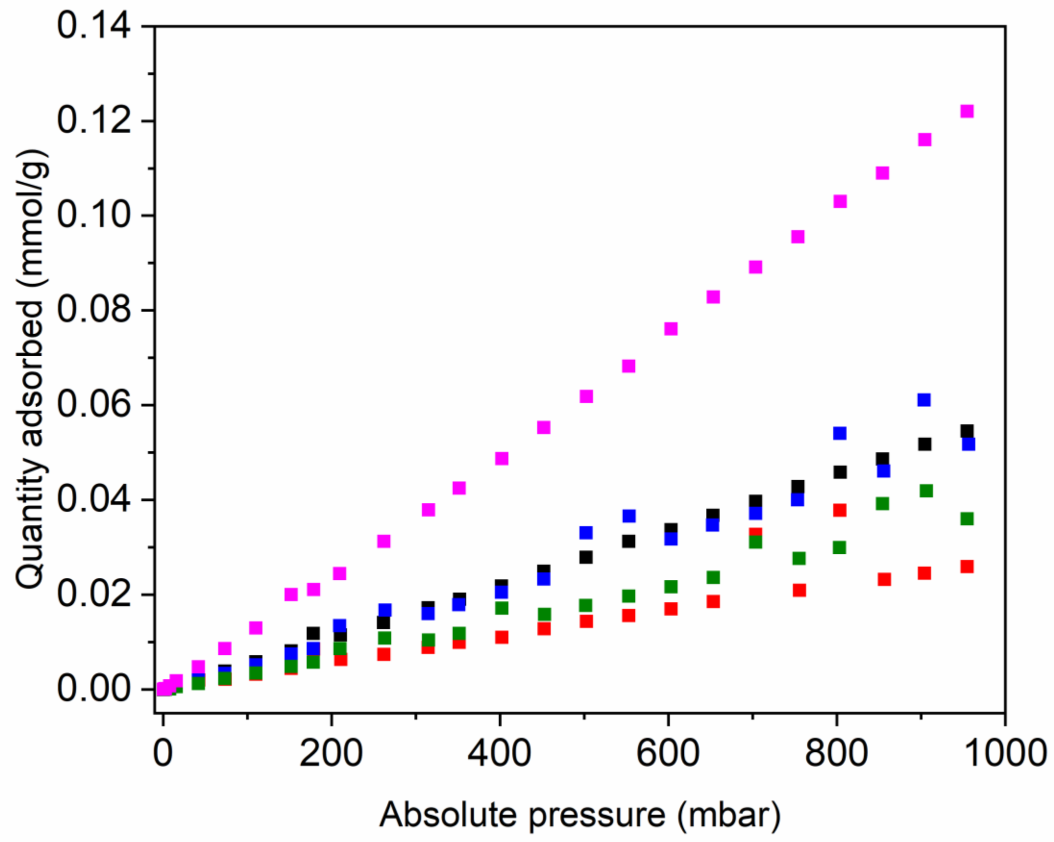

Figure S59. N_2 adsorption isotherms at 313 K of the bare Cr-BDC (magenta) compared with the Cr-BDC-amine-epoxide composites; TEPA-TMPTE (black), TEPA-BDE (red), TAEA-BDE (blue) and TAEA-TMPTE (green). The synthesis of these materials was done at larger scales (550 mg).

CO_2 adsorption at different temperatures and Heat of Adsorption

	Temperature (K)	q CO_2 @0.15 bar (mmol/g)
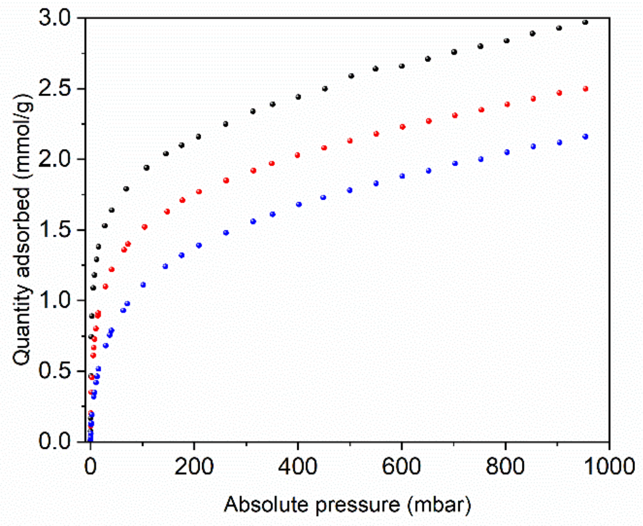	313	2.05
	333	1.65
	353	1.26

Figure S60. Adsorption isotherm plots at 313 (black), 333 (red) and 353 K (blue) of Cr-BDC-TAEA-TMPTE and their respective adsorbed amounts at 0.15 bar.

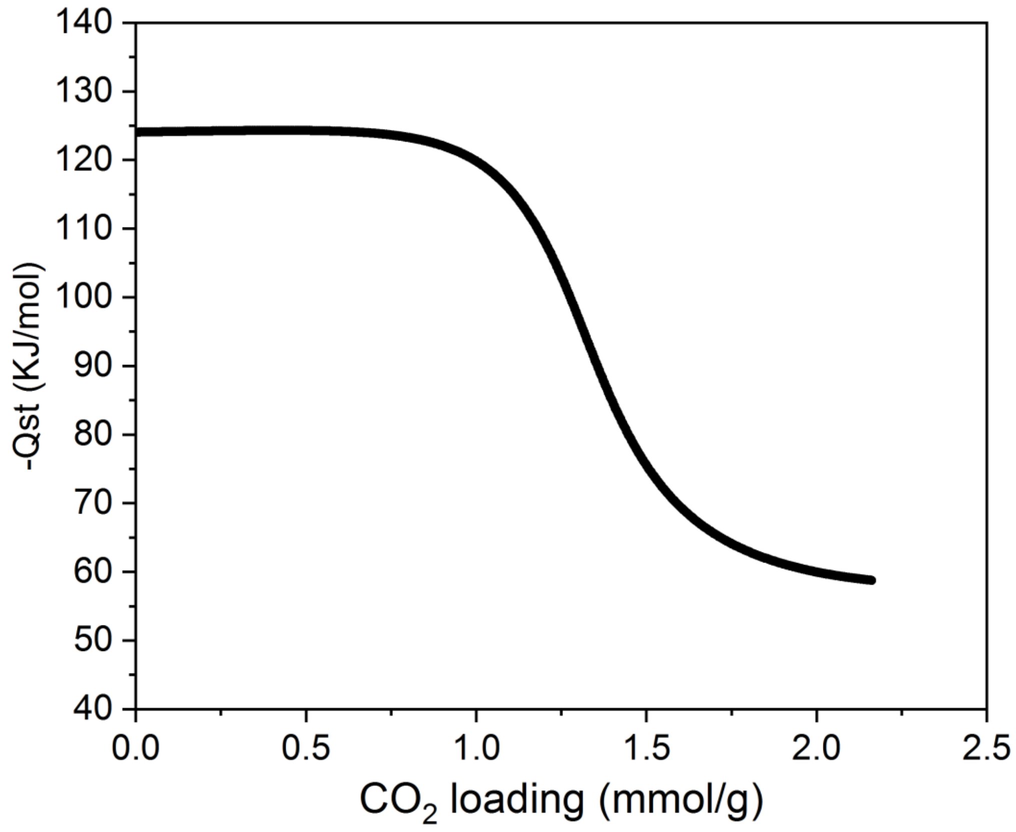

Figure S61. Isosteric heat of CO_2 adsorption of Cr-BDC-TAEA-TMPTE.

Table S10. Parameters of Cr-BDC-TAEA-TMPTE composite for the Dual-Site Langmuir fittings with the experimental isotherms.

**Temperature**	$q_{sat,1}$	b_1	$q_{sat,2}$	b_2
313 K	2.1	3.30E-5	1.32	0.011
333 K	2.1	1.31E-5	1.33	0.0014
353 K	2.1	8.86E-6	1.2	0.00041

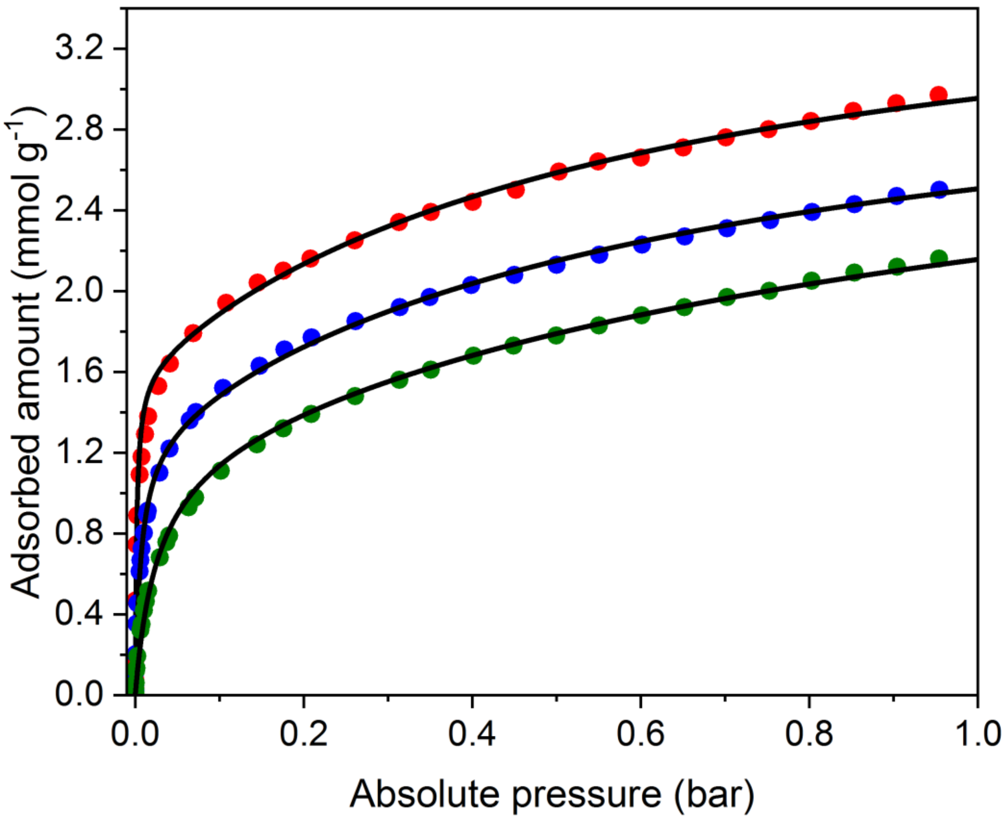

Figure S62. Isotherms of Cr-BDC-TAEA-TMPTE at 3 different temperatures; 313 K (red dots), 333 K (blue dots), 353 K (green dots), and Dual-Site Langmuir fitting (black line).

	Temperature (K)	q CO_2 @0.15 bar (mmol/g)
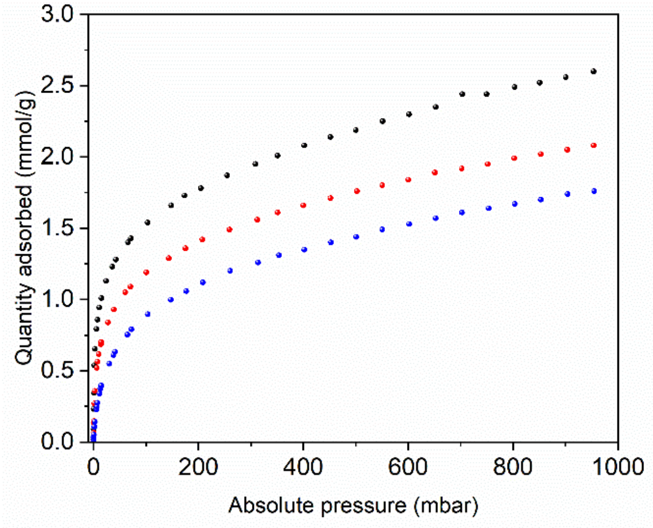	313	1.67
	333	1.31
	353	1.02

Figure S63. Adsorption isotherm plots at 313 (black), 333 (red) and 353 K (blue) of Cr-BDC-TEPA-BDE and their respective adsorbed amounts at 0.15 bar.

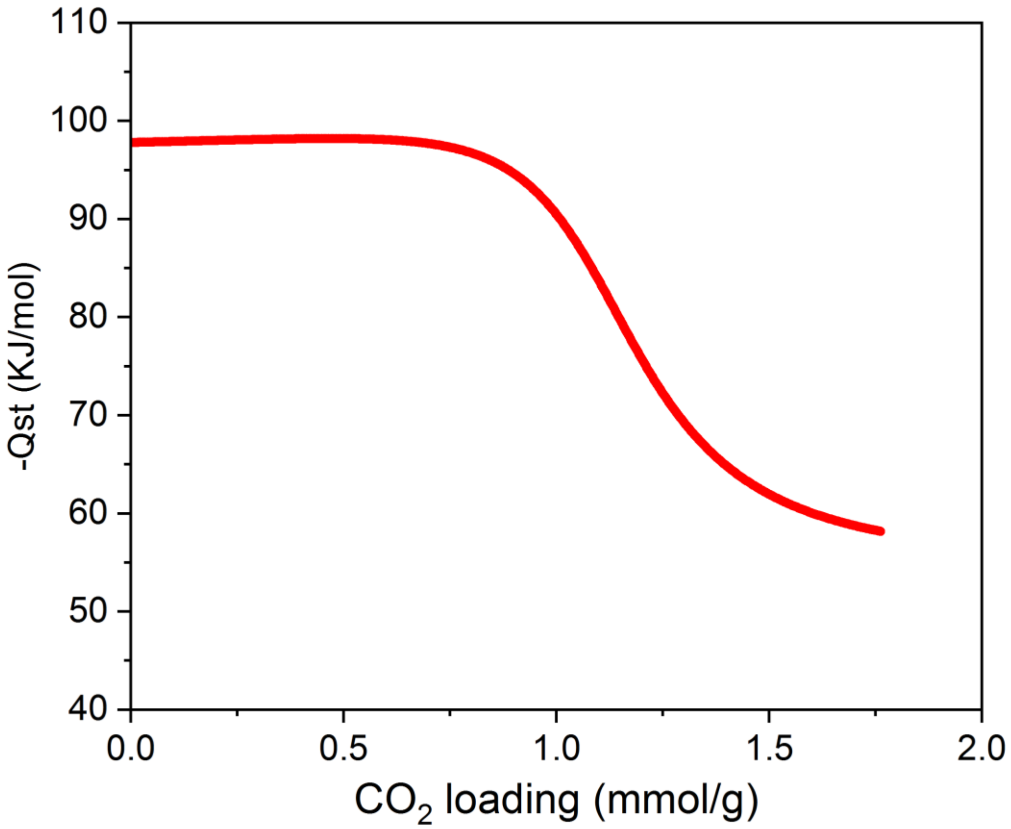

Figure S64. Isosteric heat of CO_2 adsorption of Cr-BDC-TEPA-BDE.

Table S11. Parameters of Cr-BDC-TEPA-BDE composite for the Dual-Site Langmuir fittings with the experimental isotherms.

**Temperature**	$q_{sat,1}$	b_1	$q_{sat,2}$	b_2
313 K	2.146	2.11E-5	1.13	0.00457
333 K	2.05	1.21E-5	1.00	0.00159
353 K	2.15	5.41E-5	1.05	0.000338

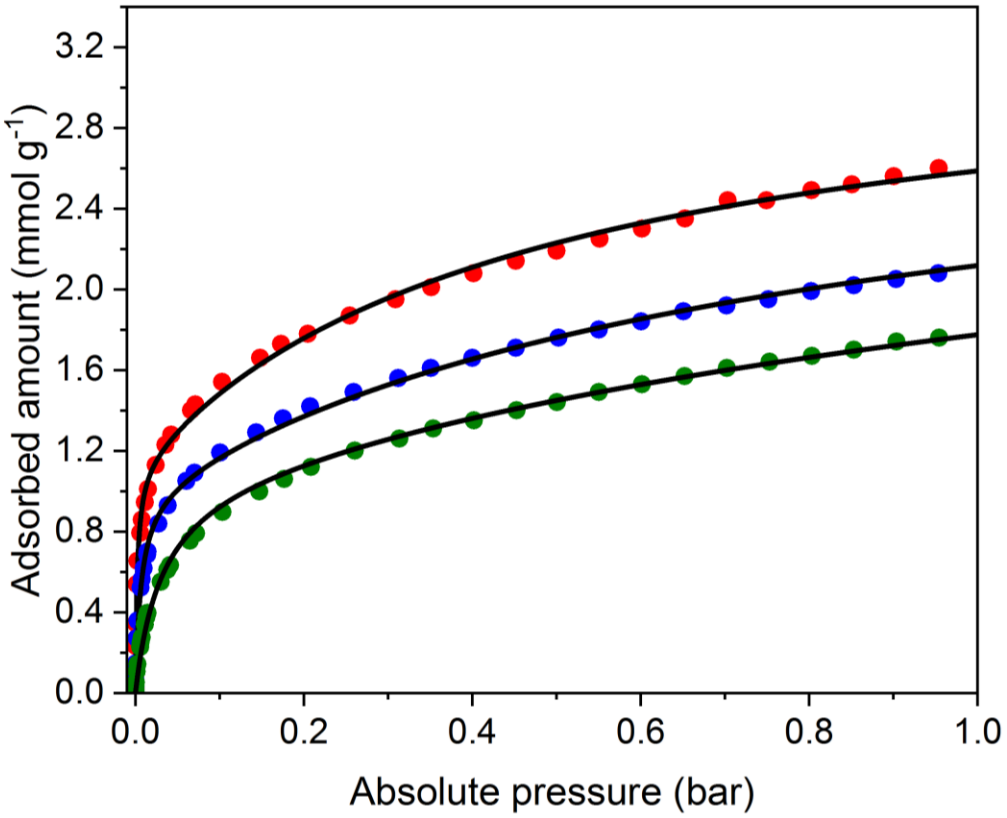

Figure S65. Isotherms of Cr-BDC-TEPA-BDE at 3 different temperatures; 313 K (red dots), 333 K (blue dots), 353 K (green dots), and Dual-Site Langmuir fitting (black line).

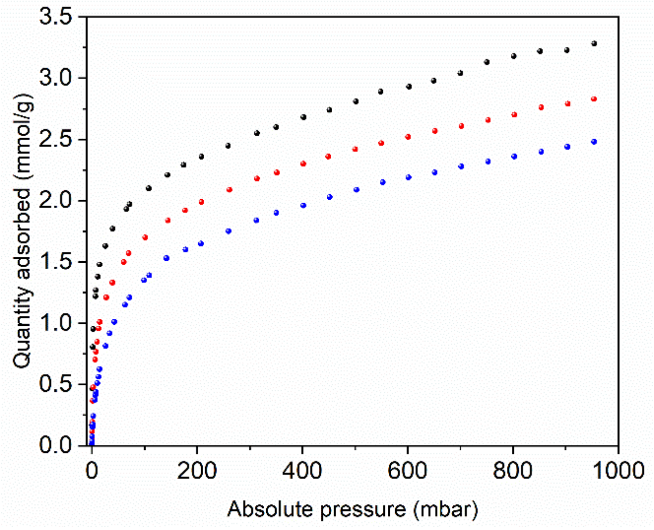

	Temperature (K)	q CO_2 @0.15 bar (mmol/g)
	313	2.24
	333	1.87
	353	1.56

Figure S66. Adsorption isotherm plots at 313 (black), 333 (red) and 353 K (blue) of Cr-BDC-TAEA-BDE and their respective adsorbed amounts at 0.15 bar.

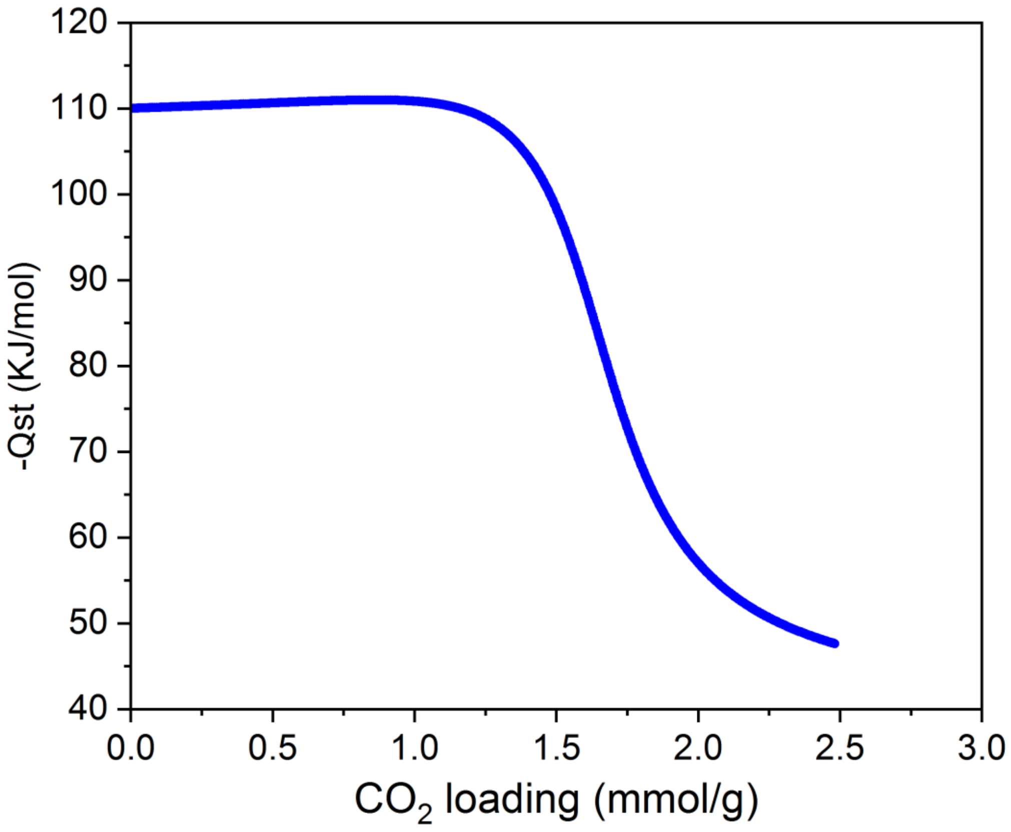

Figure S67. Isosteric heat of CO_2 adsorption of Cr-BDC-TAEA-BDE.

Table S12. Parameters of Cr-BDC-TAEA-BDE composite for the Dual-Site Langmuir fittings with the experimental isotherms.

**Temperature**	$q_{sat,1}$	b_1	$q_{sat,2}$	b_2
313 K	2.4	2.04E-5	1.645	0.0080
333 K	2.4	1.22E-5	1.54	0.00115
353 K	2.6	6.64E-6	1.50	0.000438

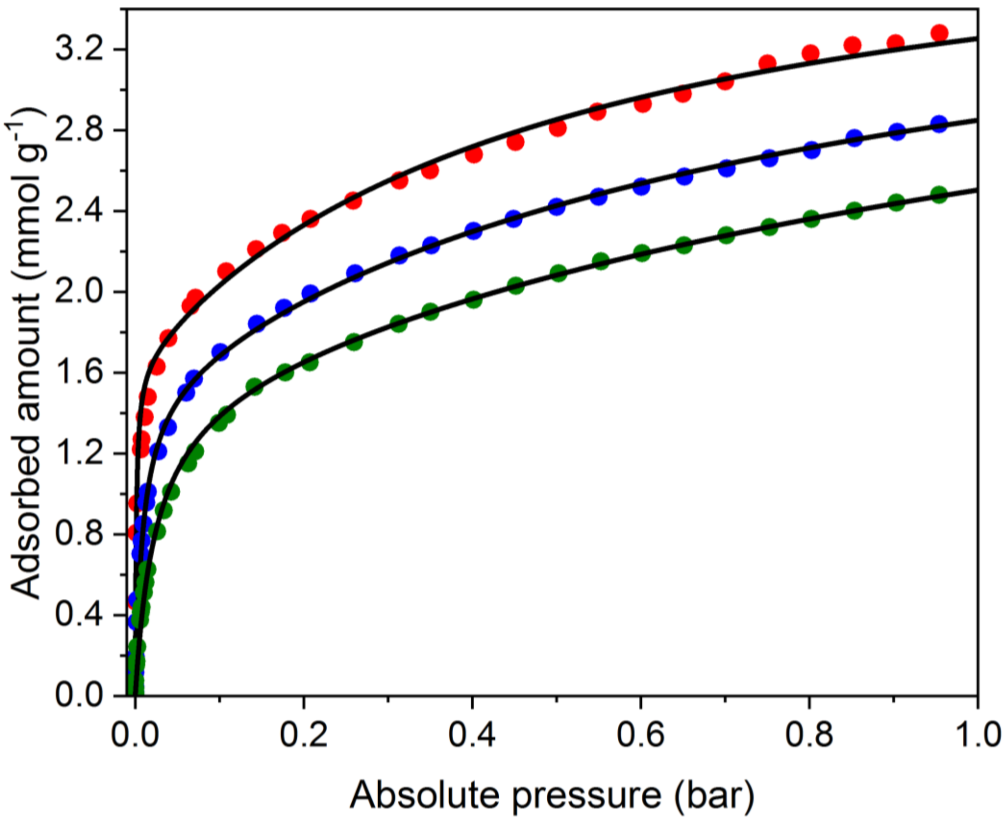

Figure S68. Isotherms of Cr-BDC-TAEA-TMPTE at 3 different temperatures; 313 K (red dots), 333 K (blue dots), 353 K (green dots), and Dual-Site Langmuir fitting (black line).

	Temperature (K)	q CO_2 @0.15 bar (mmol/g)
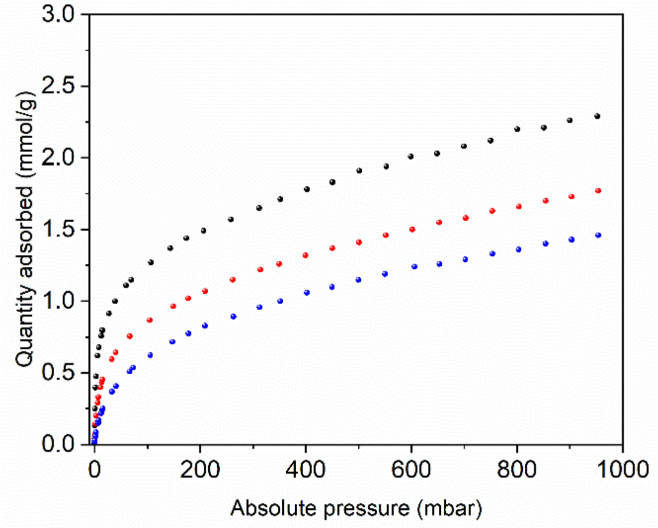	313	1.40
	333	0.97
	353	0.73

Figure S69. Adsorption isotherm plots at 313 (black), 333 (red) and 353 K (blue) of Cr-BDC-TEPA-TMPTE and their respective adsorbed amounts at 0.15 bar.

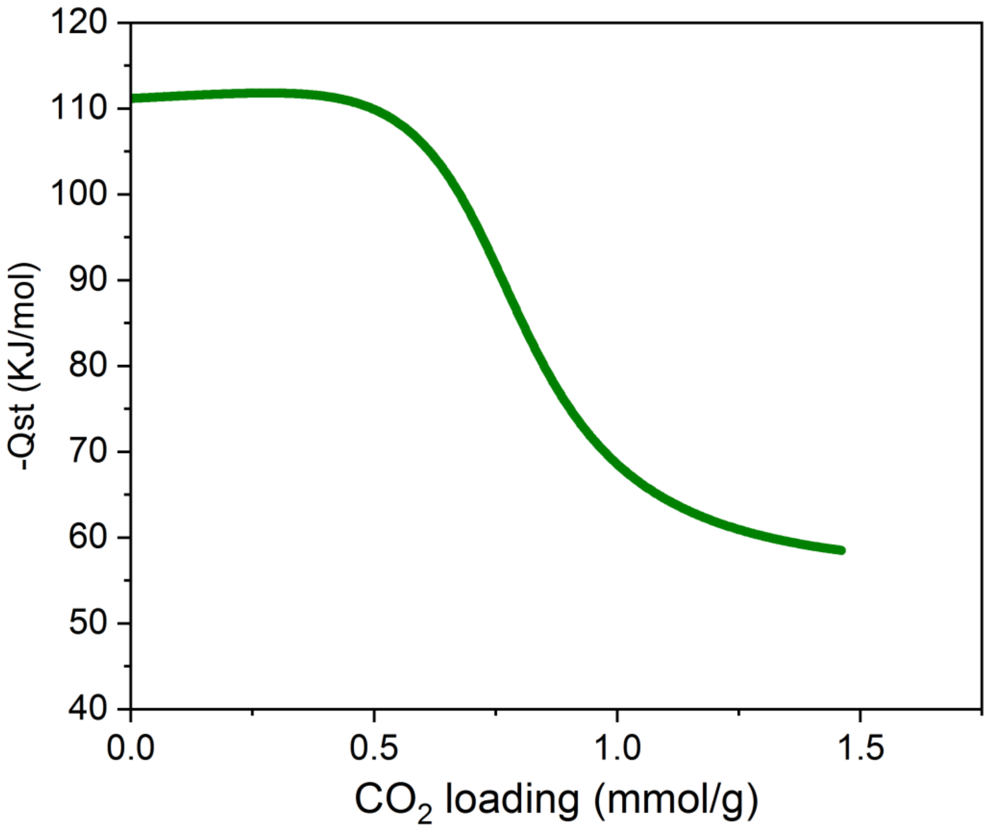

Figure S70. Isosteric heat of CO_2 adsorption of Cr-BDC-TEPA-TMPTE.

Table S13. Parameters of Cr-BDC-TEPA-TMPTE composite for the Dual-Site Langmuir fittings with the experimental isotherms.

**Temperature**	$q_{sat,1}$	b_1	$q_{sat,2}$	b_2
313 K	2.10	2.51E-5	0.77	0.0058
333 K	2.09	1.04E-5	0.72	0.0010
353 K	2.10	6.80E-6	0.65	0.00032

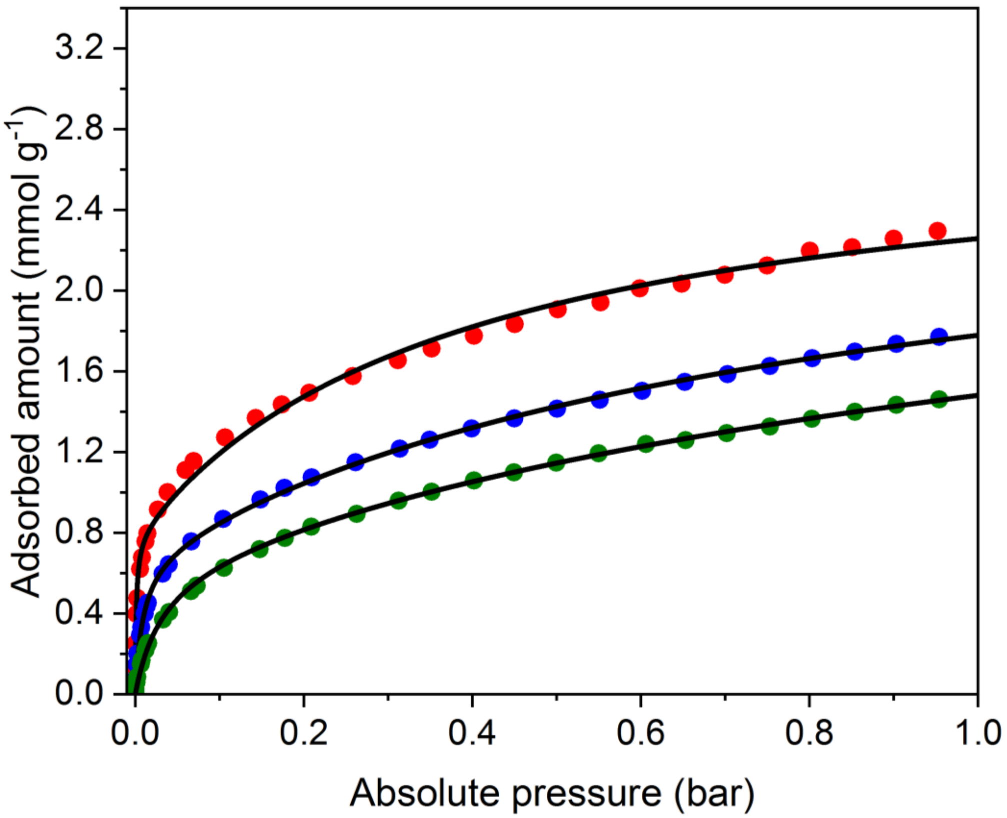

Figure S71. Isotherms of Cr-BDC-TEPA-TMPTE at 3 different temperatures; 313 K (red dots), 333 K (blue dots), 353 K (green dots), and Dual-Site Langmuir fitting (black line).

	**Temperature (K)**	**q CO_2 @0.15 bar (mmol/g)**
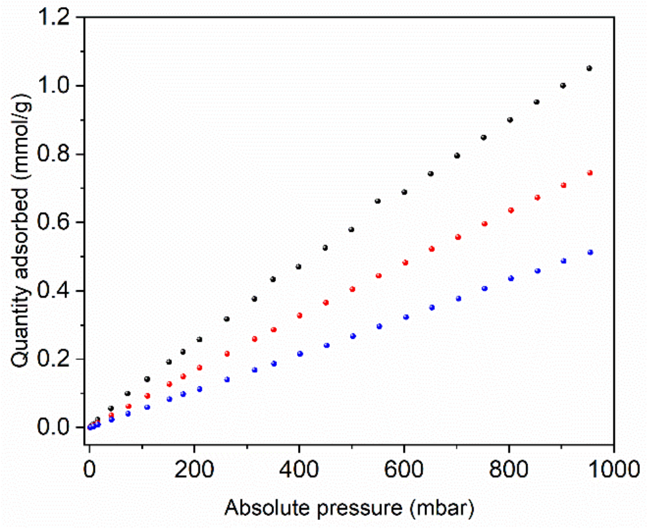	313	0.19
	333	0.13
	353	0.084

Figure S72. Adsorption isotherm plots at 313 (black), 333 (red) and 353 K (blue) of Cr-BDC and their respective adsorbed amounts at 0.15 bar.

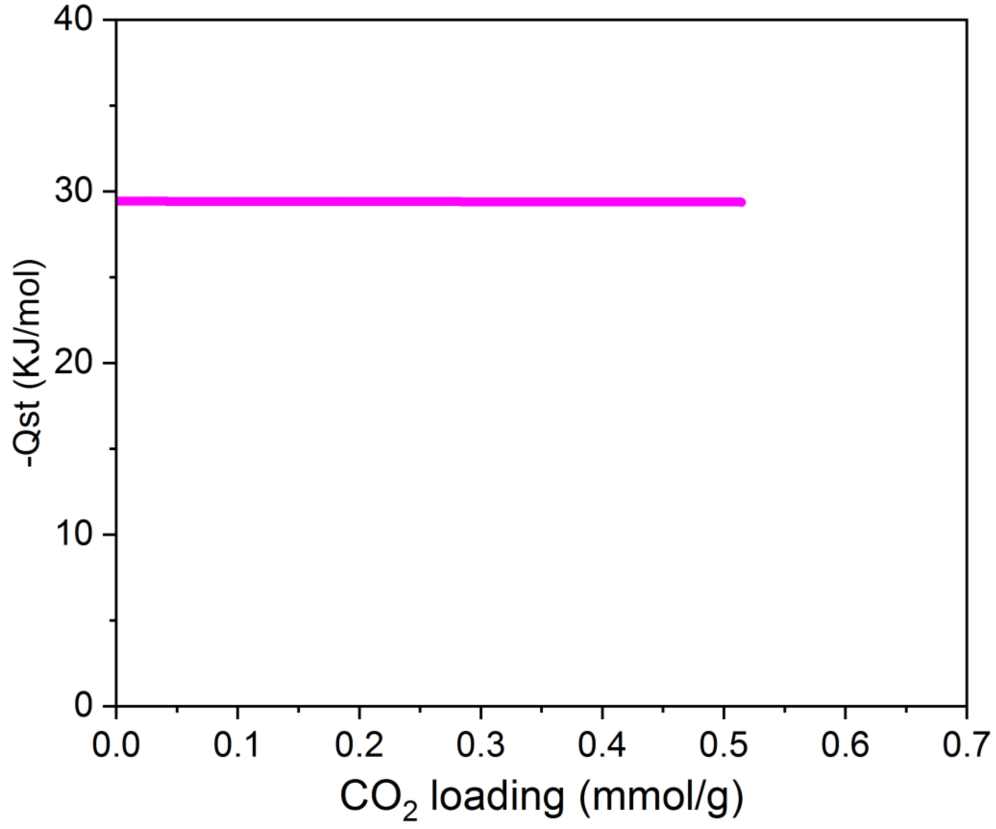

Figure S73. Isosteric heat of CO_2 adsorption of Cr-BDC.

Table S14. Parameters of Cr-BDC composite for the Single-Site Langmuir fittings with the experimental isotherms.

**Temperature**	**$q_{sat,1}$**	**b_1**
313 K	9.0	1.39E-6
333 K	9.2	9.24E-7
353 K	9.2	6.12E-7

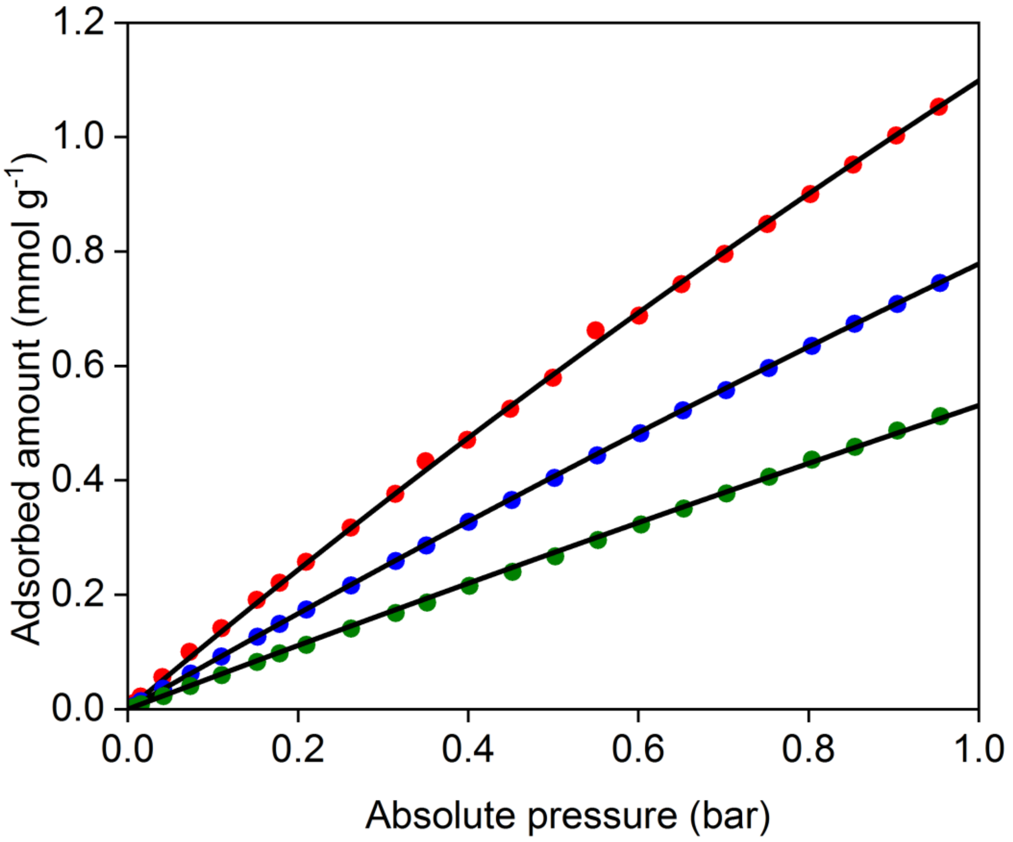

Figure S74. Isotherms of Cr-BDC at 3 different temperatures; 313 K (red dots), 333 K (blue dots), 353 K (green dots), and Single-Site Langmuir fitting (black line).

TSA cycling of Cr-BDC-TAEA-TMPTE using TGA in humid CO_2/N_2 mixture

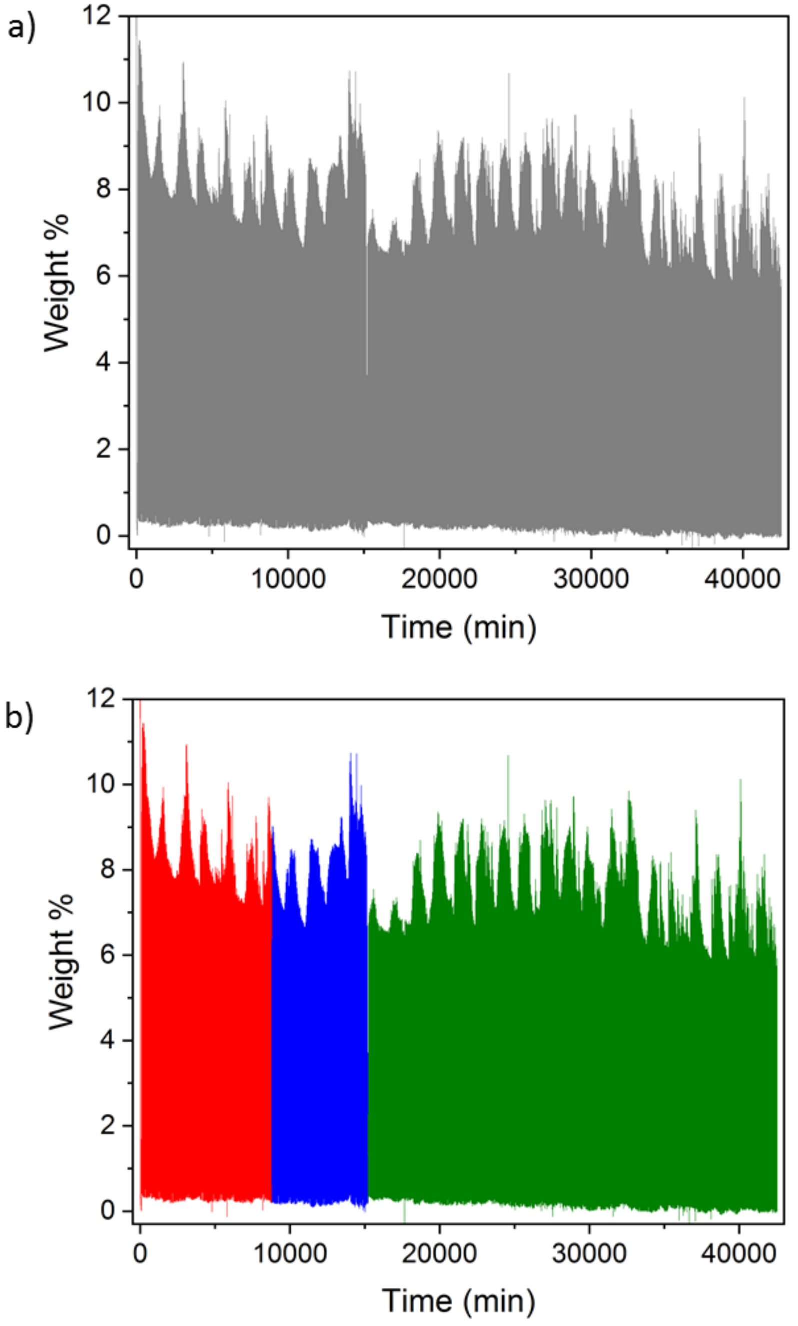

Figure S75. Humid TSA cycles of Cr-BDC-TAEA-TMPTE flowing a 15% CO_2 85 % N_2 gas mixture with an 80 % RH during adsorption at 40 °C, and 100 % CO_2 with an 80 % RH during desorption at 120 °C. a) Plot of the 1000 cycles and b) same plot showing the restart of the experiment with different colors. The same sample was used throughout the 1000 cycles, but the instrument was restarted 2 times.

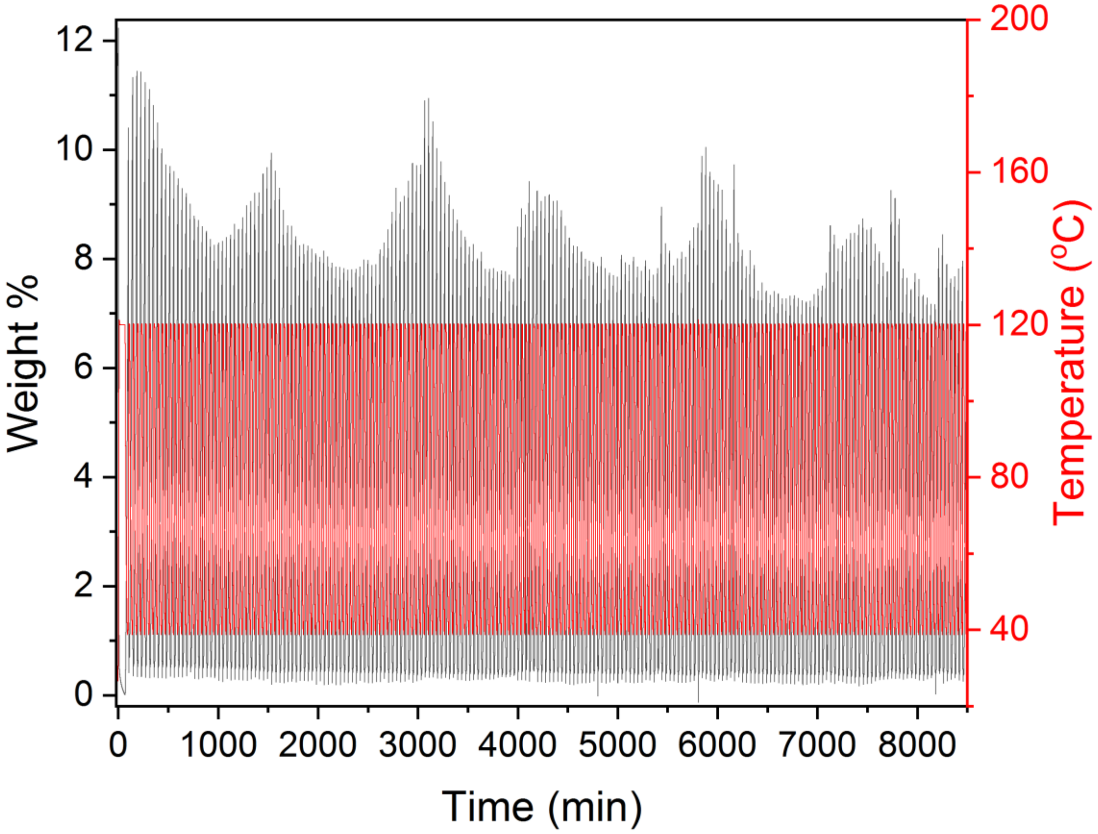

Figure S76. First 200 humid TSA cycles of Cr-BDC-TAEA-TMPTE including the continuous temperature swing plot in red.

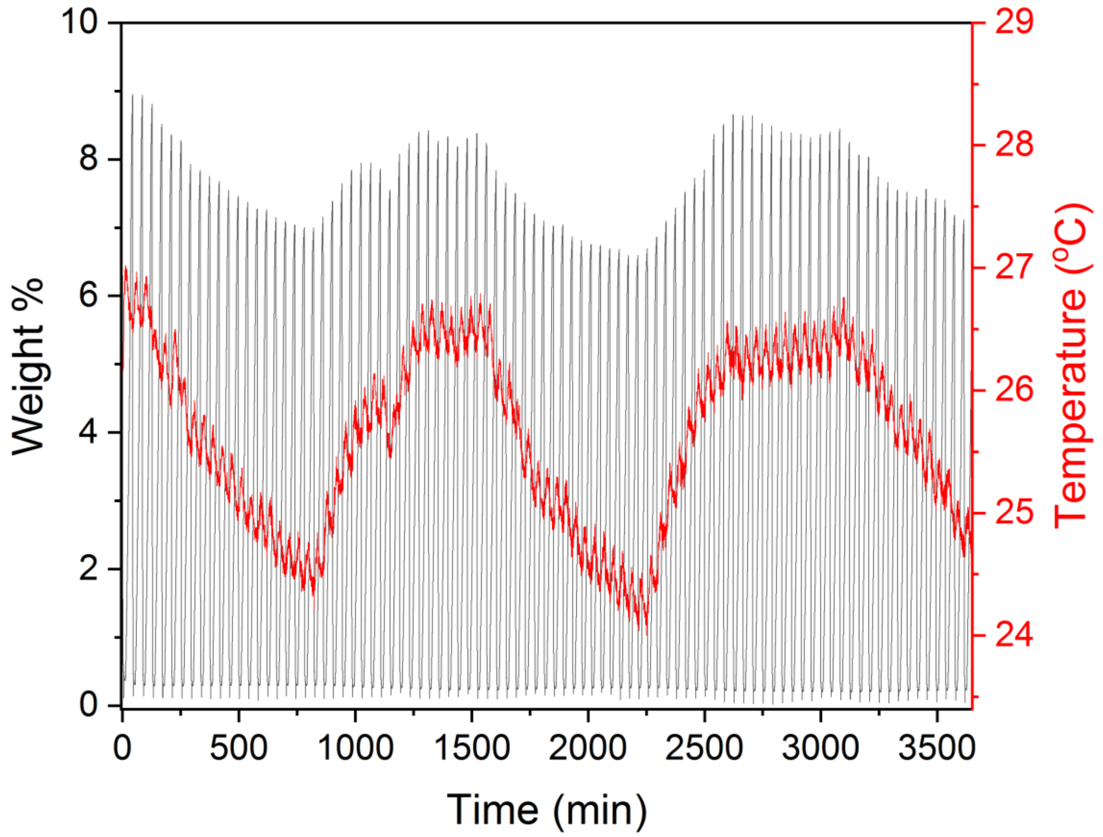

Figure S77. Plot of the TSA cycles and the room temperature during the same experiment. The temperature difference during the test impacts the humidity in the gas stream after the bubbler and, as such, the weight increase of the sample.

CO_2 isotherms for the control, Cr-BDC impregnated with amines using traditional wet impregnation techniques

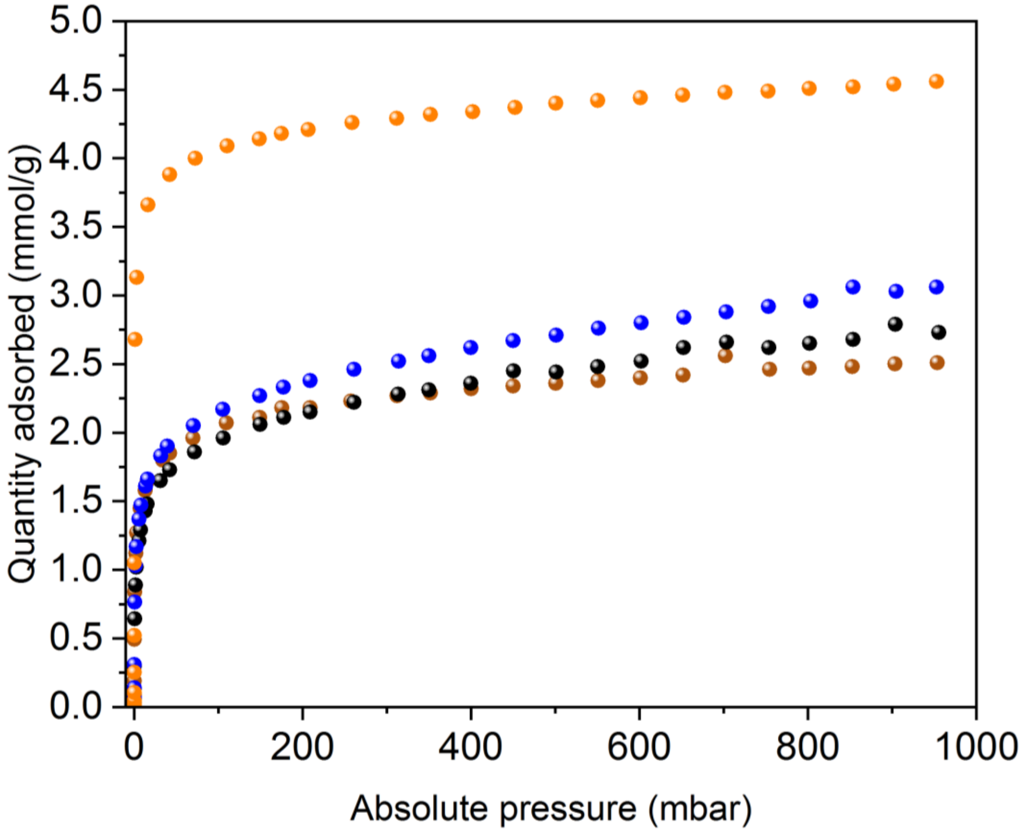

Figure S78. CO_2 adsorption isotherms at 313 K for all TAEA-based materials; Cr-BDC-TAEA-TMPTE (black), Cr-BDC-TAEA-BDE (blue), Cr-BDC-TAEA in cyclohexane (orange) and Cr-BDC-TAEA in methanol (brown). The synthesis of these materials was done at small scales (55 mg of starting Cr-BDC).

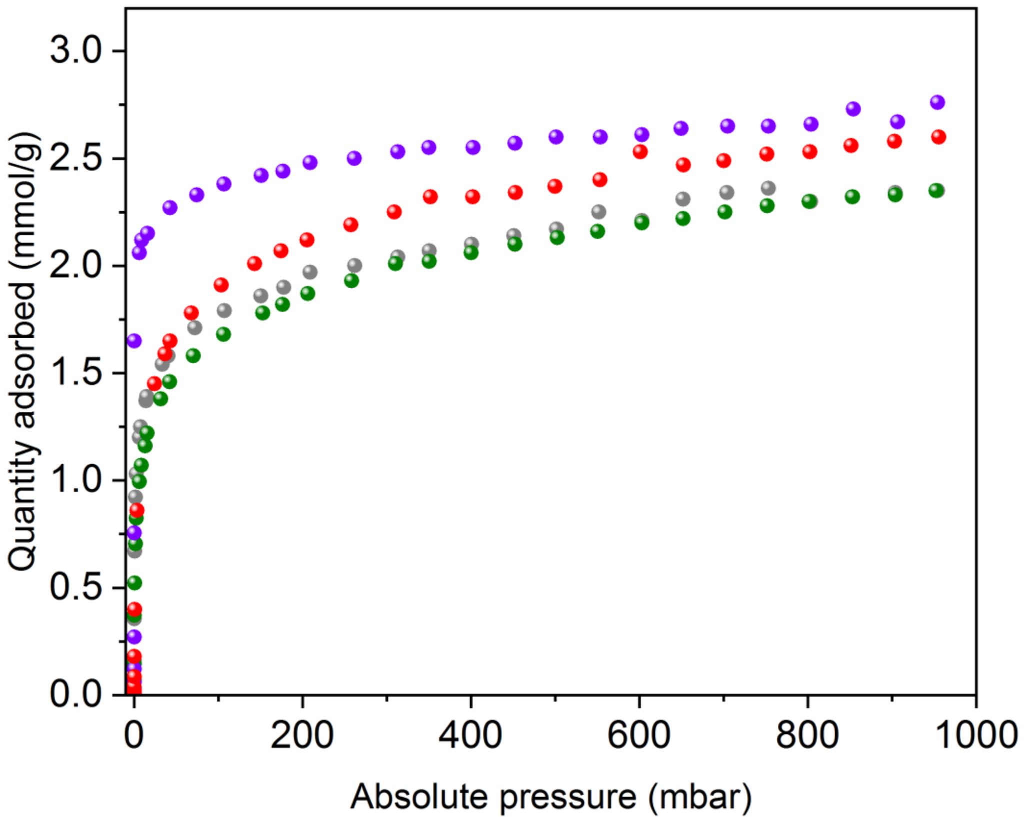

Figure S79. CO_2 adsorption isotherms at 313 K for all TEPA-based materials; Cr-BDC-TEPA-BDE (red), Cr-BDC-TEPA-TMPTE (green), Cr-BDC-TAEA in cyclohexane (purple) and Cr-BDC-TAEA in methanol (grey). The synthesis of these materials was done at small scales (55 mg of starting Cr-BDC).

Table S15. Summary of the CO_2 adsorption capacities at 313 K for the 4 different composites and the control amine-impregnated Cr-BDC at the small scale (55 mg of starting Cr-BDC).

**Composite**	**CO_2 capacity at 313 K and 0.15 bar (mmol/g)**	**CO_2 capacity at 313 K and 1 bar (mmol/g)**
Cr-BDC-TAEA-TMPTE	2.00	2.75
Cr-BDC-TEPA-BDE	1.90	2.61
Cr-BDC-TAEA-BDE	2.22	3.06
Cr-BDC-TEPA-TMPTE	1.71	2.35
Cr-BDC-TAEA in cyclohexane	4.10	4.57
Cr-BDC-TAEA in methanol	2.09	2.51
Cr-BDC-TEPA in cyclohexane	2.40	2.75
Cr-BDC-TEPA in methanol	1.81	2.35

TSA cycling using TGA in pure CO_2

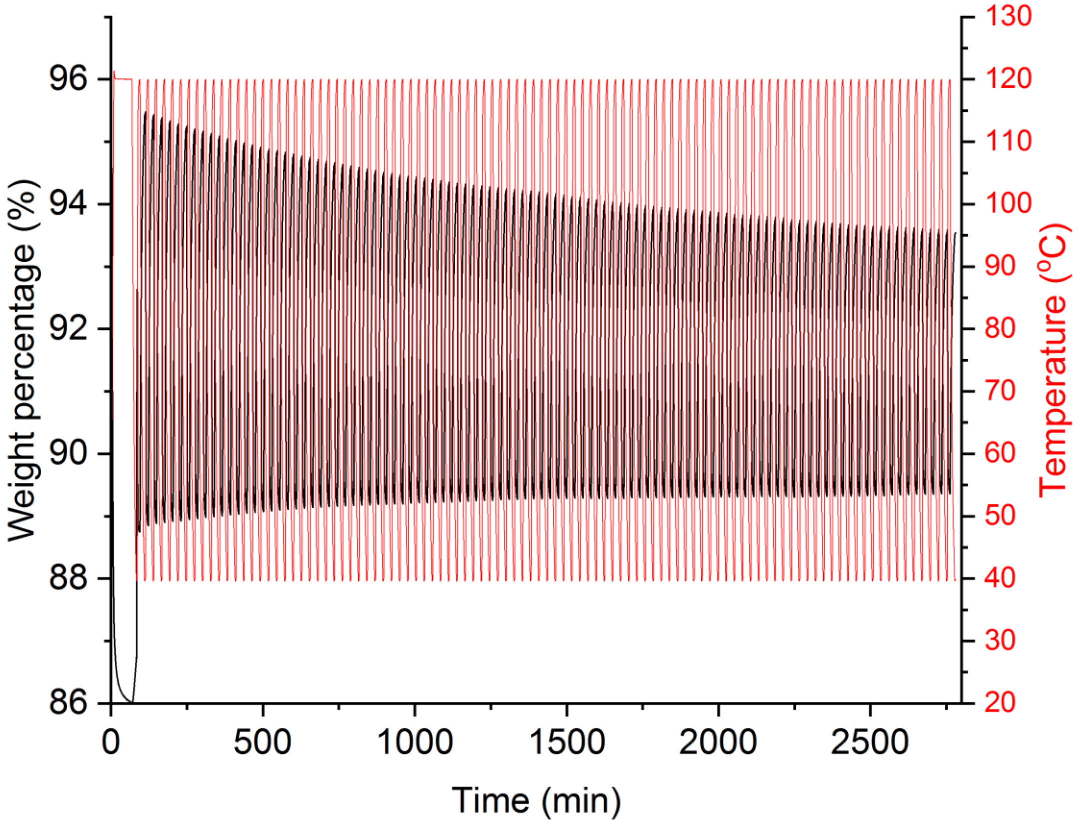

Figure S80. Plot of the TSA cycles of Cr-BDC-TAEA-TMPTE flowing 100 % CO_2 under adsorption at 40 ºC and desorption at 120 ºC. The black line represents the weight change and the red line the temperature.

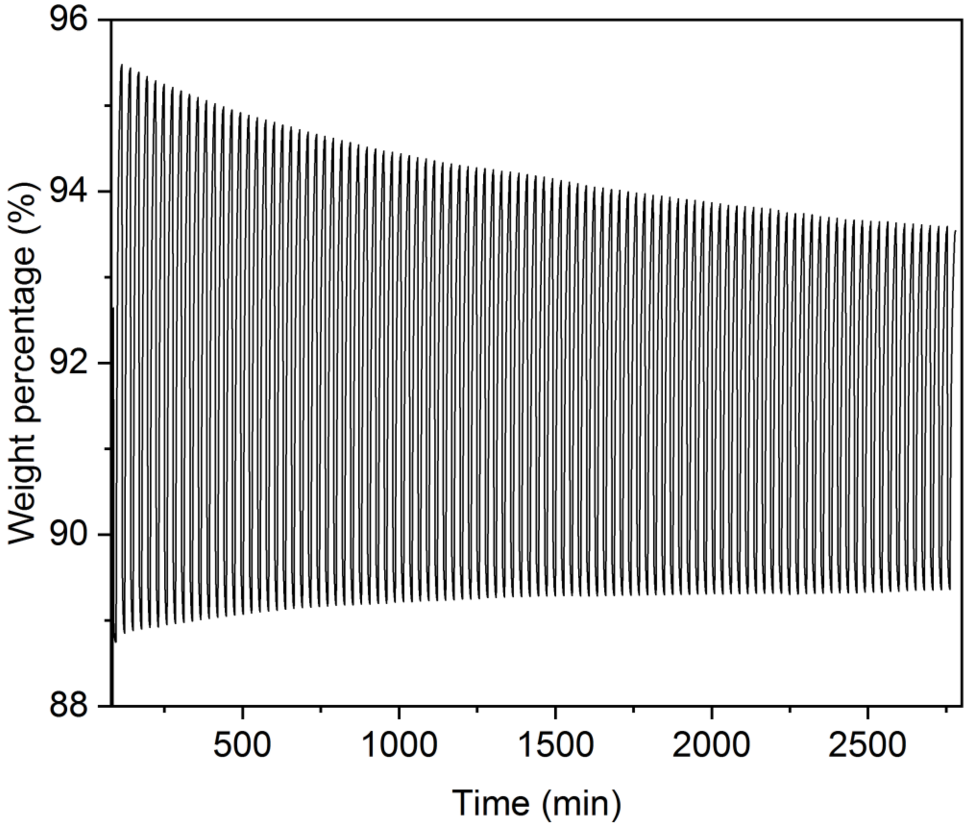

Figure S81. Enlarged plot of the weight change during the TSA cycles of Cr-BDC-TAEA-TMPTE flowing 100 % CO_2 under adsorption at 40 ºC and desorption at 120 ºC.

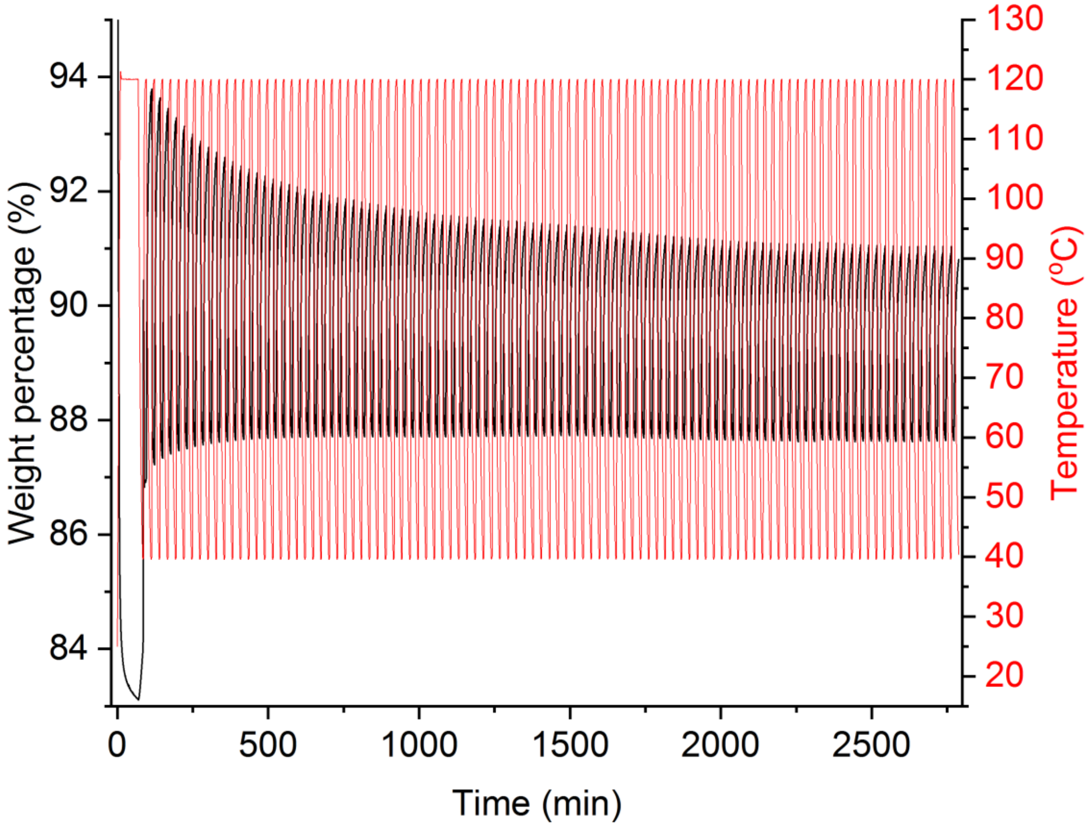

Figure S82. Plot of the TSA cycles of Cr-BDC-TEPA-BDE flowing 100 % CO_2 under adsorption at 40 ºC and desorption at 120 ºC. The black line represents the weight change and the red line the temperature.

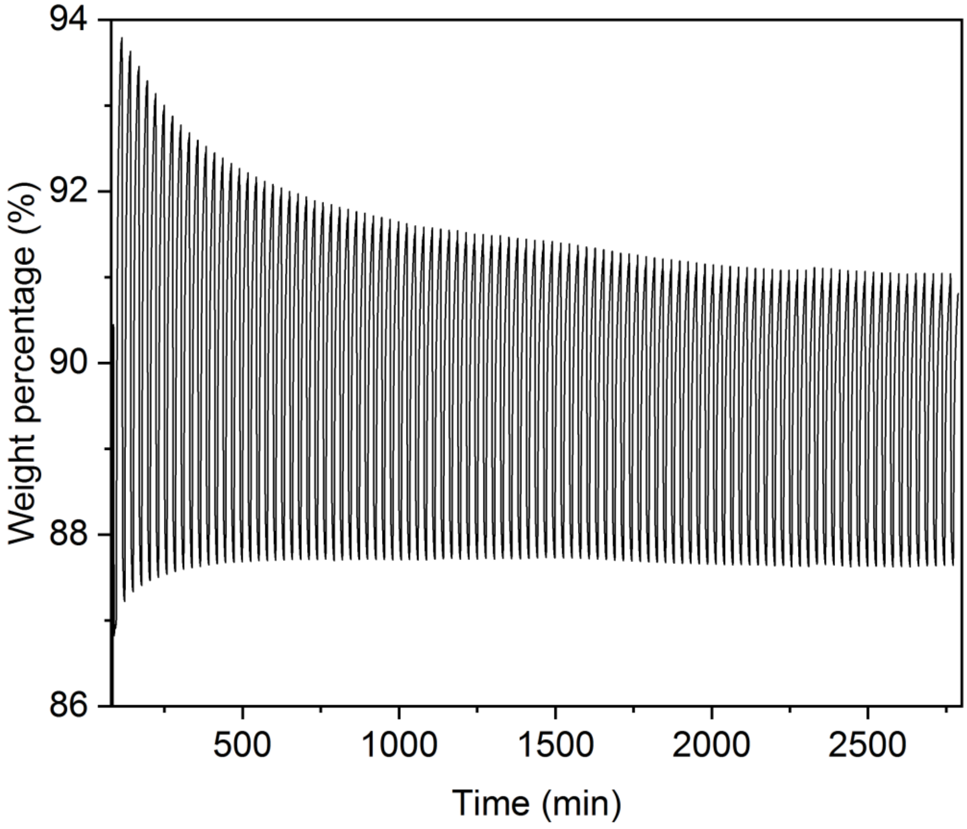

Figure S83. Enlarged plot of the weight change during the TSA cycles of Cr-BDC-TEPA-BDE flowing 100 % CO_2 under adsorption at 40 ^{o}C and desorption at 120 ^{o}C.

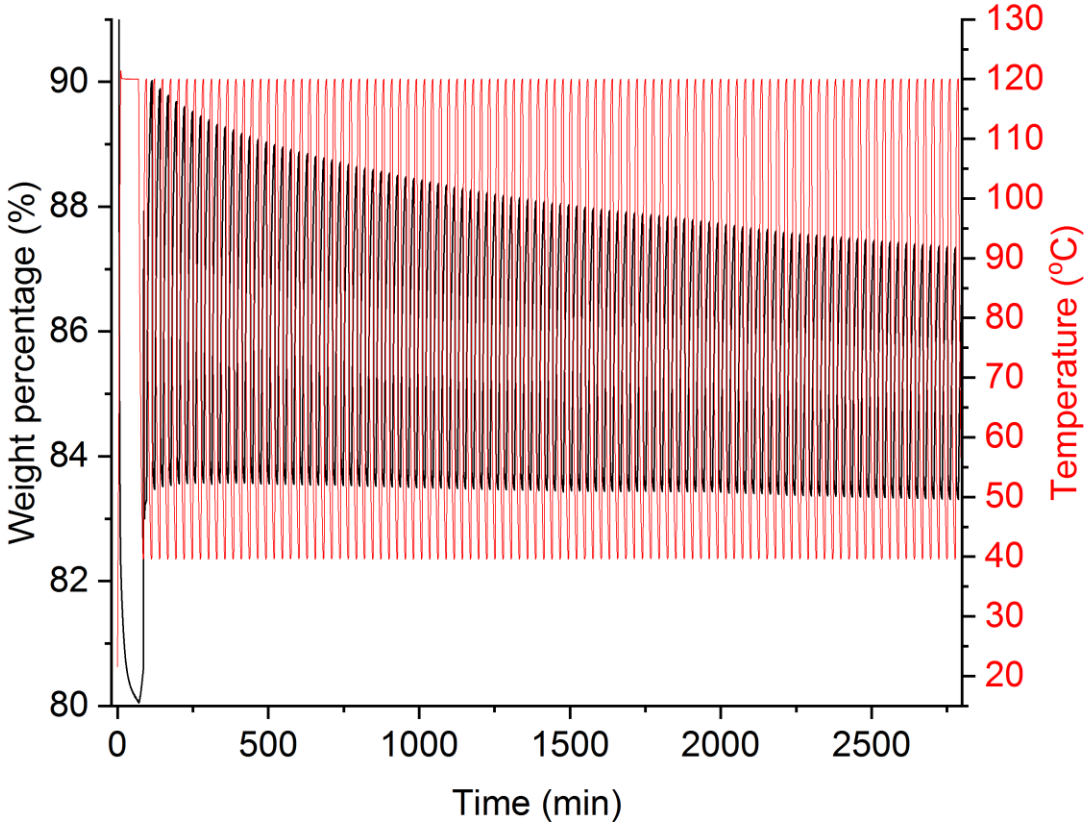

Figure S84. Plot of the TSA cycles of Cr-BDC-TAEA-BDE flowing 100 % CO_2 under adsorption at 40 ºC and desorption at 120 ºC. The black line represents the weight change and the red line the temperature.

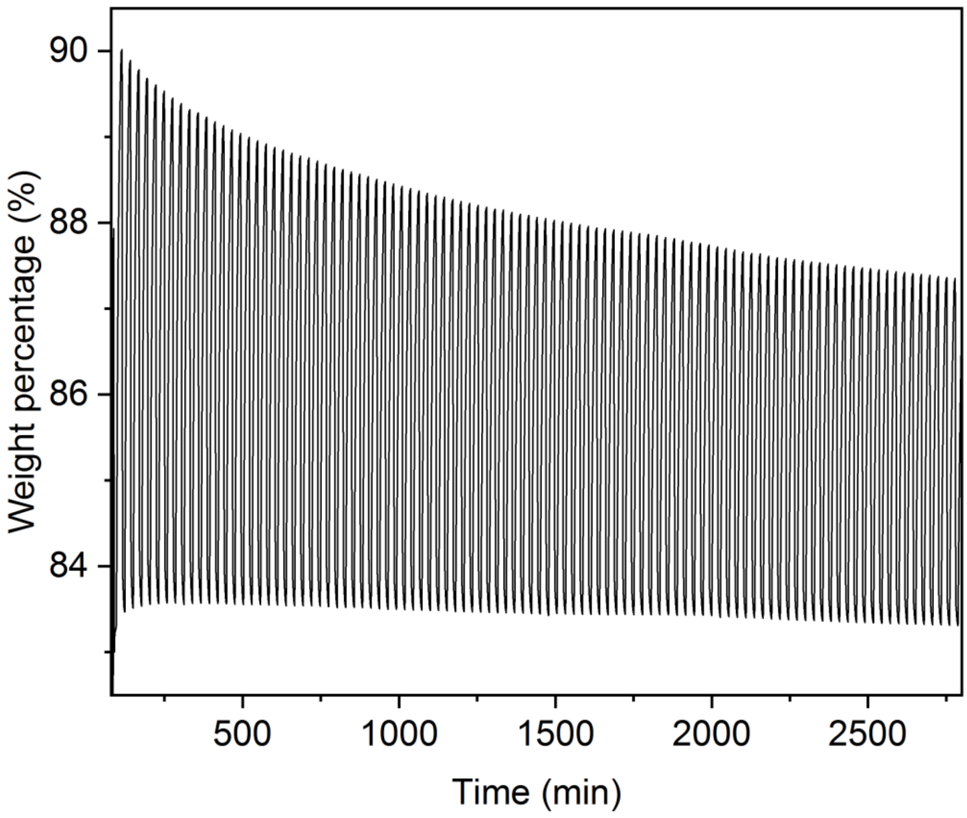

Figure S85. Enlarged plot of the weight change during the TSA cycles of Cr-BDC-TAEA-BDE flowing 100 % CO_2 under adsorption at 40 °C and desorption at 120 °C.

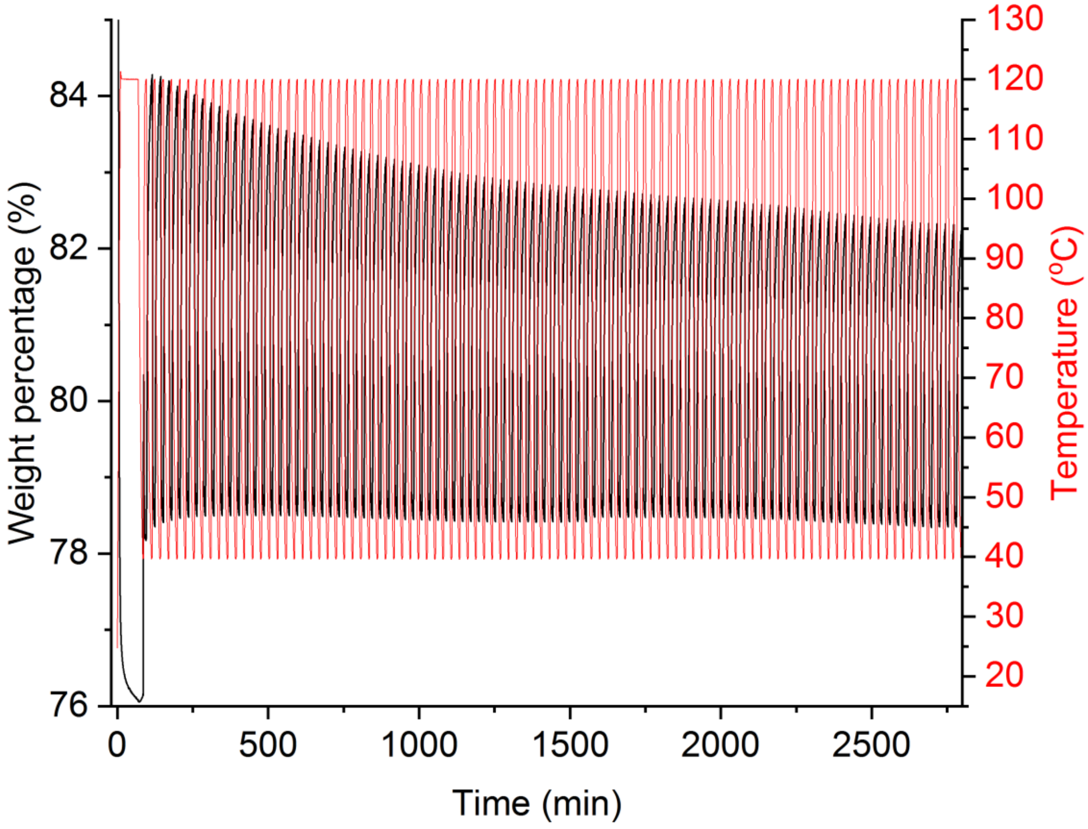

Figure S86. Plot of the TSA cycles of Cr-BDC-TEPA-TMPTE flowing 100 % CO_2 under adsorption at 40 °C and desorption at 120 °C. The black line represents the weight change and the red line the temperature.

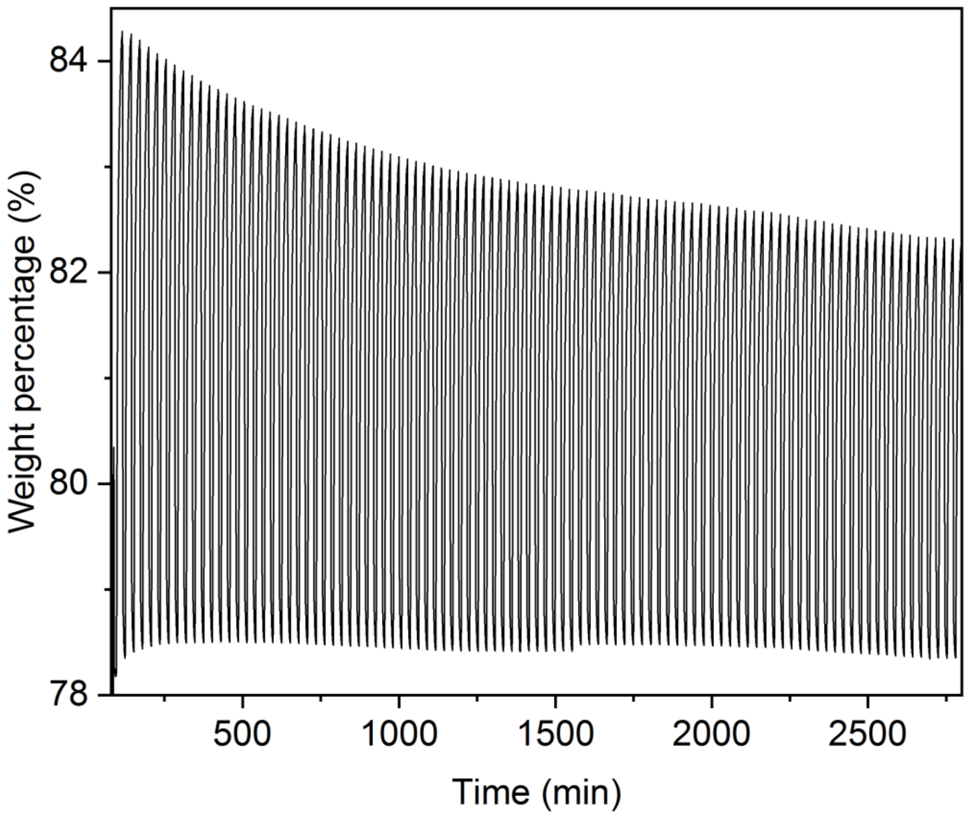

Figure S87. Enlarged plot of the weight change during the TSA cycles of Cr-BDC-TEPA-TMPTE flowing 100 % CO_2 under adsorption at 40 ºC and desorption at 120 ºC.

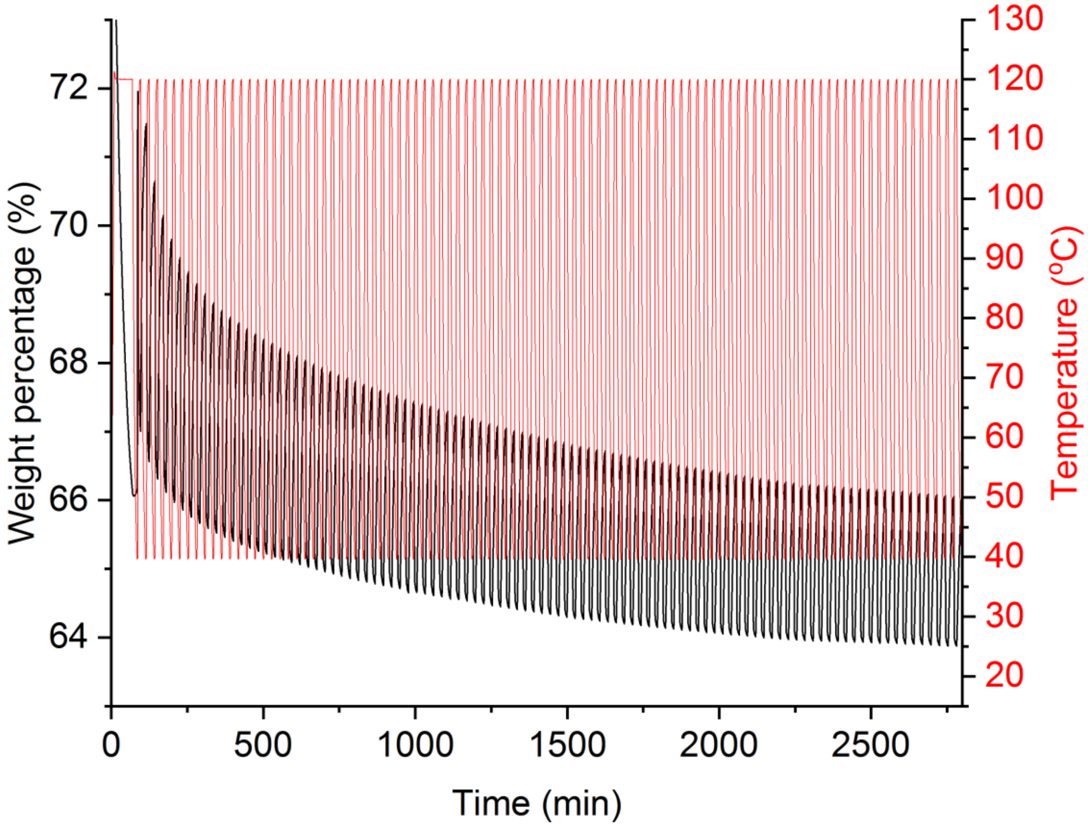

Figure S88. Plot of the TSA cycles of Cr-BDC-TAEA impregnated with cyclohexane flowing 100 % CO_2 under adsorption at 40 ºC and desorption at 120 ºC. The black line represents the weight change and the red line the temperature.

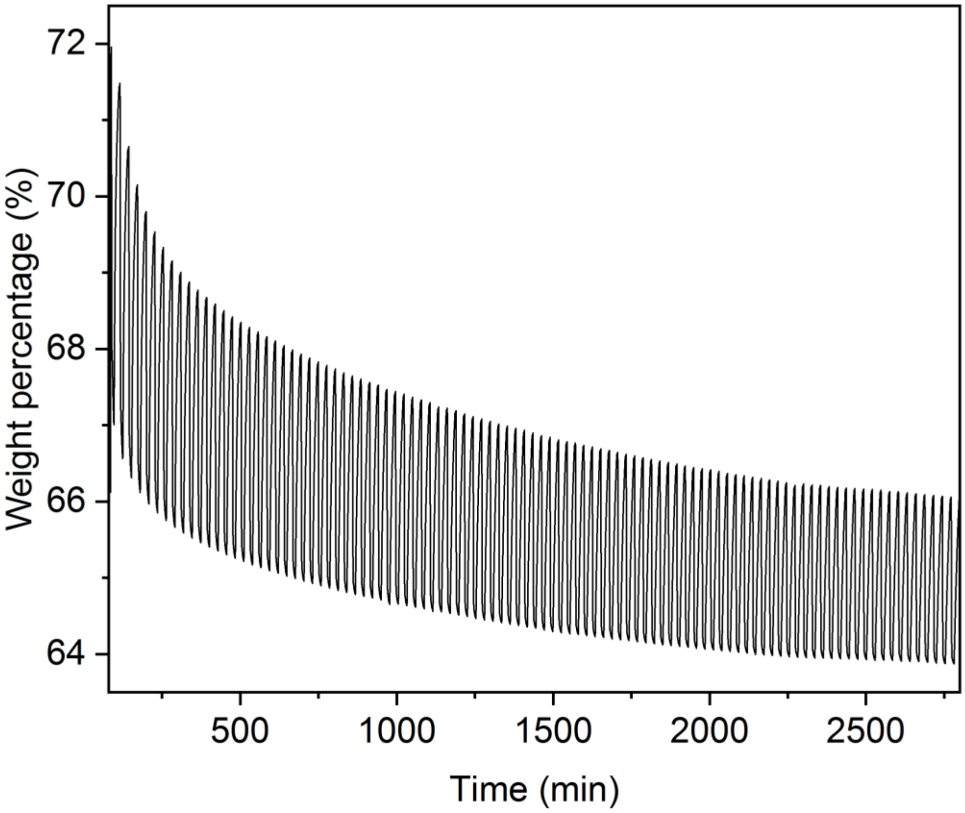

Figure S89. Enlarged plot of the weight change during the TSA cycles of Cr-BDC-TAEA impregnated with cyclohexane flowing 100 % CO_2 under adsorption at 40 ^{o}C and desorption at 120 ^{o}C.

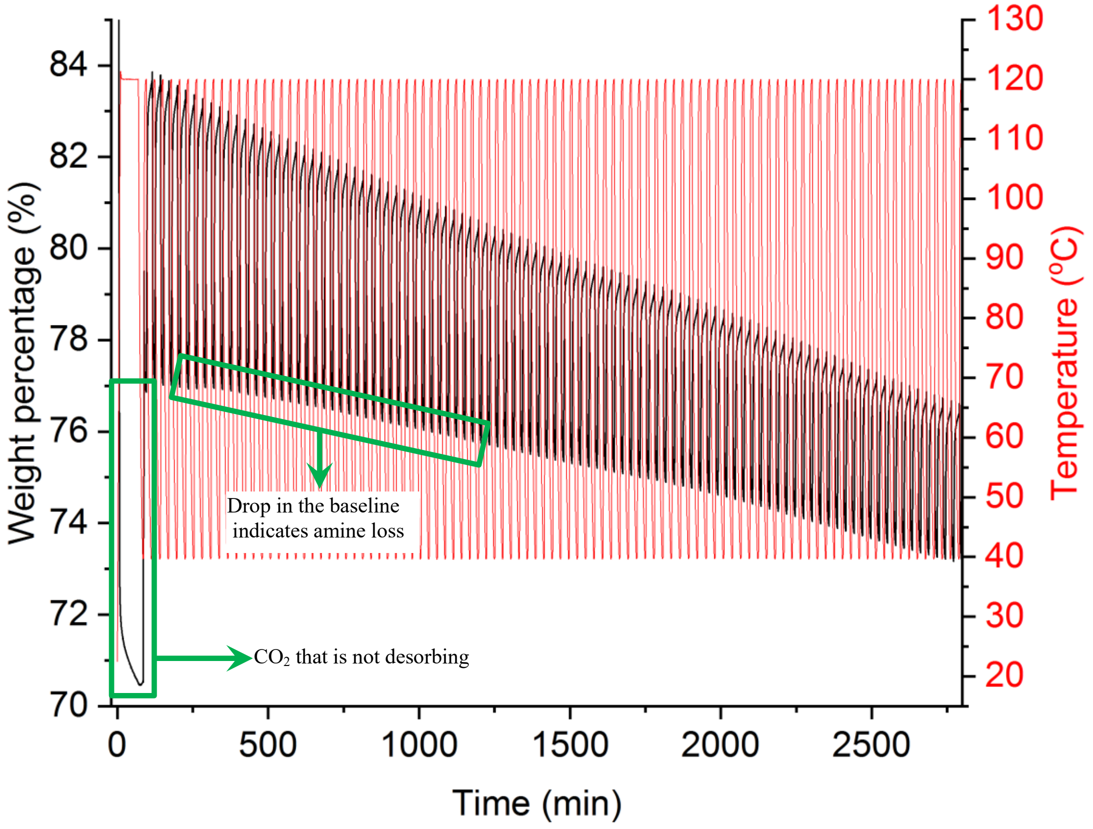

Figure S90. Enlarged plot of the weight change during the TSA cycles of Cr-BDC-TEPA impregnated with cyclohexane flowing 100 % CO_2 under adsorption at 40 °C and desorption at 120 °C. The black line represents the weight change and the red line the temperature.

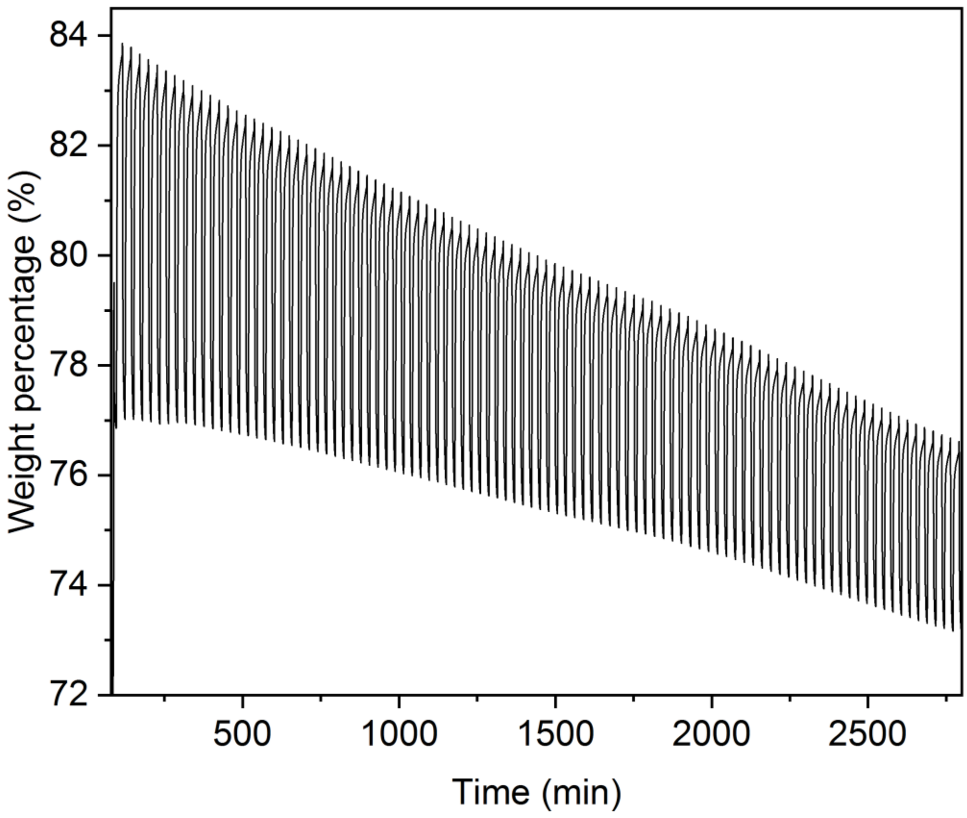

Figure S91. Enlarged plot of the weight change during the TSA cycles of Cr-BDC-TEPA impregnated with cyclohexane flowing 100 % CO_2 under adsorption at 40 °C and desorption at 120 °C.

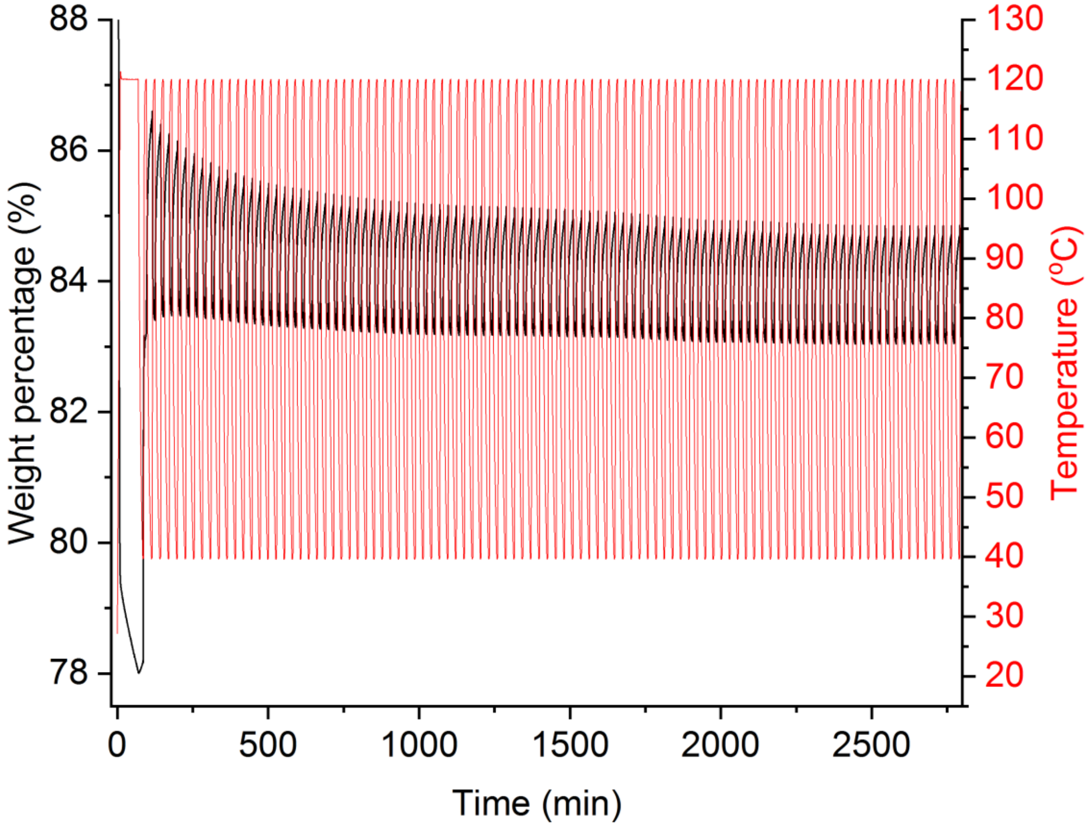

Figure S92. Plot of the TSA cycles of Cr-BDC-TAEA impregnated with methanol flowing 100 % CO_2 under adsorption at 40 °C and desorption at 120 °C. The black line represents the weight change and the red line the temperature.

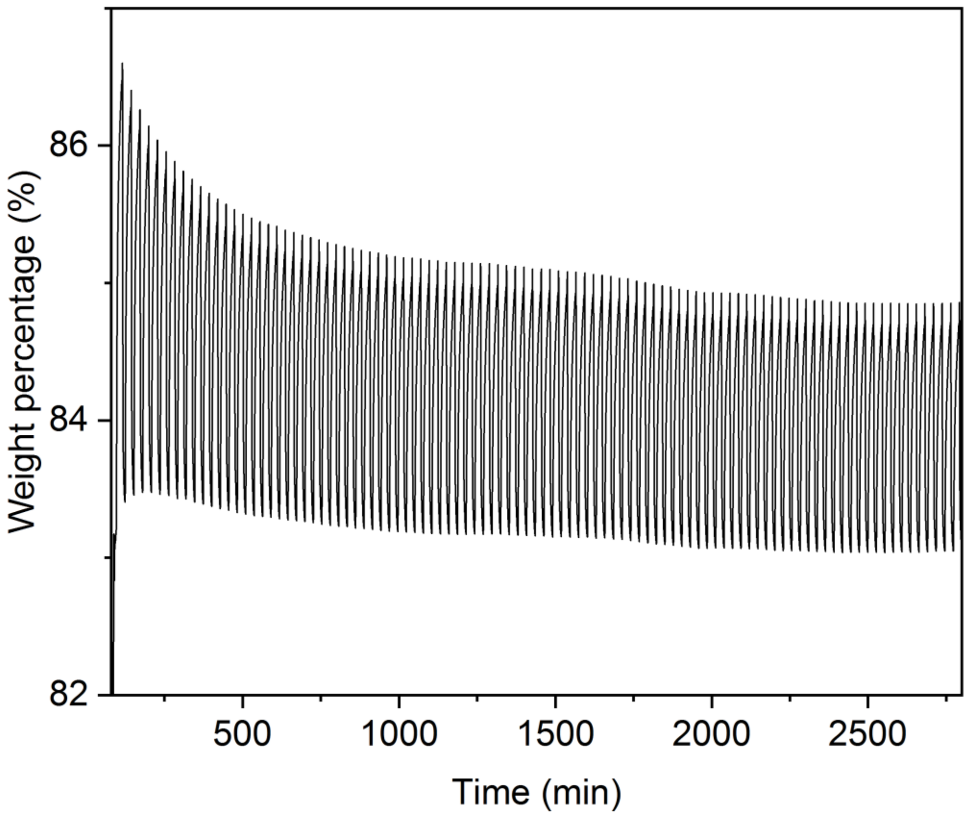

Figure S93. Enlarged plot of the weight change during the TSA cycles of Cr-BDC-TAEA impregnated with methanol flowing 100 % CO_2 under adsorption at 40 ^{o}C and desorption at 120 ^{o}C.

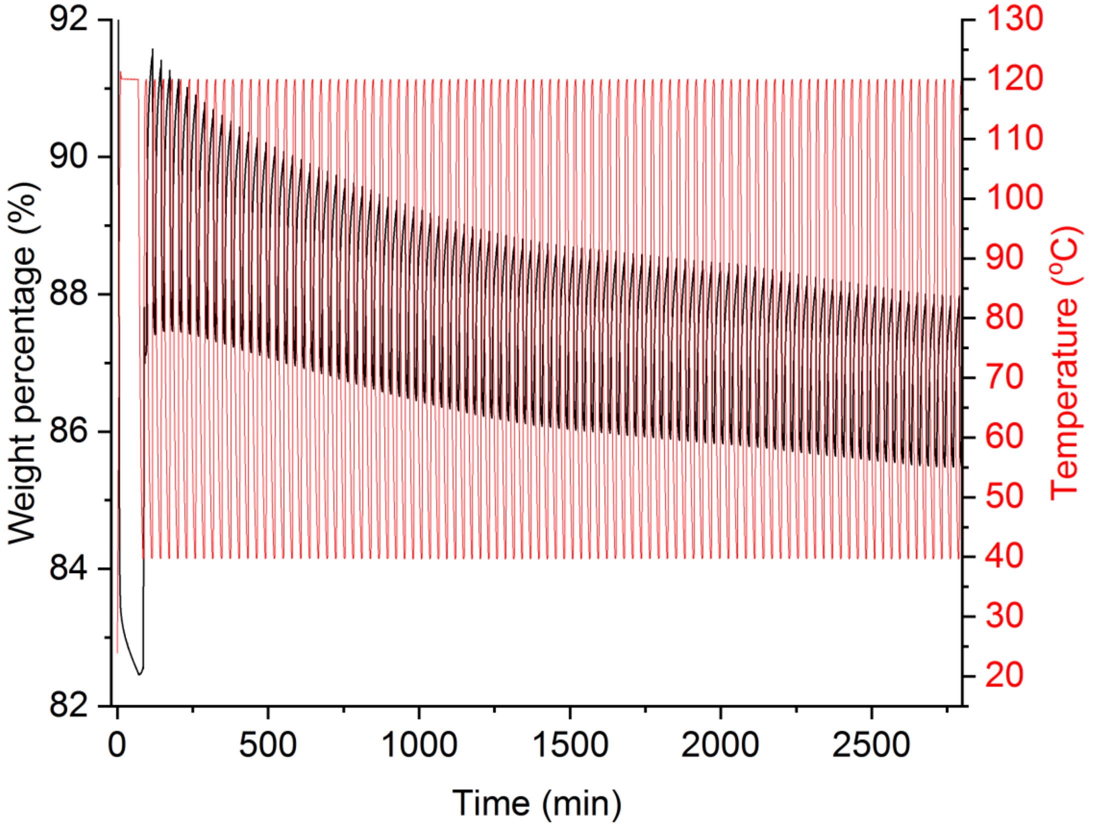

Figure S94. Enlarged plot of the weight change during the TSA cycles of Cr-BDC-TEPA impregnated with methanol flowing 100 % CO_2 under adsorption at 40 ºC and desorption at 120 ºC. The black line represents the weight change and the red line the temperature.

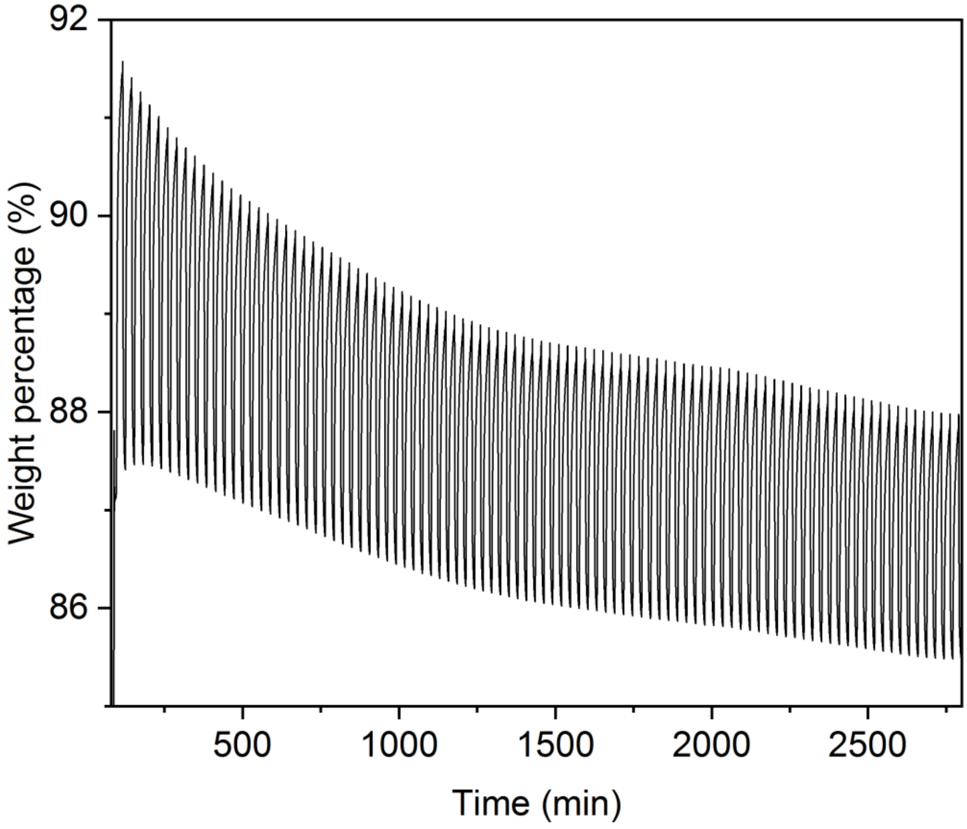

Figure S95. Enlarged plot of the weight change during the TSA cycles of Cr-BDC-TEPA impregnated with methanol flowing 100 % CO_2 under adsorption at 40 ^oC and desorption at 120 ^oC.

Accelerated CO_2 aging

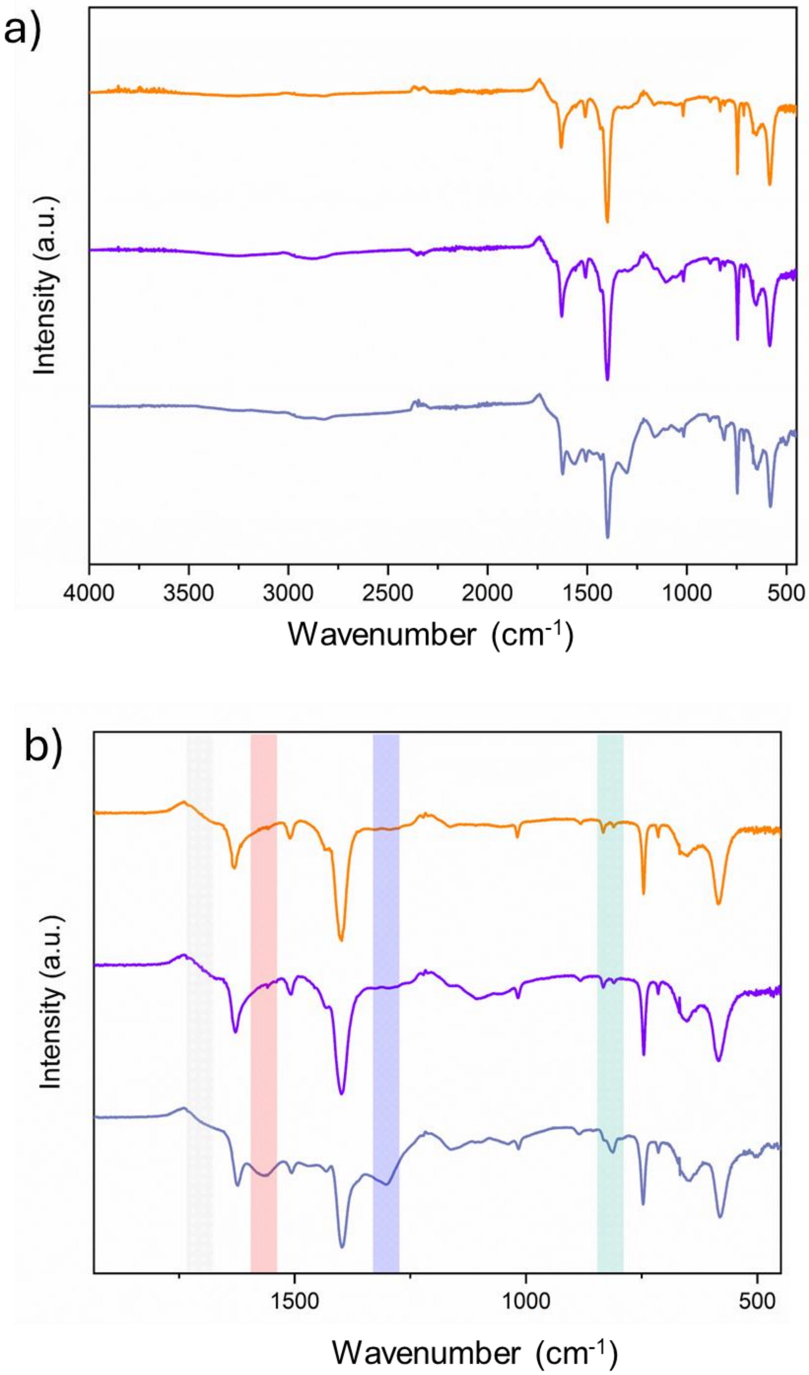

Figure S96. a) DRIFTS spectra of the Cr-BDC-TAEA-BDE (orange), Cr-BDC-TAEA-TMPTE (purple), and Cr-BDC-TAEA CH (blue) after accelerated CO_2 ageing. b) Zoomed in spectra that highlights the new peaks arising from amine degradation in the Cr-BDC-TAEA CH (blue) sample.

Q_{st} for composites with tuned polymer composition

Table S16. Summary of experimental conditions to synthesize new composites with different amine-epoxide ratios, CO_2 capacities and heats of adsorption. The highlights indicate the parameter that was changed in the synthesis.

Sample	Cr-BDC amount	TAEA amount (amine)	BDE amount (epoxide)	Organics content (%)	CO_2 capacity 313K / 0.15 bar (mmol/g)	Heat of adsorption (kJ/mol)
Cr-BDC-TAEA-BDE Original	550 mg	4.05 mL	1.07 mL	42	2.23	-110
Cr-BDC -TAEA-BDE Dilute	**1100 mg**	4.05 mL	1.07 mL	19.9	1.33	-55
Cr-BDC -TAEA-BDE +50% epoxide	550 mg	4.05 mL	**1.58 mL**	46.5	1.14	-62
Cr-BDC -TAEA-BDE 75% amine	550 mg	**3.04 mL**	1.07 mL	31.8	1.85	-79

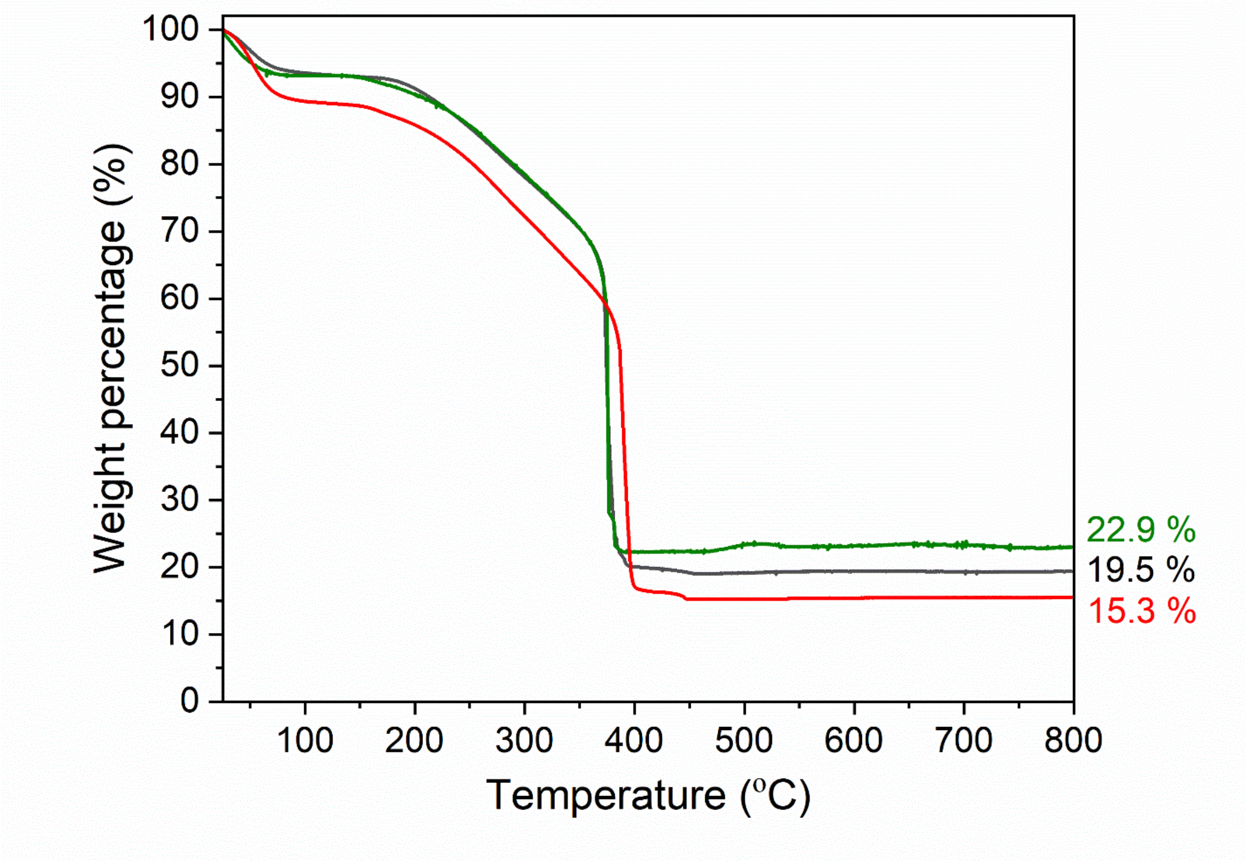

Figure S97. TGA plot of the 3 composites assessed, Cr-BDC-TAEA-BDE (75% amine) (black), Cr-BDC-TAEA-BDE (+50% epoxide) (red), and Cr-BDC-TAEA-BDE (dilute) (green). Organics content calculated from Equation 2 and summarized in Table S16.

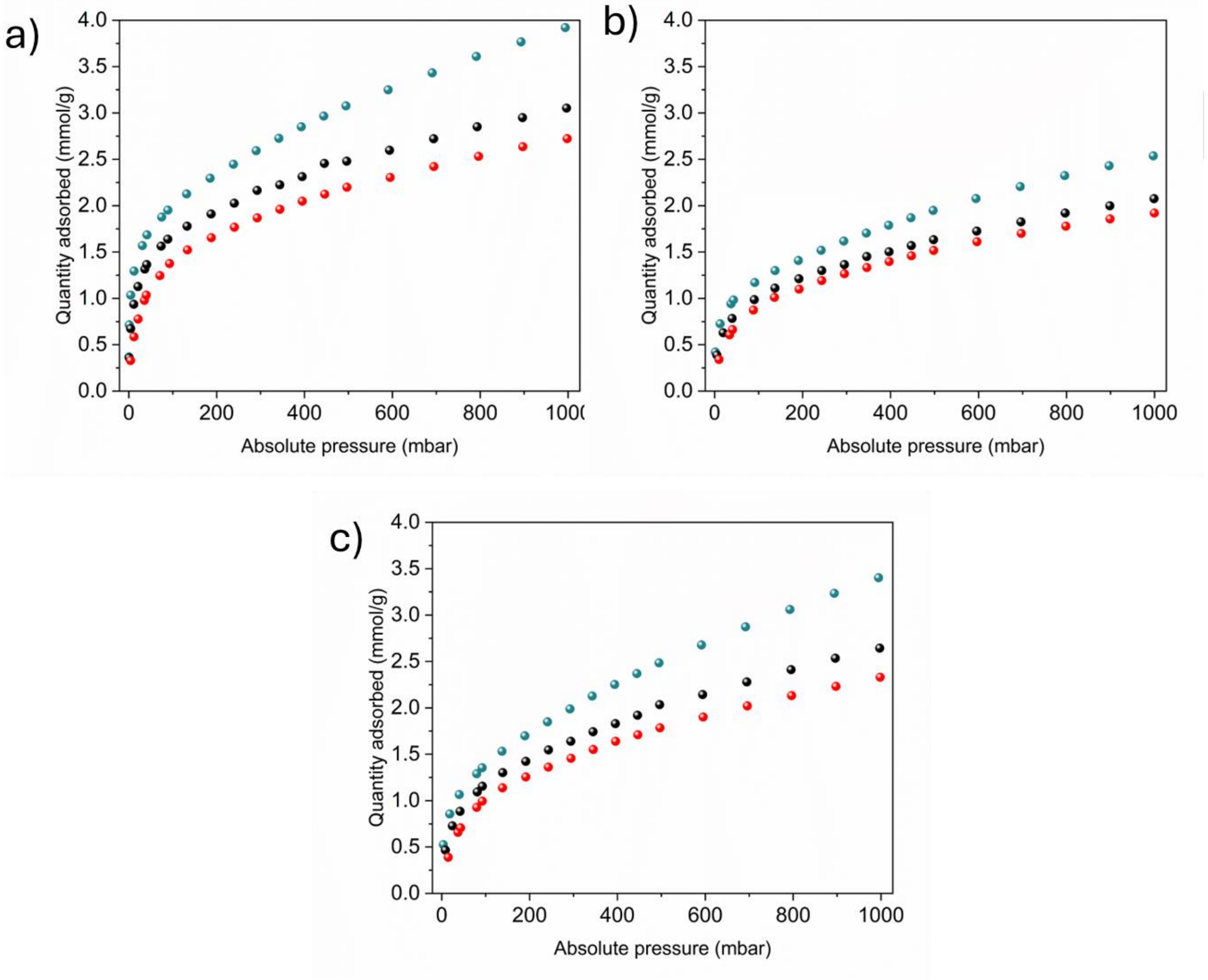

Figure S98. CO_2 isotherms at 3 different temperatures (293K – blue, 313K – black, and 333K – red) for a) Cr-BDC-TAEA-BDE (75% amine), b) Cr-BDC-TAEA-BDE (+50% epoxide), and c) Cr-BDC-TAEA-BDE (dilute).

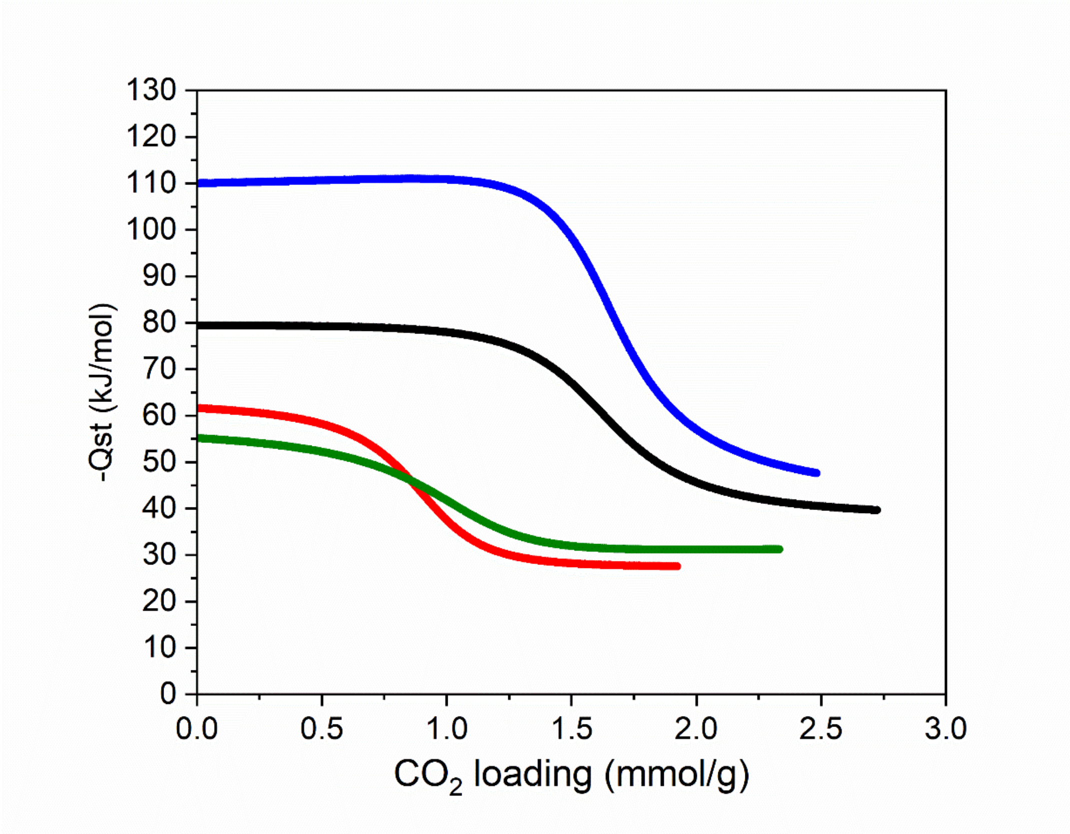

Figure S99. Heat of adsorption plots for Cr-BDC-TAEA-BDE (original from the manuscript) (blue), Cr-BDC-TAEA-BDE (75% amine) (black), Cr-BDC-TAEA-BDE (+50% epoxide) (red) and Cr-BDC-TAEA-BDE (dilute).

Table S17. Summary of CO_2 capture properties of selected porous adsorbents.

	Equilibrium measurements					Breakthrough measurements*								
**Sample**	Temp (K)	CO_2 @ 0.15bar (mmol/g)	N_2@ 0.85bar (mmol/g)	CO_2/N_2 selectivity (15/85)	Q_{st} (kJ/mol)	Sample used (g)	Temp (K)	Flow rate (ml/min)	Gas composition (% CO_2)	Breakthrough time (minutes/g)		Capacity (mmol/g)		Reference
										Dry	Humid	Dry	Humid	
Cr-BDC-TAEA-TMPTE	313	2.05	0.049	237	-124	0.3	313	2	15	87	110 (80%RH) [j]	-	-	This work
Cr-BDC-TEPA-BDE	313	1.69	0.023	416	-98	0.3	313	2	15	89	111 (80%RH) [j]	-	-	This work
Cr-BDC-TAEA-BDE	313	2.23	0.042	301	-110	0.3	313	2	15	103	142 (80%RH) [j]	-	-	This work
Cr-BDC-TEPA-TMPTE	313	1.39	0.039	202	-111	0.3	313	2	15	51	71 (80%RH) [j]	-	-	This work
mmen-Mg_2(dobpdc)	298	3.13	0.079 [c]	200 [f]	-71	-	-	-	-	-	-	-	-	2
MIL-101(Cr)-PEI-800	298	2.5	-	-	-70	-	-	-	-	-	-	-	-	3
IISERP-MOF2	303	1.6	-	1853 [g]	33	-	-	1	15	-	-	3.97	3.68 (50%RH)	4
Mg_2(dobpdc)(3-4-3) (tetraamine)	373	2.0 [a]	-	-	-99	0.67	373	30	4	-	-	-	2.0 (2.6% H_2O)	5
Cr-BDC-NH-Ac-PEI	313	1.55	0.02	437	-92	0.2	313	2	15	70	90 (80%RH) [j]	0.975	1.24 (80% RH) [j]	6
CALF-20	303	2.5	0.18	230 [h]	-39	16.7	295	-	15	-	-	-	-	7
MUF-16	293	1.2 [b]	0.059 [d]	631	-32.3	0.9	293	6	15	24		1.08		8
MOF-74-Mg-$(N_2H_4)_{1.8}$	298	5.2	-	-	-118	1.076	313	2	10	~550	~550 (82%RH)	-	-	9
Co_2Cl_2(bbta)(OH)	298	4.1	-	262	-110	1.574	313	2	10	273	281 (82%RH)	2.5	-	10
AlF	298	2.7	-	368	-47.9	-	323	2	15			0.80	0.56 (70%RH) [j]	11
MIL-120 (Al)	298	1.9 [b]	-	90	-40	-	303	1000	15	-	-	1.88(with 10% bentonite)	-	12
MUF - 17	298	1.48 [b]	0.27 [d]	270	-28.3	0.95	298	6	15	33.5	-	1.41	-	13

SIFSIX-2-Cu-i	298	1.72 [b]	0.15	140 [h]	-32	0.1–0.4	298	5	10	-	-	1.24	-	14
SIFSIX-2-Cu	298	0.22 [b]	0.17 [d]	13.7 [h]	-22	-	-	-	-	-	-	-	-	14
SIFSIX-3-Zn	298	2.38 [b]	0.22 [d]	1818 [h]	-45	0.1–0.4	298	5	1	798	-	-	-	14,15
SIFSIX-3-Cu	298	2.48	0.15 [e]	-	-54	0.1–0.4	298	5	1	1922	-	-	-	15
COF-999	298	2.34	-	-	-53	0.26	298	-	15	-	-	2.34	3.24 (75% RH) [k]	16
Zeolite 13X	303	4.49	0.34	91 [i]	-47	23.02	295	-	15	-	-	4	0.48 (1% RH) [k]	17-19
NbOFFIVE-1-Ni	298	1.3 [a]	-	-	-54.5 [l]	1	298	10	1	415	283 (74% RH)	1.86	1.27 (74% RH)	20

a = 0.04 bar CO_2

b = 0.1 bar

c = 0.75 bar

d = 1 bar

e = 0.8 bar

f = molar selectivity

g = $14CO_2$:$86N_2$ and 313 K

h = 10:90

i = competitive selectivity and 295 K

j = pre-saturation with humid gas

k = wait till water breakthrough

l = determined via TGA-DSC measurements

*It is important to note that direct comparison of breakthrough data across studies is challenging due to variations in experimental conditions such as CO_2 concentration, temperature, flow rate, sample mass, and column geometry. Additionally, the treatment of humidity differs widely, some studies pre-humidify the column, others flow humidified gas or wait for water breakthrough, making comparisons under humid conditions particularly complex. In many cases, adsorption capacities are not clearly reported. For this reason, we have intentionally omitted certain studies or left out breakthrough capacities when we judged the data to be unreliable.

References:

1. Simon, C. M.; Smit, B.; Haranczyk, M., pyIAST: Ideal adsorbed solution theory (IAST) Python package. *Comput. Phys. Commun.* **2016,** *200*, 364-380.
2. McDonald, T. M.; Lee, W. R.; Mason, J. A.; Wiers, B. M.; Hong, C. S.; Long, J. R., Capture of Carbon Dioxide from Air and Flue Gas in the Alkylamine-Appended Metal–Organic Framework mmen-Mg2(dobpdc). *J. Am. Chem. Soc.,* **2012,** *134* (16), 7056-7065.
3. Darunte, L. A.; Oetomo, A. D.; Walton, K. S.; Sholl, D. S.; Jones, C. W., Direct Air Capture of CO2 Using Amine Functionalized MIL-101(Cr). *ACS Sustainable Chem. Eng.,* **2016,** *4* (10), 5761-5768.
4. Nandi, S., Collins, S., Chakraborty, D., Banerjee, D., Thallapally, P. K., Woo, T. K., & Vaidhyanathan, R. Ultralow parasitic energy for postcombustion CO2 capture realized in a nickel isonicotinate metal–organic framework with excellent moisture stability. *J. Am. Chem. Soc.*, **2017,** *139*(5), 1734-1737.
5. Kim, E. J.; Siegelman, R. L.; Jiang, H. Z. H.; Forse, A. C.; Lee, J.-H.; Martell, J. D.; Milner, P. J.; Falkowski, J. M.; Neaton, J. B.; Reimer, J. A.; Weston, S. C.; Long, J. R., Cooperative carbon capture and steam regeneration with tetraamine-appended metal–organic frameworks. *Science,* **2020,** *369*, 392-396.
6. Justin, A.; Espín, J.; Pougin, M. J.; Stoian, D.; Schertenleib, T.; Mensi, M.; Kochetygov, I.; Ortega-Guerrero, A.; Queen, W. L., Post-Synthetic Covalent Grafting of Amines to NH2-MOF for Post-Combustion Carbon Capture. *Adv. Func. Mater.*, **2024**, *34,* 7.
7. Lin, J.-B.; Nguyen, T. T. T.; Vaidhyanathan, R.; Burner, J.; Taylor, J. M.; Durekova, H.; Akhtar, F.; Mah, R. K.; Ghaffari-Nik, O.; Marx, S.; Fylstra, N.; Iremonger, S. S.; Dawson, K. W.; Sarkar, P.; Hovington, P.; Rajendran, A.; Woo, T. K.; Shimizu, G. K. H., A scalable metal-organic framework as a durable physisorbent for carbon dioxide capture. *Science,* **2021,** *374,*1464-1469.
8. Qazvini, O. T., & Telfer, S. G. MUF-16: A robust metal–organic framework for pre-and post-combustion Carbon Dioxide capture. *ACS applied materials & interfaces*, **2021**, *13*(10), 12141-12148.
9. Liao, P. Q.; Chen, X. W.; Liu, S. Y.; Li, X. Y.; Xu, Y. T.; Tang, M.; Rui, Z.; Ji, H.; Zhang, J. P.; Chen, X. M., Putting an ultrahigh concentration of amine groups into a metal–organic framework for CO2 capture at low pressures. *Chem. Sci.* **2016**, *7*, 6528–6533.
10. Liao, P. Q.; Chen, H.; Zhou, D. D.; Liu, S. Y.; He, C. T.; Rui, Z.; Ji, H.; Zhang, J. P.; Chen, X. M., Monodentate hydroxide as a super strong yet reversible active site for CO2 capture from high humidity flue gas. *Energ. Environ. Sci.* **2015,** *8*, 1011–1016.

11. Evans, H. A.; Mullangi, D.; Deng, Z.; Wang, Y.; Bo Peh, S.; Wei, F.; Wang, J.; Brown, C. M.; Zhao, D.; Canepa, P.; Cheetham, A. K., Aluminum formate, $Al(HCOO)_3$: An earth-abundant, scalable, and highly selective material for CO_2 capture. *Sci. Adv.* **2022,** *8*, 1473.

12. Chen, B.; Fan, D.; Pinto, R. V.; Dovgaliuk, I.; Nandi, S.; Chakraborty, D.; García-Moncada, N.; Vimont, A.; McMonagle, C. J.; Bordonhos, M.; Al Mohtar, A.; Cornu, I.; Florian, P.; Heymans, N.; Daturi, M.; De Weireld, G.; Pinto, M.; Nouar, F.; Maurin, G.; Mouchaham, G.; Serre, C., A Scalable Robust Microporous Al-MOF for Post-Combustion Carbon Capture. *Adv. Sci.* **2024**, *11* (21), 2401070.

13. Qazvini, O. T.; Telfer, S. G., A robust metal–organic framework for post-combustion carbon dioxide capture. *J. Mater. Chem. A* **2020**, *8*, 12028-12034.

14. Nugent, P.; Belmabkhout, Y.; Burd, S. D.; Cairns, A. J.; Luebke, R.; Forrest, K.; Pham, T.; Ma, S.; Space, B.; Wojtas, L.; Eddaoudi, M.; Zaworotko, M. J., Porous materials with optimal adsorption thermodynamics and kinetics for CO2 separation. *Nature* **2013,** *495*, 80–84.

15. Shekhah, O.; Belmabkhout, Y.; Chen, Z.; Guillerm, V.; Cairns, A.; Adil, K.; Eddaoudi, M., Made-to-order metal-organic frameworks for trace carbon dioxide removal and air capture. *Nat. Commun.* **2014,** *5*, 4228.

16. Zhou, Z.; Ma, T.; Zhang, H; Chheda, S.; Li, H.; Wang, K.; Ehrling, S.; Giovine, R.; Li, C.; Alawadhi, A. H.; Abduljawad, M. M.; Alawad, M. O.; Gagliardi, L.; Sauer, J.; Yaghi, O. M, Carbon dioxide capture from open air using covalent organic frameworks. *Nature,* **2024,** *635*, 96–101.

17. Wilkins, N. S.; Rajendran, A., Measurement of competitive CO_2 and N_2 adsorption on Zeolite 13X for post-combustion CO_2 capture. *Adsorption,* **2019,** *25*, 115–133.

18. Wilkins, N. S., Sawada, J. A., & Rajendran, A. Measurement of competitive CO2 and H2O adsorption on zeolite 13X for post-combustion CO 2 capture. *Adsorption*, **2020,** 26(5), 765-779.

19. Mondino, G.; Grande, C. A.; Blom, R.; Nord, L. O., Moving bed temperature swing adsorption for CO2 capture from a natural gas combined cycle power plant. *Int. J. Greenh. Gas Control*, **2019,** 85, 58-70.

20. Bhatt, P. M.; Belmabkhout, Y.; Cadiau, A.; Adil, K.; Shekhah, O.; Shkurenko, A.; Barbour, L.J.; Eddaoudi, M., A Fine-Tuned Fluorinated MOF Addresses the Needs for Trace CO2 Removal and Air Capture Using Physisorption. *J. Am. Chem. Soc.,* **2016**, *138* (29), 9301-9307.
